# What and where? Predicting invasion hotspots in the Arctic marine realm

**DOI:** 10.1111/gcb.15159

**Published:** 2020-07-10

**Authors:** Jesica Goldsmit, Christopher W. McKindsey, Robert W. Schlegel, D. Bruce Stewart, Philippe Archambault, Kimberly L. Howland

**Affiliations:** ^1^ Fisheries and Oceans Canada Maurice Lamontagne Institute Mont‐Joli QC Canada; ^2^ Department of Biology, Science and Engineering Faculty ArcticNet Takuvik Laval University Quebec City QC Canada; ^3^ Fisheries and Oceans Canada Arctic Research Division Freshwater Institute Winnipeg MB Canada; ^4^ Department of Oceanography Dalhousie University Halifax NS Canada; ^5^ Arctic Biological Consultants Winnipeg MB Canada

**Keywords:** aquatic invasive species, climate warming, ensemble models, habitat suitability, risk of invasion, shipping, species distribution model

## Abstract

The risk of aquatic invasions in the Arctic is expected to increase with climate warming, greater shipping activity and resource exploitation in the region. Planktonic and benthic marine aquatic invasive species (AIS) with the greatest potential for invasion and impact in the Canadian Arctic were identified and the 23 riskiest species were modelled to predict their potential spatial distributions at pan‐Arctic and global scales. Modelling was conducted under present environmental conditions and two intermediate future (2050 and 2100) global warming scenarios. Invasion hotspots—regions of the Arctic where habitat is predicted to be suitable for a high number of potential AIS—were located in Hudson Bay, Northern Grand Banks/Labrador, Chukchi/Eastern Bering seas and Barents/White seas, suggesting that these regions could be more vulnerable to invasions. Globally, both benthic and planktonic organisms showed a future poleward shift in suitable habitat. At a pan‐Arctic scale, all organisms showed suitable habitat gains under future conditions. However, at the global scale, habitat loss was predicted in more tropical regions for some taxa, particularly most planktonic species. Results from the present study can help prioritize management efforts in the face of climate change in the Arctic marine ecosystem. Moreover, this particular approach provides information to identify present and future high‐risk areas for AIS in response to global warming.

## Graphical abstract

Marine aquatic invasive species with the greatest potential for invasion and impact in the Canadian Arctic were identified and modelled to predict their potential spatial distributions at pan‐Arctic and global scales. Modelling for 23 species was conducted under present environmental conditions and two future (2050 and 2100) global warming scenarios. Potential invasion hotspots were identified for Hudson Bay, Northern Grand Banks/Labrador, Chukchi/Eastern Bering seas and Barents/White seas, suggesting that these regions are most vulnerable to invasions. Organisms showed future poleward shifts and overall gains in habitat suitability under future conditions, although some species distributions will likely recede towards the equator.

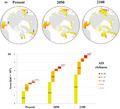

## 1 INTRODUCTION

Invasive species are considered to be one of the main drivers of biodiversity loss and recent extinctions (Bellard, Cassey, & Blackburn, 2016; Blackburn, Bellard, & Ricciardi, 2019). In addition, they may cause great ecological and economic impacts and, in some cases, even impact human health (Cook, Payne, MacLeod, & Brown, 2016; Hewitt, Campbell, & Gollasch, 2006; Ricciardi, Hoopes, Marchetti, & Lockwood, 2013; Simberloff et al., 2013). Estimates suggest that their presence may entail costs of up to 12% of the gross domestic product of affected countries (Marbuah, Gren, & McKie, 2014).

The greatest numbers of aquatic invasions have been documented in temperate regions (Ruiz & Hewitt, 2009). However, high latitude areas are generally warming at a faster rate than other areas (Niemi et al., 2019; Overland et al., 2019) and various climate change scenarios predict that the ice‐free season will continue to lengthen (Barnhart, Miller, Overeem, & Kay, 2016; Jahn, 2018; Niederdrenk & Notz, 2018; Sigmond, Fyfe, & Swart, 2018). If warming is limited to 1.5°C, as proposed in the Paris Agreement, ice‐free summers in the Arctic will continue to rise even if global warming ceases (Screen, 2018). Moreover, temperature extremes at regional scales could be much greater than the global mean with some models predicting a rise greater than 7°C in the Arctic with a global average increase of only 1.5°C at peak warming (Seneviratne et al., 2018). Aquatic invasions in Arctic ecosystems have thus been identified as an emerging issue (Ricciardi et al., 2017) as the area is becoming increasingly susceptible due to regional climate change and associated increases in shipping activity and resource exploitation (Melia, Haines, & Hawkins, 2016; Miller & Ruiz, 2014; Niimi, 2004; Smith & Stephenson, 2013).

One of the leading causes of increasing invasion risk is the expansion of transportation networks (Early et al., 2016). Aquatic transportation, in particular, is highly relevant given that the shipping network is responsible for 90% of global trade (Kaluza, Kölzsch, Gastner, & Blasius, 2010; Xu et al., 2014), with ballast water and biofouling being the main vectors for transport of organisms (Bailey, 2015; Drake & Lodge, 2007; Ruiz, Fofonoff, Steves, & Carlton, 2015). Consequently, marine taxa are one of the dominant groups that are unintentionally introduced at a global scale (Turbelin, Malamud, & Francis, 2017), and are predicted to contribute a 3‐ to 20‐fold increase in global invasion risk (Sardain, Sardain, & Leung, 2019). In the Arctic, almost half of known marine invasions have been due to shipping activities as a single‐pathway means of transport (Chan et al., 2019). Greater opportunities for aquatic invasive species (AIS) transport are expected in the future as shipping traffic is projected to grow given predicted ice‐free conditions that will allow more direct shipping corridors between the Pacific and the Atlantic oceans (Melia et al., 2016; Miller & Ruiz, 2014; Smith & Stephenson, 2013), reducing transit distances by up to 24% relative to current shipping routes (Buixadé Farré et al., 2014).

A recent study tallied a total of 34 non‐indigenous species for the entire Arctic, with shipping activity being one of the main purported vectors (Chan et al., 2019). In addition, non‐indigenous species have been identified from both ships en route to the Arctic and in the environment to which they arrive, highlighting the risk of new invasions in this region (Ashton, Riedlecker, & Ruiz, 2008; Chan, MacIsaac, & Bailey, 2015; Dispas, 2019; Gíslason et al., 2014; Golder, 2018; Laget, 2017; Lambert, Shenkar, & Swalla, 2010; Svavarsson & Dungal, 2008; Tremblay, 2017; Zimina, 2014). Furthermore, the origins of many newly observed species in Arctic locations are unknown so they have often been classified as cryptogenic (Goldsmit, Howland, & Archambault, 2014; MacDonald, Bluhm, Iken, Gagaev, & Strong, 2010). These numbers are high compared to the other polar environment—the Antarctic—where there are no established AIS populations to date and only five non‐indigenous marine species that have potentially arrived through human activities (McCarthy, Peck, Hughes, & Aldridge, 2019). However, Hughes et al. (2020) suggest that marine taxa pose the greatest potential invasion risk to the Antarctic Peninsula.

Given the vast size and remoteness of the Arctic, few baseline studies have been conducted (Archambault et al., 2010; CAFF & PAME, 2017; Piepenburg et al., 2011) and the resident fauna are thus poorly described relative to more accessible locations elsewhere in the world (Costello et al., 2017). Consequently, simply identifying newly arriving species can be challenging. To improve this detection gap, new methods and identification techniques are being used in remote regions, such as environmental DNA and metabarcoding (Brown, Chain, Zhan, MacIsaac, & Cristescu, 2016; Chain, Brown, MacIsaac, & Cristescu, 2016; Lacoursière‐Roussel et al., 2018; Leduc et al., 2019). Species distribution modelling (SDM) may be used to predict the distribution of suitable habitat for AIS (Barbosa & Schneck, 2015; Barbosa, Schneck, & Melo, 2012), including for high‐risk AIS in areas of concern, such as high latitude regions (Byrne, Gall, Wolfe, & Agüera, 2016; de Rivera, Steves, Fofonoff, Hines, & Ruiz, 2011; Goldsmit et al., 2018; Ware et al., 2016). Modelled results can inform preinvasion management policies to avoid new arrivals and, potentially, subsequent species establishments and consequent needs for eradication actions (Floerl, 2014; Locke & Hanson, 2009).

Although future invasions are expected to be enhanced by climate change (Bellard et al., 2013), predicted SDM‐modelled distributions of environmental suitability for invasive species have been understudied in this context (Bellard, Jeschke, Leroy, & Mace, 2018), particularly for marine ecosystems (Barbosa et al., 2012; Bellard et al., 2018). Nevertheless, SDM tools have been successfully applied to marine species (Lowen, McKindsey, Therriault, & DiBacco, 2016; Meißner et al., 2014; Reiss, Cunze, König, Neumann, & Kröncke, 2011; Robinson et al., 2011; Robinson, Nelson, Costello, Sutherland, & Lundquist, 2017; Weinert et al., 2016), including forecasting distributions under global change scenarios in high latitude regions (de Rivera et al., 2011; Goldsmit et al., 2018; Jueterbock, Smolina, Coyer, & Hoarau, 2016).

Regions where many species coincide, known as biodiversity hotspots, may provide information about species richness, endemism and/or threatened taxa, and may be of importance for conservation (Myers, Mittermeier, Mittermeier, Da Fonseca, & Kent, 2000). Based on this concept, potential invasion hotspots may be identified as areas that are hospitable to and thus at risk of invasion by exceptionally high numbers of AIS for a given region (Ruiz, Fofonoff, Steves, Foss, & Shiba, 2011). The use of a biological invasion hotspot approach has been limited to date (but see Bellard, Leroy, Thuiller, Rysman, & Courchamp, 2016; Catford, Vesk, White, & Wintle, 2011; Ibanez, Silander, Allen, Treanor, & Wilson, 2009; O'Donnell et al., 2012; Torres et al., 2018); with few studies in marine environments. Most analyses of AIS hotspots in marine ecosystems have focused on vectors (Davidson et al., 2010; Drake & Lodge, 2004; Pearce, Peeler, & Stebbing, 2012; Semmens, Buhle, Salomon, & Pattengill‐Semmens, 2004; Tidbury, Taylor, Copp, Garnacho, & Stebbing, 2016) and current AIS richness (Edelist, Rilov, Golani, Carlton, & Spanier, 2013; Katsanevakis et al., 2014; Kelly, Leach, Cameron, Maggs, & Reid, 2014; Rilov & Galil, 2009; Ruiz et al., 2011; Verlaque, 2001), although a few have also predicted hotspots of potential marine biological invasions (Cheung et al., 2009; Gallardo, Zieritz, & Aldridge, 2015; Jones & Cheung, 2015).

This study aims to identify potential high‐risk AIS and predict hotspots of invasion for those under current and projected future environmental conditions in Arctic environments. Cold‐tolerant planktonic and benthic AIS were scored to produce a list of the top species with the highest relative likelihood of invasion and impact, since these ecological groups include the dominant known highest risk marine invasive species (Molnar, Gamboa, Revenga, & Spalding, 2008). The distribution of suitable habitats for this set of species was then modelled to predict regions of overlap under current and future projected conditions, thus identifying hotspots of potential biological invasions. Although all analyses were done globally, there was an emphasis at the Canadian and pan‐Arctic scales.

## 2 MATERIALS AND METHODS

### 2.1 Study region

**FIGURE 1:**
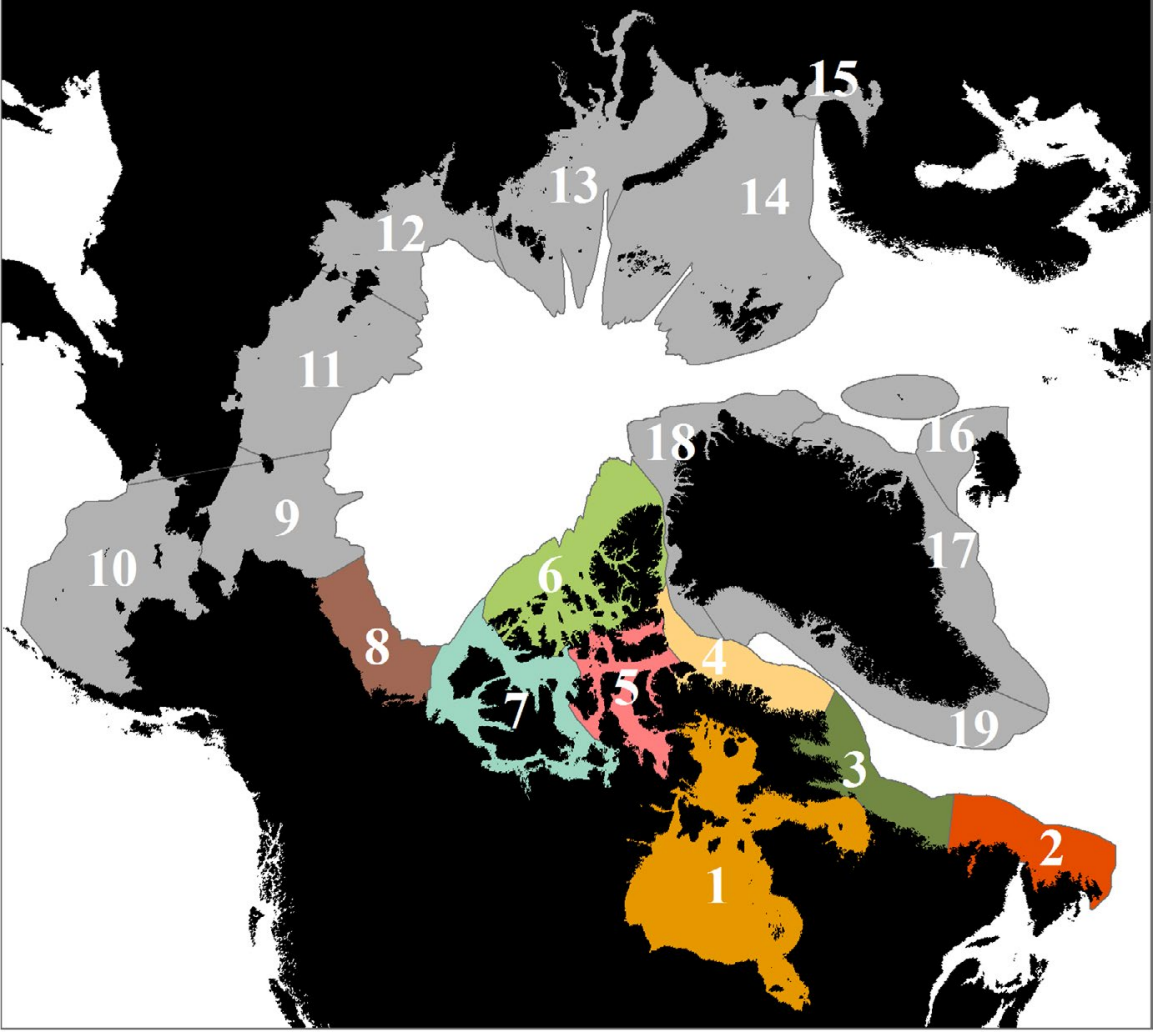
Arctic ecoregions as delineated by Spalding et al. (2007). Canadian Arctic ecoregions are coloured. 1, Hudson Bay Complex; 2, Northern Grand Banks‐Southern Labrador; 3, Northern Labrador; 4, Baffin Bay‐Davis Strait; 5, Lancaster Sound; 6, High Arctic Archipelago; 7, Beaufort‐Amundsen‐Viscount Melville‐Queen Maud; 8, Beaufort Sea‐continental coast and shelf; 9, Chukchi Sea; 10, Eastern Bering Strait; 11, East Siberian Sea; 12, Laptev Sea; 13, Kara Sea; 14, North and East Barents Sea; 15, White Sea; 16, North and East Iceland; 17, East Greenland Shelf; 18, North Greenland; 19, West Greenland Shelf

Distribution model outputs were produced with global coverage to allow evaluation of global and pan‐Arctic patterns although analyses were focussed on the potential highest‐risk species for the Canadian Arctic. The rationale for this approach is the vast expanse of the Canadian Arctic (Archambault et al., 2010), its vulnerability to invasion (Chan, Bailey, Wiley, & MacIsaac, 2013; Chan, MacIsaac, & Bailey, 2016; Goldsmit et al., 2018; Goldsmit, McKindsey, Archambault, & Howland, 2019), the ambiguous status of new species that have potentially arrived through human transport (Goldsmit et al., 2014) and the unprecedented warming that it has experienced over the last decade (Bush & Lemmen, 2019; Niemi et al., 2019). The region is immense, accounting for eight of the 19 Arctic marine ecoregions (Figure 1; Spalding et al., 2007). In fact, Canada has the longest coastline in the world, the majority of which is situated in the Arctic (accounting for almost 2,000,000 km^2^ of the territorial sea; Archambault et al., 2010). Even though few AIS have been identified in Canada relative to other Arctic regions, Chan et al. (2019) suggest that this accounts for ca. 20% of all AIS recorded from marine Arctic waters. Recently, additional species have been identified for the first time in the Canadian Arctic using molecular tools (Brown et al., 2016; Chain et al., 2016; Lacoursière‐Roussel et al., 2018) or identified as cryptogenic as their origin is unclear (Goldsmit et al., 2014). Moreover, Canadian Arctic ports (especially those in the Hudson Bay Complex; Figure 1) have been identified as being of moderate to high ecological risk of invasion since they could provide suitable habitat for various AIS (Goldsmit et al., 2018; Goldsmit, Nudds, et al., 2019) and there is evidence that a number of non‐indigenous species are being transported into the region by shipping (Chan et al., 2015; Dispas, 2019; Laget, 2017; Tremblay, 2017).

### 2.2 Species selection

A three‐step procedure was used to select potential marine AIS (zoobenthos, phytobenthos and zooplankton) for modelling (Figure S1). Most were species that are considered AIS in other regions of the world and may be transported via shipping to the Canadian Arctic; several others had been previously detected in other Arctic environments. These steps included: (a) prescreening analysis and selection; (b) ranking a subset of these species using an invasive species screening tool; and (c) selecting a final list of higher risk species based on tool results.

The first step of the prescreening analysis was to identify potential Arctic AIS. One hundred species were identified using a combination of data sources including published articles, grey literature and web‐based global invasive species databases (see details in Figure S1.1). Species' biological and ecological characteristics for survival in Arctic conditions (e.g. temperature and salinity tolerances) and their ability to arrive in the region via shipping were evaluated, identifying a total of 31 potential Arctic AIS (Figure S1.2a). These species were then ranked to evaluate invasion risk using the Canadian Marine Invasive Species Tool (CMIST; Drolet et al., 2016)—a rapid screening‐level risk assessment tool to quantify the risk of existing or potential marine invaders in a given area. The semiquantitative tool uses existing information and expert opinion to evaluate the potential for arrival, establishment, spread and impact by a given species and has been applied in a number of eco‐regions within Canada (DFO, 2017; Drolet et al., 2016; Moore, Lowen, & DiBacco, 2018; Therriault et al., 2018). CMIST scores are computed based on responses to 17 questions related to the likelihood and impact of invasion (see Figure S1.2a) according to the information found in the literature and other sources. Expert assessor knowledge on the risk assessment areas was used to score the potential impact of a given species based on its known impacts observed elsewhere and the availability of suitable habitats and environmental conditions. Uncertainty is scored for each risk question by assigning a qualitative score reflecting the quality of information available to answer each CMIST question. A Monte Carlo randomization procedure is then used to obtain adjusted risk scores that include uncertainty (Drolet et al., 2016).

CMIST‐ranked species with either medium or high mean risk scores (*N* = 18; Table 1) were retained for more detailed assessment using SDM. To this list, five potentially harmful phytoplankton species were added (Table 1; Figure S1.2b). These dinoflagellate species have been found in ballast water tanks and/or in ballast water exchange zones of Canadian domestic ships that discharge their ballast water into Canadian Arctic ports (Laget, 2017). All are known to have the capacity to produce toxins and have been implicated in harmful algal events throughout the world (Harmful Algal Information System, from the Intergovernmental Oceanographic Commission of UNESCO, http://haedat.iode.org/).

**TABLE 1:** List of modelled species and the methodology for species selection. Ecological groups were classified as zoobenthos, phytobenthos, zooplankton and phytoplankton. Selection methods were the Canadian Marine Invasive Screening Tool (CMIST; Drolet et al., 2016), and harmful dinoflagellate species found in ballast of vessels discharging in the Canadian Arctic (Laget, 2017)

Species	Common name	Taxa	Ecological group	Selection method	Predictors included in SDM
*Amphibalanus eburneus*	Ivory barnacle	Crustacea	Zoobenthos	CMIST	Bottom temperature Sea surface temperature Bottom salinity Sea surface salinity Ice thickness Depth Distance to land
*Botrylloides violaceus*	Violet tunicate	Tunicata	Zoobenthos	CMIST	
*Botryllus schlosseri*	Golden star tunicate	Tunicata	Zoobenthos	CMIST	
*Carcinus maenas*	Green crab	Crustacea	Zoobenthos	CMIST	
*Chionoecetes opilio*	Snow crab	Crustacea	Zoobenthos	CMIST	
*Ciona intestinalis*	Vase tunicate	Tunicata	Zoobenthos	CMIST	
*Littorina littorea*	Common periwinkle	Mollusca	Zoobenthos	CMIST	
*Membranipora membranacea*	Coffin box bryozoan	Bryozoa	Zoobenthos	CMIST	
*Molgula manhattensis*	Sea grape	Tunicata	Zoobenthos	CMIST	
*Mya arenaria*	Soft shell clam	Mollusca	Zoobenthos	CMIST	
*Paralithodes camtschaticus*	Red king crab	Crustacea	Zoobenthos	CMIST	
*Codium fragile* spp. *fragile*	Dead man's fingers	Chlorophyta	Phytobenthos	CMIST	Bottom temperature Sea surface temperature Bottom salinity Sea surface salinity Ice thickness Depth Distance to land Photosynthetically active radiation Dissolved oxygen pH Minerals and nutrients (calcite, iron, nitrate, phosphate and silicate)
*Dumontia contorta*	Dumont's tubular weed	Rhodophyta	Phytobenthos	CMIST	
*Sargassum muticum*	Japanese wireweed	Phaeophycea	Phytobenthos	CMIST	
*Undaria pinnatifida*	Wakame	Phaeophycea	Phytobenthos	CMIST	
*Acartia (Acanthacartia) tonsa*	No common name found	Copepoda	Zooplankton	CMIST	Sea surface temperature Bottom salinity Sea surface salinity Ice thickness Depth Distance to land Chlorophyll concentration Dissolved oxygen pH
*Aurelia limbata*	Brown‐branded moon jelly	Cnidaria	Zooplankton	CMIST	
*Mnemiopsis leidyi*	Warty comb jelly	Ctenophora	Zooplankton	CMIST	
*Alexandrium tamarense*	No common name found	Dinoflagellata	Phytoplankton	Laget (2017)	Sea surface temperature Sea surface salinity Ice thickness Distance to land Photosynthetically active radiation Dissolved oxygen pH Minerals and nutrients (iron, nitrate, phosphate and silicate)
*Dinophysis caudata*	No common name found	Dinoflagellata	Phytoplankton	Laget (2017)	
*Dinophysis dens*	No common name found	Dinoflagellata	Phytoplankton	Laget (2017)	
*Gonyaulax polygramma*	No common name found	Dinoflagellata	Phytoplankton	Laget (2017)	
*Kryptoperidinium triquetrum*	No common name found	Dinoflagellata	Phytoplankton	Laget (2017)	

The final species list for modelling thus included a total of 23 known marine AIS or harmful algal species from four ecological groups (zoobenthos, phytobenthos, zooplankton and phytoplankton) that pose potential risks for invasion in the Canadian Arctic. To simplify terminology, hereafter when referring to this suite of species they will be termed as ‘AIS’. (See Table S1 for information on species' characteristics and impacts). Likewise, the term ‘invasion’ is used to make reference to the complete process of a species transitioning all invasion stages (transport, arrival, establishment and spread; Lockwood, Hoopes, & Marchetti, 2007).

### 2.3 Species data

Global scale occurrence data of marine invaders selected for modelling was compiled for both native and invaded ranges using global biodiversity databases such as the Global Biodiversity Information Facility (GBIF—www.gbif.org), Ocean Biogeographic Information System (OBIS—www.obis.org), and invasive species lists with coordinate location information and specific literature (Table S1). An effort was made to maximize the number and quality of occurrence records to best predict potential distributions by doing a vast and complete search of occurrence records and by checking information to find the original source of those records (García‐Roselló et al., 2015; Guisan, Graham, Elith, & Huettmann, 2007; Guisan, Zimmermann, et al., 2007; Lobo, 2008). Both native and invaded ranges were used for training and evaluating all models (Verbruggen et al., 2013), since invaded areas provide valuable information on species' tolerances to climatic conditions that may not be present in their native range (Marcelino & Verbruggen, 2015) and may improve predictions for the extent of suitable habitat (Broennimann & Guisan, 2008). A single presence record was counted per grid cell to decrease the possibility of overprediction, hence, occurrence points were considered at the same resolution as the corresponding environmental layers (García‐Roselló et al., 2015). All points were verified to ensure that they were in sea grids (and not over land). When necessary, points were moved to the closest sea grid using the Near Proximity tool in ArcMap v10.2.2.

### 2.4 Environmental data

Marine data layers, prepared specifically for ecological modelling, were used as environmental predictors and downloaded at a global scale from Bio‐ORACLE v2 (http://www.bio‐oracle.org/). These layers were produced with climate data describing monthly averages for the period from 2000 to 2014 representing recent (hereafter referred to as present) conditions (Assis et al., 2018). They were obtained from preprocessed global ocean re‐analyses combining satellite and in situ observations at regular two‐ and three‐dimensional spatial grids (Assis et al., 2018). A set of 37 environmental layers was used from this source comprising bottom and sea surface temperature and salinity, ice thickness, chlorophyll, dissolved oxygen, pH, photosynthetically active radiation and minerals and nutrients (iron, calcite, nitrate, phosphate and silicate; Table 1; Table S2). Long‐term maximum, minimum and mean values were used for most predictors when available (Table S2). Resolution of environmental layers was 5 arcmin (approximate 9.2 km at the equator). Bathymetry and land distance layers were obtained from Aquamaps (http://www.aquamaps.org/; Kaschner et al., 2016), but with a resolution of 30 arcmin (approximately 55 km at the equator; Table S2). Bottom substratum type was not included in the analysis due to the lack of availability of a high‐resolution global scale database; suitable benthic habitat was assumed to be present within each study region.

### 2.5 Habitat suitability model

Habitat suitability for selected AIS was modelled with MaxEnt v3.3.3k (Phillips, Anderson, & Schapire, 2006) and biomod2 v3.4.6 (Thuiller, Georges, Engler, & Breiner, 2020) within R v3.6.3 (R Core Team, 2019). Multiple modelling techniques were used so that the results from each approach could be combined into an ensemble model, which has been shown to reduce the biases that single models may have (Araújo & New, 2007).

MaxEnt is a machine learning method based on maximum entropy that predicts the potential geographic distribution of suitable habitat for species using species occurrence data and various combinations of environmental data layers as input. The method is one of the most widely used SDM algorithms due to its high predictive accuracy and efficiency in modelling range shifts under future climate change scenarios (Bucklin et al., 2015; Elith et al., 2006, 2011; Hijmans & Graham, 2006; Pearson, 2007). It has also recently been shown to outperform other modelling techniques in accurately predicting invasive species distributions (Battini, Farías, Giachetti, Schwindt, & Bortolus, 2019). This was complemented with SDM using a suite of other techniques within biomod2, another well‐known and widely used SDM tool (Hao, Elith, Guillera‐Arroita, & Lahoz‐Monfort, 2019) that was selected because it allows multiple models to be run on the same training and testing datasets to better ensure the consistency and accuracy of resultant ensemble models. The four models were run with biomod2: generalized linear model (GLM), random forest (RF), artificial neural network and generalized additive model (GAM) because each represents a fundamentally different modelling technique. Initially, BIOCLIM (a presence‐only model) was also included in runs but was later removed as its performance was very low, in agreement with other studies (Elith et al., 2006), and is thus the least frequently used SDM approach within biomod2 (Hao et al., 2019). More detailed information about each of these models included from biomod2 may be found in Thuiller, Lafourcade, Engler, and Araújo (2009).

MaxEnt generates background data to compare with known presence points. This study used the default option, which generates 10,000 random background points. In contrast, selected models from biomod2 require presence and absence data, hence it was necessary to create pseudo‐absence values before running the biomod2 models (Thuiller et al., 2020). For consistency with MaxEnt models, 10,000 pseudo‐absence points were selected randomly using the biomod2 default settings.

Model predictive power for MaxEnt and biomod2 was evaluated using cross‐validation with 70% of the occurrence points chosen randomly and used to train the model and the other 30% to test the complete set of models (Araújo, Pearson, Thuiller, & Erhard, 2005). For MaxEnt, data were partitioned by a random process of *k* = 500 training and validation iterations (Hijmans, 2012), and for biomod2 the data were partitioned randomly. Training points for MaxEnt were selected by random seeding with the convergence threshold set at 0.00001. The hinge feature in MaxEnt was used as it produces complex yet smooth and ecologically meaningful response curves and improves model performance (Merow, Smith, & Silander, 2013; Phillips & Dudík, 2008).

Environmental predictors for each species were selected based on initial models run with MaxEnt, which has been shown to have good discrimination accuracy regarding the identification of important variables compared to other models (Smith & Santos, 2019). Selected predictors included those that are most typically considered as important for related taxa and have been used in other modelling studies (Table 1; Barnes, 1999; Belanger et al., 2012; Chust et al., 2016; Cusson, Archambault, & Aitken, 2007; Gallardo et al., 2015; Jensen, Mousing, & Richardson, 2017; Leidenberger, Obst, et al., 2015; Wagner, 1977), or were identified by taxonomic experts as being important (G. Winkler, personal communication, 2017; A. Rochon, personal communication, 2018). After the first run, predictors with a relative contribution score of <4% were excluded (Jueterbock et al., 2016). Correlations may lead models to produce erroneous response curves to predictors that do not reflect species physiological tolerances (Marcelino & Verbruggen, 2015). Thus, highly correlated environmental predictors (correlation coefficient ≥ 0.7; Dormann et al., 2013), as determined using the SDMtoolbox (Brown, 2014; Table S2), were evaluated and the one with the highest contribution to the modelling exercise retained for model construction. As per Goldsmit et al. (2018), environmental predictors were identified by evaluating the combination of: (a) the response curves for each species—to evaluate if the predictor behaves in a biologically meaningful way in the model (Marcelino & Verbruggen, 2015); (b) a species‐specific Jackknife test—to evaluate the contributions of the various parameters and analyse the importance of predictors; and (c) the estimates of the contribution of each environmental layer to model prediction. A minimum of three environmental predictors were included in each model. Each species was then modelled again with the selected environmental predictors (Table S3). Model performance was evaluated as the area under the curve (AUC) of the receiver operating curve and true skill statistic (TSS). AUC is the probability that the model correctly ranks a random presence site versus a random site from the study area (Phillips et al., 2009). Values close to 1 indicate good prediction in site discrimination, a value of 0.5 indicates a prediction equal to random and values lower than 0.5 indicate a performance that is worse than random predictions. TSS assesses the accuracy of predictions using sensitivity (proportion of correctly predicted presences) and specificity (proportion of correctly predicted absences) in its calculation (TSS = sensitivity + specificity − 1) and is an appropriate evaluation alternative for model predictions converted to binary maps using a threshold (Allouche, Tsoar, & Kadmon, 2006). It ranges from −1 to +1, where values between 0 and −1 indicate performance no better than random, while a statistically reliable model performance is indicated by values >0.4, excellent models by a minimum of 0.7, and 1 indicates perfect agreement with the model (Allouche et al., 2006). Models with a TSS score of <0.7 and sensitivity = 0 were excluded from the final ensemble predictions.

MaxEnt and biomod2 models were each run five times. The continuous values produced by the models were transformed to binary values to identify predicted suitable and unsuitable habitat since continuous model projections may be difficult to interpret. In addition, binary presence/absence maps are more useful for risk assessment exercises and are required for some model evaluations (Liu, White, & Newell, 2013). For each species, the binary results for the five runs per model were averaged to create a single binary result per model. The resulting binary results for each model were then averaged to produce the final ensemble binary projection for each species. Binary transformation was done using the maximum training sensitivity plus specificity threshold, which maximizes TSS values to create binary maps and has been shown to produce the most accurate predictions (Andrade, Velazco, & Júnior, 2020; Jiménez‐Valverde & Lobo, 2007; Liu et al., 2013), and has been used in other studies with similar objectives (e.g. Bellard et al., 2013; Duffy et al., 2017; Wisz et al., 2015). Following transformation, all binary models were delimited using a threshold for the maximum depth each species could inhabit according to their ecological requirements (Goldsmit et al., 2018; Table S4). An exception was made for phytoplankton as they were all surface dinoflagellates; thus, there was no need to consider maximum depth for their distribution (A. Rochon, personal communication, 2018). Heat maps showing the total number of modelled AIS that may find suitable habitat in a region—hereafter ‘AIS richness’—were then created using combined maps representing the cumulative number of species (of the 23 modelled) predicted to find suitable habitat in a given grid cell at global and pan‐Arctic scales. It should be kept in mind that, at a global scale, richness includes potential native and invaded ranges of all species modelled, while at the pan‐Arctic scale, richness includes mainly predicted potentially suitable habitats.

### 2.6 Future projections

Once all models were run, tested and validated, predicted habitat suitability was evaluated under projected global change scenarios at global and pan‐Arctic scales. The same set of environmental layers that were used for contemporary models were used for projected future models although only temperature, salinity and ice layers were available for projected future scenarios (Table S3). This is common practice in similar modelling exercises (e.g. Goldsmit et al., 2018; Jueterbock et al., 2016; Leidenberger, De Giovanni, Kulawik, Williams, & Bourlat, 2015; Weinert et al., 2016). Future environmental layers were obtained from Bio‐ORACLE v2 for RCP4.5 emission scenario for the years 2050 and 2100. The average and range of climatic anomalies for temperature, salinity and sea ice thickness of this scenario at the end of the century are given in Assis et al. (2018). In short, it represents an intermediate greenhouse emission (temperature anomaly of 2.4°C by 2100; Moss et al., 2008) and stabilization scenario resulting from the implementation of appropriate mitigation strategies (Clarke et al., 2007). This anomaly also coincides approximately with the expected increase in temperature in Arctic regions under the proposed efforts of the 2015 Paris Agreement (Solecki et al., 2018). Future layers were produced for 2040–2050 and 2090–2100 by averaging data from distinct atmosphere–ocean general circulation models provided by the Coupled Model Intercomparison Project 5, which was developed by the World Climate Research Programme's Working Group on Coupled Modelling (see Assis et al., 2018 for more information).

Resulting distribution models of each individual species were compiled by ecological groups (zoobenthos, phytobenthos, zooplankton and phytoplankton) and these combined models were used to compare present with forecasted distributions for the years 2050 and 2100. This was achieved by spatially analysing species richness and distribution change between present and future climate scenarios using ArcMap v10.2.2 and SDMtoolbox (Brown, 2014). Overlap of regions was analysed at both global and pan‐Arctic scales, to evaluate predicted latitudinal shifts in suitable habitat. Changes in the distribution of suitable habitat over time were evaluated independent of natural or anthropogenic habitat overlap to identify regions of loss, gain and no change in time, for both time frames.

## 3 RESULTS

### 3.1 Analysis of environmental predictors and model evaluation

**FIGURE 2:**
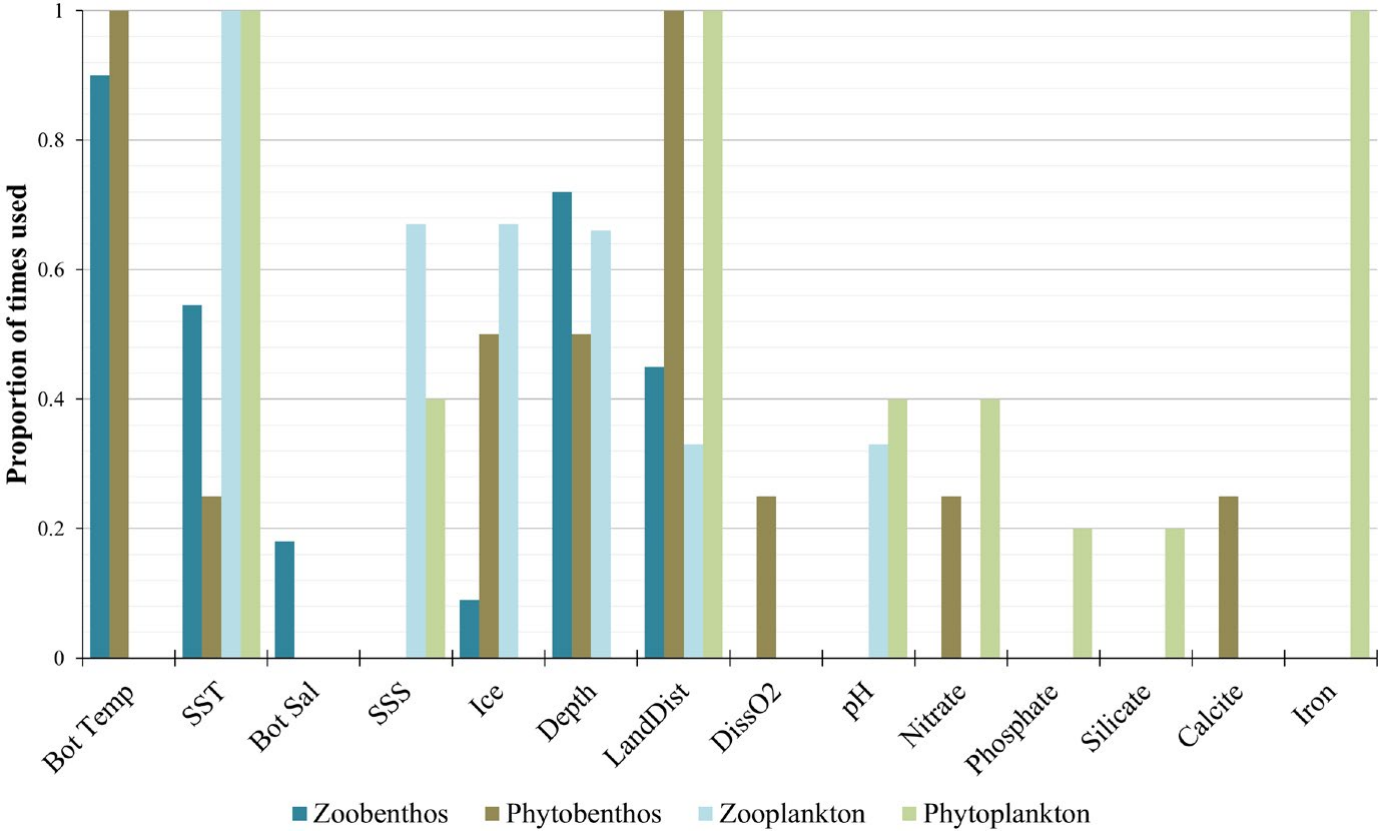
Proportion of times individual predictors were used for model building per ecological group. Bot Sal, bottom salinity; Bot Temp, bottom temperature; DissO_2_, dissolved oxygen; LandDist, land distance; SSS, sea surface salinity; SST, sea surface temperature

Varying combinations of environmental predictors contributed to explaining the species distribution models of the ecological groups analysed (Figure 2). Sea surface temperature and land distance contributed to models for all ecological groups, although in varying proportions. Bottom temperature and depth were important in explaining models for zoobenthos and phytobenthos, in addition to ice thickness for the latter. Other main predictors for zooplankton included sea surface salinity, ice and depth, whereas important phytoplankton predictors included nutrients and minerals (especially iron; Figure 2; Table S3). All environmental predictors used to construct the final model for each species were within training ranges. Analysis of unimodal environmental response curves indicated that environmental conditions were within suitable ranges for all modelled species.

After averaging the five replicates of each model, most AUC values were >0.9 and TSS > 0.8, indicating good model performance and good prediction in site discrimination with most of the models included in the ensemble results. There were only four species with somewhat lower AUC or TSS values for some of the averaged models (*Chionoecetes opilio*, *Dinophysis caudata* and *Gonyaulax polygramma* with AUC between 0.76 and 0.83 in MaxEnt models; and *G. polygramma* and *Aurelia limbata* with TSS between 0.75 and 0.79 in GAM, GLM and Maxent models), but still well above the thresholds for random site prediction (Table S5). A few individual replicated GAM, GLM and RF models (*N* = 19) for five species were excluded from model‐specific binary outputs (maximum 3/species) due to their having TSS < 0.7 and/or sensitivity = 0; which resulted in model averages based on fewer than five replicates for these species (Table S5).

### 3.2 Hotspots vulnerable to invasions

**FIGURE 3:**
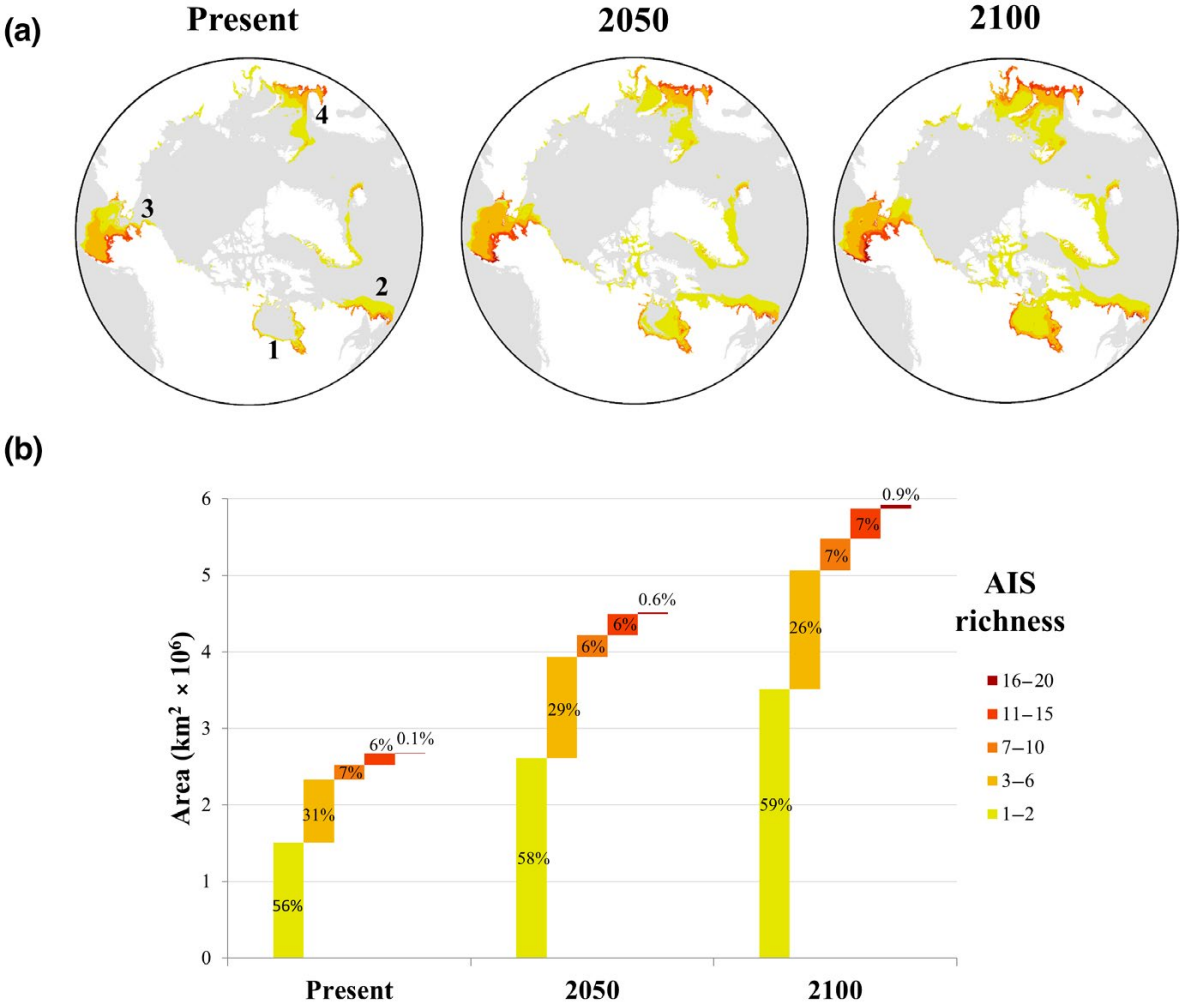
Predicted total aquatic invasive species (AIS) richness at an Arctic scale: (a) predicted hotspots of AIS richness for present and future (2050 and 2100) conditions in the Arctic (1, Hudson Bay; 2, Northern Grand Banks/Labrador; 3, Chukchi/Eastern Bering seas; 4, Barents and White seas). Colours represent the number of overlapping species with predicted suitable habitat in a given area; (b) predicted future extension of suitable habitat by area for each category (natural breaks Jenks) of AIS richness. Values shown in the bars are the net percentages of suitable habitat at each level of AIS richness within each projected time period

From the 23 modelled species, up to 20 were predicted to have overlapping suitable habitat in Arctic regions, mostly located near the coasts surrounding Hudson Bay, Northern Grand Banks/Labrador, Chukchi/Eastern Bering seas and Barents/White seas (Figure 3a). These hotspots are predicted to have potentially greater AIS richness compared to other Arctic regions in the present and both modelled future scenarios. The areal extent of hotspots vulnerable to invasion is predicted to increase over time; this increase is predicted not only in total area, but also in the number of species projected to encounter appropriate habitat there (Figure 3b).

**FIGURE 4:**
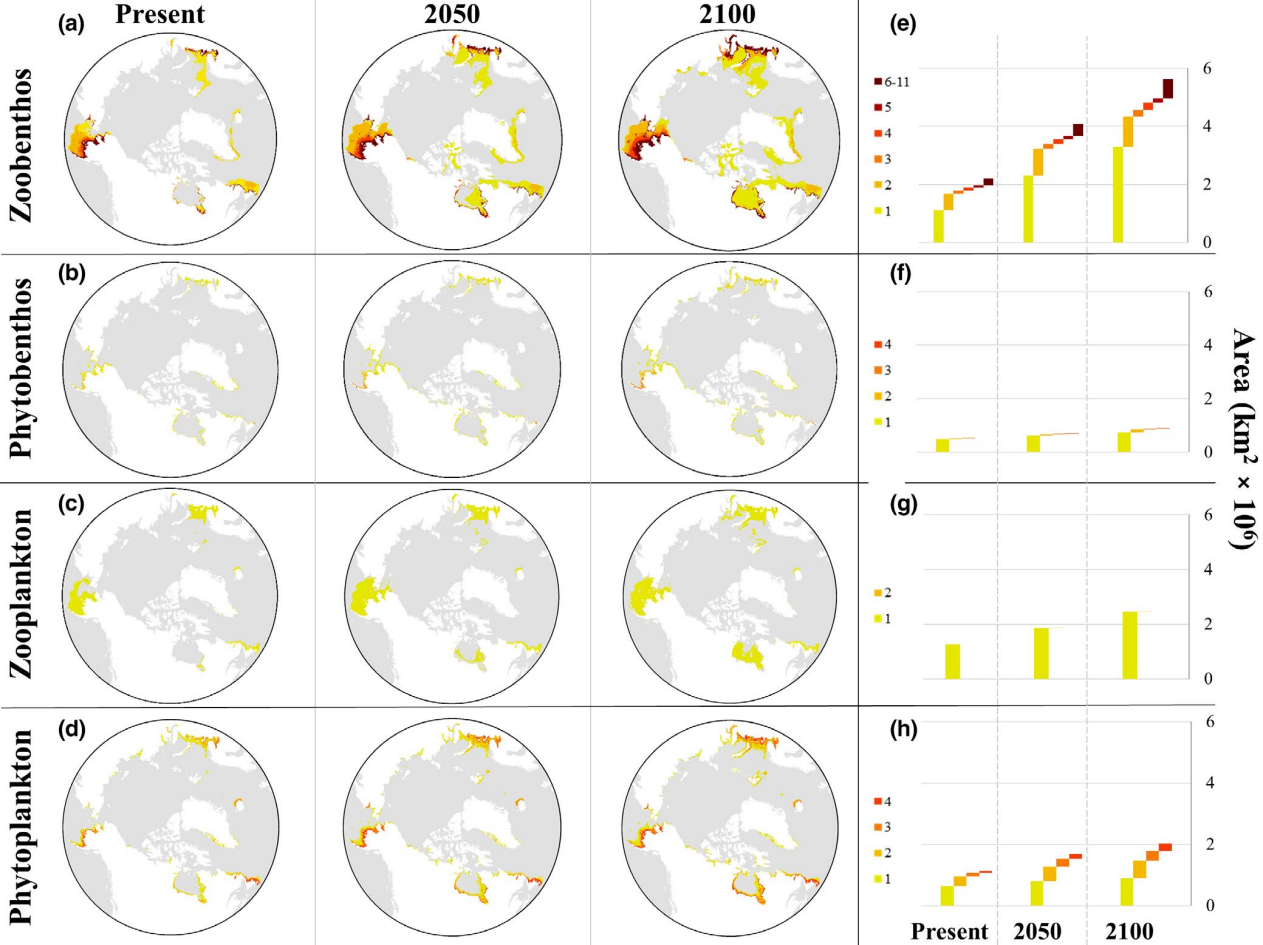
(a–d) Predicted hotspots of aquatic invasive species (AIS) richness per ecological group under present and future (2050 and 2100) conditions at an Arctic scale. Colours represent the number of overlapping species with suitable habitat in a given area. (e–h) Predicted future change of suitable habitat for each category of AIS richness

These same regions were predicted to be hotspots for individual ecological groups, particularly zoobenthos and phytoplankton (Figure 4a,b,d). When evaluated independently, these groups showed the same pattern of future increased areal extent in the predicted suitable habitat, but at varying taxa‐dependent scales (absolute area extent: zoobenthos ~6 × 10^6^ km^2^, phytobenthos, zooplankton and phytoplankton ~1–2 × 10^6^ km^2^; Figure 4a–d). Despite these differences in magnitude for absolute predicted future suitable habitat, at a relative scale the percentage change in suitable habitat was predicted to be greater through time for some groups (e.g. zoobenthos). Furthermore, the relative change in predicted suitable habitat for various categories of AIS richness differed between ecological groups (Figure 4e–h). For example, zoobenthos were predicted to have greater future increases in the areal extent of habitats suitable for a high number of overlapping species (i.e. with high AIS richness).

Only one species of zooplankton (*Aurelia limbata*) was predicted to have extended suitable habitat in the Arctic; hence, no potential hotspots with overlapping species were observed (Figure 4c). It should be highlighted that a few zoobenthic species have native ranges included in the area of analysis: (a) *C. opilio*: Beaufort Sea, Bering Sea and Northern Grandbanks/Labrador; (b) *Littorina littorea*: White Sea; (c) *Mya arenaria*: Northern Grandbanks/Labrador; and (d) *Paralithodes camtschaticus*: Bering Sea (Figure 1; Table S1).

At a global scale, total species richness was higher and largely concentrated on the coasts of Northern Europe and the White Sea, Black Sea, Northwest Atlantic, some regions of the Northwest and Northeast Pacific, Iceland and southern regions of Temperate South America and Temperate Australasia (Figure S2).

### 3.3 Distribution change

**FIGURE 5:**
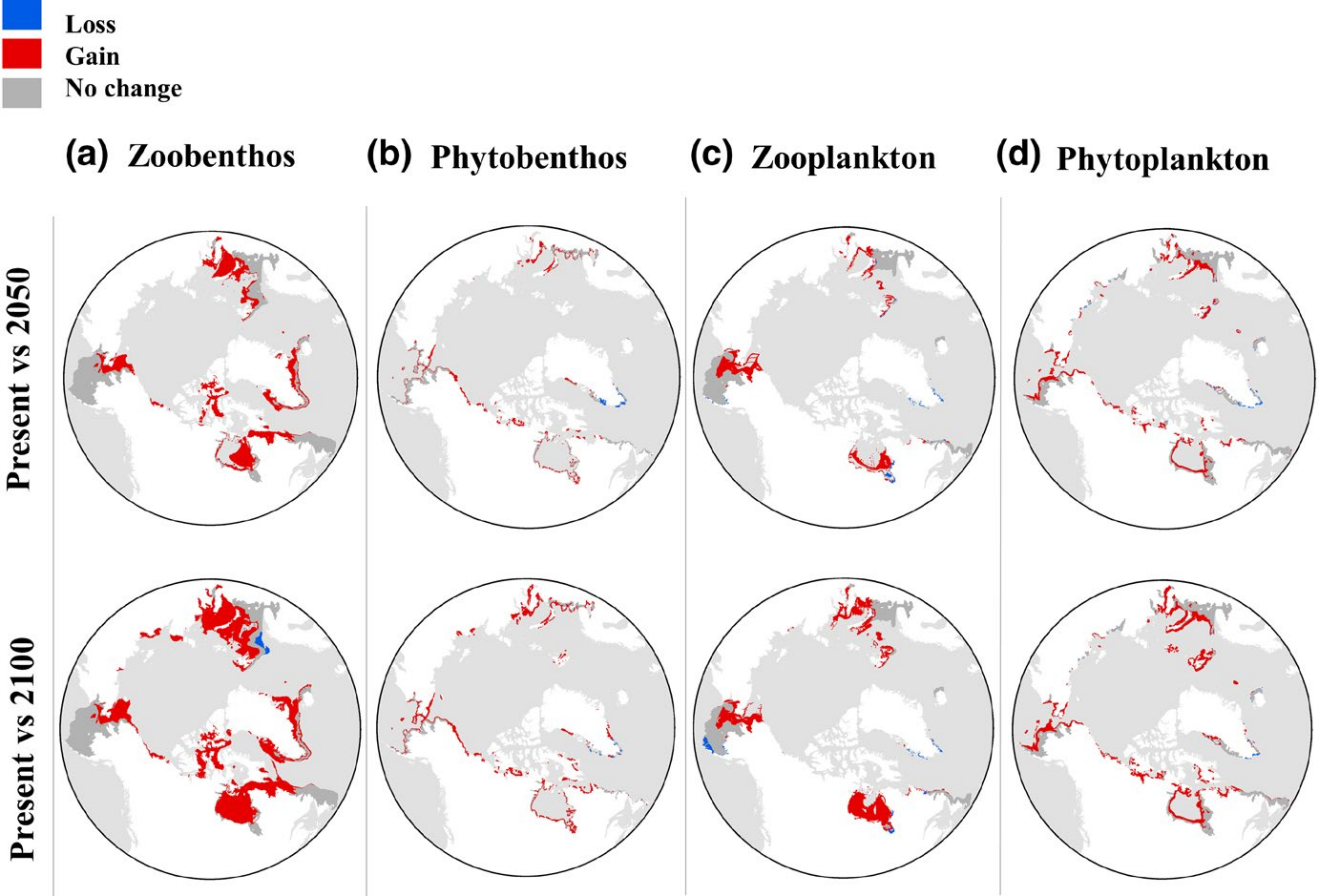
Overall change in predicted suitable habitat for each ecological group at an Arctic scale between present and future scenarios (2050 and 2100): (a) zoobenthos, (b) phytobenthos, (c) zooplankton and (d) phytoplankton

**FIGURE 6:**
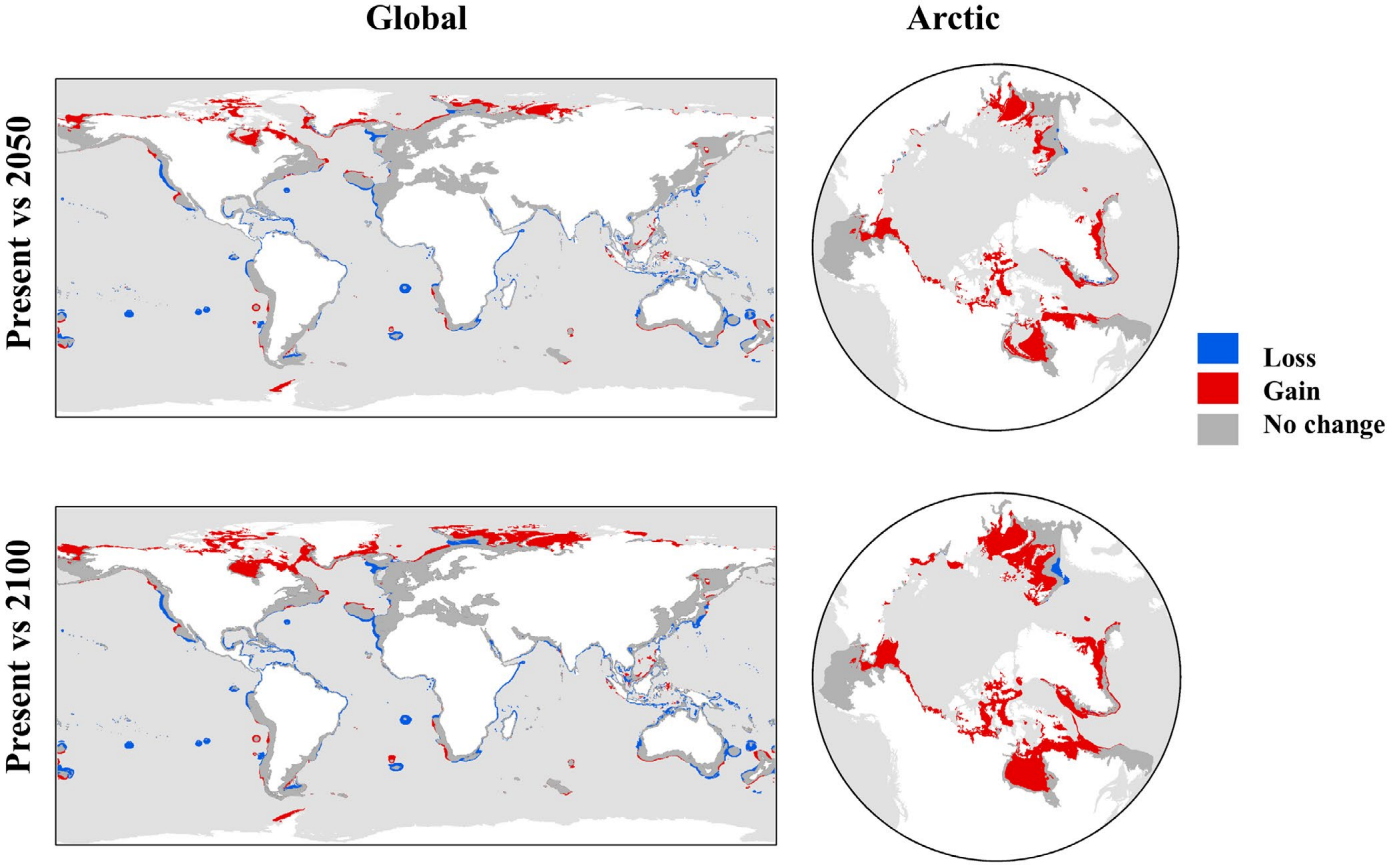
Overall change in predicted suitable habitat of all aquatic invasive species combined at global and pan‐Arctic scales between present and future scenarios (2050 and 2100)

**FIGURE 7:**
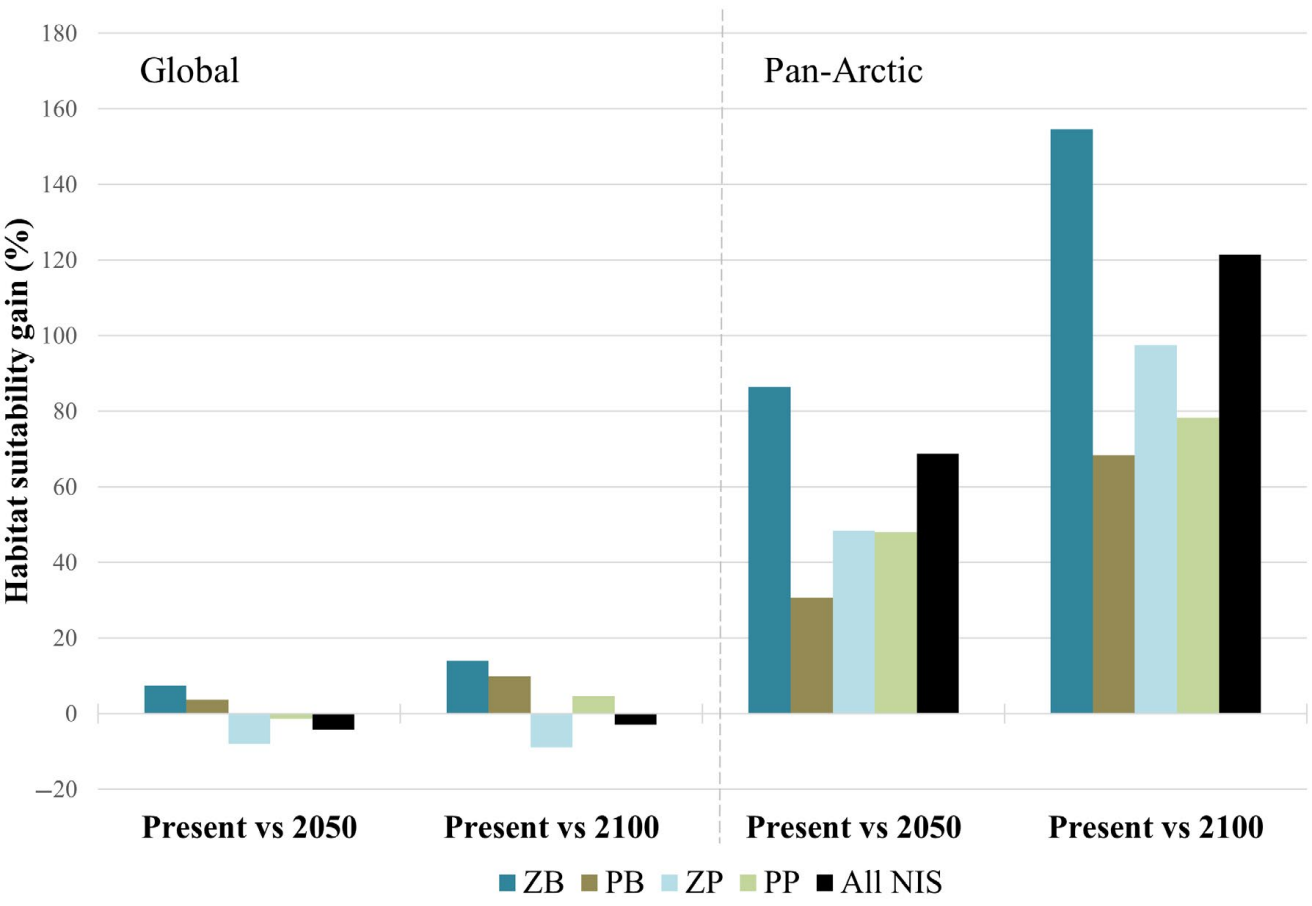
Percentage of predicted habitat suitability change (gain or loss) for the four ecological groups and all aquatic invasive species together at global and pan‐Arctic scales. PB, phytobenthos; PP, phytoplankton; ZB, zoobenthos; ZP, zooplankton

Suitable habitat was projected to increase in the future for all AIS combined as well as for all individual ecological groups in the Arctic realm under both climate change scenarios (Figures 5 and 6), with zoobenthos showing the greatest potential habitat gains (more than double the gains of other ecological groups; Figures 5a and 7) under future climate scenarios. The overall predicted future suitable habitat changes at the pan‐Arctic scale for all AIS combined were net habitat gains of +68.8% and +121.4% by 2050 and 2100, respectively (Figures 6 and 7). However, the same analysis predicted an overall suitable habitat loss at a global scale in both future scenarios (−4.3% by 2050 and −3% by 2100; Figures 6 and 7).

These results show that most modelled species are predicted to find suitable habitats in colder regions, with a trend towards poleward shifts in future distributions, particularly in northern latitudes (the poleward shift to the south is predicted to be lower; Figure 6). At a global scale, however, planktonic species are predicted to experience habitat loss (except for a small habitat gain predicted for phytoplankton towards the year 2100), while benthic species are predicted to have a positive gain, although much less than that at the pan‐Arctic scale (Figure 7; Figure S3).

## 4 DISCUSSION

This study predicted the distribution of suitable habitat for 23 high‐risk AIS at pan‐Arctic and global scales and identified hotspots of suitable habitats for multiple species. Overall, results indicate that suitability will increase over time in Arctic regions, particularly in Hudson Bay, Northern Grand Banks/Labrador, Chukchi/Eastern Bering seas and Barents/White seas regions. Gradually, these regions could become more vulnerable to invasions due to increases in the extent of suitable habitat and in the number of species that gain access to these habitats and establish reproducing populations (i.e. AIS richness). This trend was observed across all assessed ecological groups that showed gains in suitable habitat under future scenarios, with zoobenthos exhibiting the greatest future distributional changes (more than double). Nevertheless, all other ecological groups are predicted to increase their future suitable habitat by 30% up to more than 90%.

Interestingly, predicted shifts under future climate scenarios differed at pan‐Arctic and global scales. When all species were considered collectively, there was a great predicted increase in suitable habitat at the pan‐Arctic scale. In contrast, the net result at a global scale was a predicted loss of suitable habitat, suggesting a marked northward shift with global change. Projected future scenarios considered only the direct effect of climate change. However, projected increases of shipping to the area will likely exacerbate the risk as new transport routes open due to longer ice‐free conditions that increase opportunities for natural resource extraction (Melia et al., 2016; Miller & Ruiz, 2014; Smith & Stephenson, 2013). Likewise, the Antarctic could be affected by native and invasive species from northern regions, given that environmental conditions are similar. Arctic ports could act as possible sources of AIS to the Antarctic since some ships transit between poles and could act as a potential pathway of transportation, although survival and establishment in such scenarios are unknown (McCarthy et al., 2019).

The use of CMIST as a screening tool to identify high‐risk species for specific ecoregions is a recent approach to stay ahead of the invasion process (Drolet et al., 2016; Moore et al., 2018; Therriault et al., 2018). Although the use of CMIST combined with habitat modelling, as done in this study, places more emphasis on the likelihood of invasion than the likelihood of impact component of risk, such a tool may be used as a first step to develop a ranked watch list to guide early detection and monitoring efforts and to prioritize species for detailed risk assessments and potential regulations (Locke, Mandrak, & Therriault, 2011). CMIST provided the opportunity to identify potential AIS of greater risk to the Arctic by considering known species' characteristics and impacts in other regions of the world where they have already invaded. Eighty‐seven per cent of the species included in the present modelling assessment belong to groups with the greatest numbers of known invasions in the marine Arctic ecosystem (Chan et al., 2019). The uneven emphasis of organisms retained for modelling from the different ecological groups (maximum of 11 zoobenthic species versus a minimum of 3 zooplankton species) may, in part, be explained by the fact that most known introduced marine organisms are benthic species (Streftaris, Zenetos, & Papathanassiou, 2005). These proportions are comparable with previous assessment studies (Leidenberger, Obst, et al., 2015), although the uneven coverage does make general trends more difficult to interpret.

Sea surface temperature and land distance were retained in all models, consistent with other studies that found these predictors to contribute significantly to explaining species distributions (Bradie & Leung, 2017; Leidenberger, Obst, et al., 2015; Stelzer, Heyer, Bourlat, & Obst, 2013). Water temperature has been identified as the most relevant predictor of global marine species distribution and land distance of moderate importance for both benthic and planktonic species (Bosch, Tyberghein, Deneudt, Hernandez, & De Clerck, 2018; Bradie & Leung, 2017). Depth was an important predictor, as shown by Bosch et al. (2018) and Snickars et al. (2014) who suggested that bathymetry is of high relevance for modelling various taxa. Ice thickness was moderately important for phytobenthos and zooplankton, perhaps reflecting limits on distribution of the former due to ice abrasion, changes in light exposure and a preference of ice‐free waters for the latter (Clark et al., 2013; Kube, Postel, Honnef, & Augustin, 2007; Pascual et al., 2015; Richardson, 1979). Iron was an important predictor for all modelled phytoplankton species, likely as it is known to be important to phytoplankton growth, abundance, dominance and species distributions (Hecky & Kilham, 1988; Moore et al., 2013) and plays a role in the development of harmful algal bloom species (Doucette & Harrison, 1990; Wells, Mayer, & Guillard, 1991). In Arctic regions, meltwater can be a significant bioavailable source of iron to surrounding coastal oceans (Bhatia et al., 2013; Tovar‐Sánchez et al., 2010) and evidence suggests a link between ice and iron from glacial meltwater leading to blooms in some phytoplankton taxa (Aguilar‐Islas, Rember, Mordy, & Wu, 2008; Joli et al., 2018). This is important given that increased iron due to a shift from an ice‐covered to an open water Arctic Ocean (Screen, 2018; Seneviratne et al., 2018) could create favourable conditions for harmful species that may arrive in the region.

Four Arctic regions (i.e. Hudson Bay, Northern Grand Banks/Labrador, Chukchi/Eastern Bering seas and Barents/White seas) were identified as potential hotspots for invasions. Invasion hotspots could pose even greater risks if they coincide with major shipping routes, biodiversity hotspots or areas of special interest/importance, which is the case for most of the invasion hotspots predicted in the present study. The predicted invasion hotspots overlap with regions that have been highlighted for being special (with regard to their uniqueness, importance for species life histories, threatened species and/or habitats, biological productivity, diversity, etc.). For example, some Ecologically and Biologically Significant Areas (also known as EBSAs) and Marine Refuges coincide with the predicted invasion hotspots in Northern Grand Banks/Labrador, Chukchi/Eastern Bering seas and Barents/White seas (Kenchington et al., 2011; Speer & Laughlin, 2011; Templeman, 2007; www.dfo‐mpo.gc.ca/oceans/oeabcm‐amcepz/refuges/index‐eng.html), as well as in the southern Hudson Bay, which has been identified as being an area of high biological importance (Stephenson & Hartwig, 2010). Ecoregions with a high concentration of polynyas could also be at greater risk, given that they may act as important biological hotspots due to their increased productivity and biodiversity (Marchese, 2015). This may be the case for the Chukchi and Eastern Bering seas, which are known to have concentrations of polynyas (CAFF, 2013). This is consistent with previous studies that have identified biodiversity hotspots in marine and terrestrial ecosystems as being at particular risk to future invasions (Bellard et al., 2014; Li, Liu, Kraus, Tingley, & Li, 2016) and climate change (Ramírez, Afán, Davis, & Chiaradia, 2017). The presence of AIS in biological hotspots could endanger native and endemic species as AIS are considered to be one of the leading causes of biodiversity loss and contemporary extinctions (Bellard, Cassey, et al., 2016; Blackburn et al., 2019) with impacts on ecosystem structure and functioning, including changes in food webs, biomass, flux rates, etc. (Ehrenfeld, 2010). Relating where invasion hotspots overlap with biological hotspots could be crucial for prioritizing conservation efforts.

The Barents Sea has been highlighted as a region that already has a substantial number of invasions and environmental conditions that increase the probability of successful establishment (Chan et al., 2019). Two of the species modelled in the present study, *C. opilio* and *P. camtschaticus*, are already established there (Chan et al., 2019; Dvoretsky & Dvoretsky, 2009; Hansen, 2015). The former is thought to have been introduced by ballast water, while the latter was an intentional introduction (Alvsvåg, Agnalt, & Jørstad, 2009; Jørgensen & Nilssen, 2011). The Barents Sea appears to be in transition from a cold Arctic to warm Atlantic climate regime (Lind, Ingvaldsen, & Furevik, 2018), making it particularly vulnerable to invasion. Indeed, it is predicted to suffer one of the largest future habitat losses by endemic species in the Arctic (Renaud et al., 2019), leaving potential niches available for novel species to occupy. Additionally, Arctic ecoregions will be more exposed to potential future arrivals with further ice reduction and increased navigability of the Northern Sea Route and the Northwest Passage, although much greater investment in infrastructure, navigation and communications would be needed to this end (Buixadé Farré et al., 2014). Nonetheless, both human activities and AIS are likely to increase over time in the Arctic, as has been observed over the last few years (Chan et al., 2019; Dawson, Pizzolato, Howell, Copland, & Johnston, 2018).

At a global scale, planktonic organisms generally showed loss of suitable habitat that, in all but one case, exceeded predicted gains. This may be due to their being dispersive pelagic organisms which have the capability of expanding rapidly and show extensive distribution changes in response to temperature increases related to global warming (Poloczanska et al., 2013). Predicted future changes in sea surface temperatures, in particular, which are expected to outpace shifts in bottom temperature (Levitus et al., 2012), may be driving this pattern. Indeed, models in the present study showed that sea surface temperature was more important for planktonic organisms, whereas bottom temperature was important for benthic organisms. However, the pattern of predicted change by taxa is different at the pan‐Arctic scale where all ecological groups showed high positive suitable habitat gains coinciding with other modelling studies (de Rivera et al., 2011; Goldsmit et al., 2018; Jueterbock et al., 2013; Townhill et al., 2018; Villarino et al., 2015; Ware et al., 2016). This could be explained by the fact that temperature increases have been shown to be greater in the Arctic than in other areas (Bush & Lemmen, 2019; Pendleton et al., 2019) and thus predicted gains in suitable habitat there would be expected to be relatively greater than losses elsewhere.

A marked poleward shift was predicted, consistent with other forecasting studies for various types of marine organisms, including invertebrates, algae and fish (Byrne et al., 2016; Chust et al., 2013; Goldsmit et al., 2018; Jones & Cheung, 2015; Mackey et al., 2012; Townhill et al., 2018; Valle et al., 2014; Wisz et al., 2015). Poleward shifts may serve as an early warning signal of ecosystem change due to climate warming. Multiple studies across different taxa are showing consistency between observed responses of marine organisms to climate change, particularly in high‐latitude regions (Poloczanska et al., 2013). Cold‐adapted species typically have narrow thermal windows and low‐energy demand lifestyles, making them more sensitive to temperature changes (Poloczanska et al., 2016). Predicted poleward shifts in suitable habitat in the present study are disproportionately located in Arctic areas, as has been shown in Cheung et al. (2009), García Molinos et al. (2015) and Jones and Cheung (2015). Invasive species in the Antarctic region have been limited by physiology at cold temperatures rather than geographic limits, but global warming may remove those physiological barriers and alter distributions (Aronson et al., 2007). However, it should be noted that a substantial number of the species modelled here are already distributed in northern/cold regions, which may bias observed patterns. Although, three modelled species (*C. intestinalis*, *B. schlosseri* and *U. pinnatifida*) are among the most likely to become invasive in the Antarctic Peninsula according to a recent horizon scanning study (Hughes et al., 2020).

The locations with greater reductions of the sea ice season over the last 40 years (Stammerjohn, Massom, Rind, & Martinson, 2012) coincide with the regions of predicted invasion hotspots in the present study, suggesting that these areas are already experiencing changing environmental conditions and that such variations are projected to continue in time, including the probability of a complete ice‐free Arctic during summer (Screen, 2018; Sigmond et al., 2018). The combination of species invasions and predicted reductions in sea‐ice cover could alter reproduction/phenology timing, energy pathways and food‐web dynamics with subsequent impacts on production at higher trophic levels (Haug et al., 2017; Poloczanska et al., 2013 and references therein). In this context, AIS could take advantage of new habitats and resources and outcompete native species, which are generally expected to be more sensitive to temperature changes given that they live within a narrow low‐temperature range (Peck, 2005). Invasive, non‐native and native boreal species could thus expand their ranges as suitable habitats in polar regions become more common (de Rivera et al., 2011; Goldsmit et al., 2018; Renaud et al., 2019; Ware et al., 2016) with subsequent impacts on richness, community composition and Arctic ecosystems. However, it remains unknown whether biogeographic boundary locations will change (Costello et al., 2017).

The combination of methods used in this study permitted the inclusion of different modelling algorithms for more robust prediction results based on an ensemble model. Models had varying data requirements (e.g. presence‐background or presence–absence) and used varying statistical approaches (e.g. statistical regression models and machine‐learning decision trees). Modelling studies have restrictions and limits that should be recognized when interpreting results. A main limitation of the present study is that potential species occurrences and range changes are based solely on abiotic factors and do not reflect the realized distribution of a species, assuming that species are in equilibrium with their environment (Bellard et al., 2018; Guisan & Zimmermann, 2000). Biotic interactions, potential resources and life history traits were not included in the modelling exercise, even though they are known to be important in shaping species spatial distributions (Wisz et al., 2013). Moreover, including physiological data such as reproductive temperature to represent phenology, can significantly change SDM predictions. Including this type of data has been shown to strongly limit predicted northward shifts under future climate change scenarios (Chefaoui, Serebryakova, Engelen, Viard, & Serrão, 2019). However, this particular aspect was somewhat offset by the fact that environmental conditions for reproduction, together with conditions needed for different life stages, were considered in this study during the species selection using CMIST scores. Given that the present study modelled the potential distribution of suitable habitat in a new environment, biotic interactions may not be realistic if applied in a different context, such as different regions or time periods (Anderson, 2017 and references therein). Despite limitations and restrictions, SDM can provide valuable information to help manage resources in marine ecosystems that will face increasing anthropogenic pressures (Reiss et al., 2014) and has been shown to be a useful predictive tool to assess various taxa concurrently (Gallardo et al., 2015; Leidenberger, Obst, et al., 2015). SDM may also be particularly useful for regions such as the Arctic, where predicting biodiversity changes under global warming effects is challenging due to the paucity of baseline data for most organisms (Renaud et al., 2019; Wassmann, Duarte, Agusti, & Sejr, 2011).

Distributional change of AIS may increase risk in previously unimpacted areas, potentially creating new problems for wildlife and even human health (such as harmful algal blooms). These types of episodes are being facilitated by climate change (Hallegraeff, 2010; Kordas, Harley, & O'Connor, 2011; Poloczanska et al., 2013, 2016). The present study predicts changes over such an extensive area (~6 × 10^6^ km^2^, equivalent to almost three times the size of Greenland) that altered community structure may be widespread. Results presented here provide information on AIS that pose some of the greatest threats to the Arctic, with areas at greatest risk identified as hotspots. This information is valuable given that aquatic invaders are understudied in the context of climate change (Bellard et al., 2018). The Arctic is predicted to be affected by increased habitat suitability for a number of potential AIS and, given its vast area, could be severely impacted by AIS accumulating in specific locations under both current and future environmental conditions. Identification of hotspots through SDM, predictions of habitat vulnerability and particular areas of concern could guide ballast water practices and other management actions, including prevention, early detection monitoring, rapid response and conservation planning. Information such as that provided by the present study should help guide how to prioritize management efforts in this unique and vast region.

## Supporting information

Fig S1

Fig S2

Fig S3

Table S1

Table S2

Table S3

Table S4

Table S5

Table S5

## Data Availability

Data sharing is not applicable to this article as no new data were created in this study. The data that support the findings of this study were derived from the following resources available in the public domain: OBIS (https://obis.org/), GBIF (https://www.gbif.org/fr/), Bio‐ORACLE (https://www.bio‐oracle.org/) and AquaMaps (https://www.aquamaps.org/).

## References

[gcb15159-bib-0001] Aguilar‐Islas, A. M. , Rember, R. D. , Mordy, C. W. , & Wu, J. (2008). Sea ice‐derived dissolved iron and its potential influence on the spring algal bloom in the Bering Sea. Geophysical Research Letters, 35(24). 10.1029/2008GL035736

[gcb15159-bib-0002] Allouche, O. , Tsoar, A. , & Kadmon, R. (2006). Assessing the accuracy of species distribution models: Prevalence, kappa and the true skill statistic (TSS). Journal of Applied Ecology, 43(6), 1223–1232. 10.1111/j.1365-2664.2006.01214.x

[gcb15159-bib-0003] Alvsvåg, J. , Agnalt, A. L. , & Jørstad, K. E. (2009). Evidence for a permanent establishment of the snow crab (*Chionoecetes opilio*) in the Barents Sea. Biological Invasions, 11(3), 587–595. 10.1007/s10530-008-9273-7

[gcb15159-bib-0004] Anderson, R. P. (2017). When and how should biotic interactions be considered in models of species niches and distributions? Journal of Biogeography, 44(1), 8–17. 10.1111/jbi.12825

[gcb15159-bib-0005] Andrade, A. F. A. , Velazco, S. J. E. , & Júnior, P. D. M. (2020). ENMTML: An R package for a straightforward construction of complex ecological niche models. Environmental Modelling & Software, 125, 104615 10.1016/j.envsoft.2019.104615

[gcb15159-bib-0006] Araújo, M. B. , & New, M. (2007). Ensemble forecasting of species distributions. Trends in Ecology & Evolution, 22, 42–47. 10.1016/j.tree.2006.09.010 17011070

[gcb15159-bib-0007] Araújo, M. B. , Pearson, R. G. , Thuiller, W. , & Erhard, M. (2005). Validation of species–climate impact models under climate change. Global Change Biology, 11(9), 1504–1513. 10.1111/j.1365-2486.2005.01000.x

[gcb15159-bib-0008] Archambault, P. , Snelgrove, P. V. R. , Fisher, J. A. D. , Gagnon, J.‐M. , Garbary, D. J. , Harvey, M. , … Poulin, M. (2010). From sea to sea: Canada's three oceans of biodiversity. PLoS ONE, 5(8), e12182 10.1371/journal.pone.0012182 20824204 PMC2930843

[gcb15159-bib-0009] Aronson, R. B. , Thatje, S. , Clarke, A. , Peck, L. S. , Blake, D. B. , Wilga, C. D. , & Seibel, B. A. (2007). Climate change and invasibility of the Antarctic benthos. Annual Review of Ecology, Evolution and Systematics, 38, 129–154. 10.1146/annurev.ecolsys.38.091206.095525

[gcb15159-bib-0010] Ashton, G. V. , Riedlecker, E. I. , & Ruiz, G. M. (2008). First non‐native crustacean established in coastal waters of Alaska. Aquatic Biology, 3(2), 133–137. 10.3354/ab00070

[gcb15159-bib-0011] Assis, J. , Tyberghein, L. , Bosch, S. , Verbruggen, H. , Serrão, E. A. , & De Clerck, O. (2018). Bio‐ORACLE v2. 0: Extending marine data layers for bioclimatic modelling. Global Ecology and Biogeography, 27(3), 277–284. 10.1111/geb.12693

[gcb15159-bib-0012] Bailey, S. A. (2015). An overview of thirty years of research on ballast water as a vector for aquatic invasive species to freshwater and marine environments. Aquatic Ecosystem Health & Management, 18(3), 261–268. 10.1080/14634988.2015.1027129

[gcb15159-bib-0013] Barbosa, F. G. , & Schneck, F. (2015). Characteristics of the top‐cited papers in species distribution predictive models. Ecological Modelling, 313, 77–83. 10.1016/j.ecolmodel.2015.06.014

[gcb15159-bib-0014] Barbosa, F. G. , Schneck, F. , & Melo, A. S. (2012). Use of ecological niche models to predict the distribution of invasive species: A scientometric analysis. Brazilian Journal of Biology, 72(4), 821–829. 10.1590/S1519-69842012000500007 23295510

[gcb15159-bib-0015] Barnes, D. K. A. (1999). The influence of ice on polar nearshore benthos. Journal of the Marine Biological Association of the United Kingdom, 79(3), 401–407. 10.1017/S0025315498000514

[gcb15159-bib-0016] Barnhart, K. R. , Miller, C. R. , Overeem, I. , & Kay, J. E. (2016). Mapping the future expansion of Arctic open water. Nature Climate Change, 6(3), 280–285. 10.1038/nclimate2848

[gcb15159-bib-0017] Battini, N. , Farías, N. , Giachetti, C. B. , Schwindt, E. , & Bortolus, A. (2019). Staying ahead of invaders: Using species distribution modeling to predict alien species' potential niche shifts. Marine Ecology Progress Series, 612, 127–140. 10.3354/meps12878

[gcb15159-bib-0018] Belanger, C. L. , Jablonski, D. , Roy, K. , Berke, S. K. , Krug, A. Z. , & Valentine, J. W. (2012). Global environmental predictors of benthic marine biogeographic structure. Proceedings of the National Academy of Sciences of the United States of America, 109(35), 14046–14051. 10.1073/pnas.1212381109 22904189 PMC3435205

[gcb15159-bib-0019] Bellard, C. , Cassey, P. , & Blackburn, T. M. (2016). Alien species as a driver of recent extinctions. Biology Letters, 12(2), 20150623 10.1098/rsbl.2015.0623 26888913 PMC4780541

[gcb15159-bib-0020] Bellard, C. , Jeschke, J. M. , Leroy, B. , & Mace, G. M. (2018). Insights from modeling studies on how climate change affects invasive alien species geography. Ecology and Evolution, 8(11), 5688–5700. 10.1002/ece3.4098 29938085 PMC6010883

[gcb15159-bib-0021] Bellard, C. , Leclerc, C. , Leroy, B. , Bakkenes, M. , Veloz, S. , Thuiller, W. , & Courchamp, F. (2014). Vulnerability of biodiversity hotspots to global change. Global Ecology and Biogeography, 23(12), 1376–1386. 10.1111/geb.12228

[gcb15159-bib-0022] Bellard, C. , Leroy, B. , Thuiller, W. , Rysman, J. F. , & Courchamp, F. (2016). Major drivers of invasion risks throughout the world. Ecosphere, 7(3), e01241 10.1002/ecs2.1241

[gcb15159-bib-0023] Bellard, C. , Thuiller, W. , Leroy, B. , Genovesi, P. , Bakkenes, M. , & Courchamp, F. (2013). Will climate change promote future invasions? Global Change Biology, 19(12), 3740–3748. 10.1111/gcb.12344 23913552 PMC3880863

[gcb15159-bib-0024] Bhatia, M. P. , Kujawinski, E. B. , Das, S. B. , Breier, C. F. , Henderson, P. B. , & Charette, M. A. (2013). Greenland meltwater as a significant and potentially bioavailable source of iron to the ocean. Nature Geoscience, 6(4), 274 10.1038/ngeo1746

[gcb15159-bib-0025] Blackburn, T. M. , Bellard, C. , & Ricciardi, A. (2019). Alien versus native species as drivers of recent extinctions. Frontiers in Ecology and the Environment, 17(4), 203–207. 10.1002/fee.2020

[gcb15159-bib-0026] Bosch, S. , Tyberghein, L. , Deneudt, K. , Hernandez, F. , & De Clerck, O. (2018). In search of relevant predictors for marine species distribution modelling using the MarineSPEED benchmark dataset. Diversity and Distributions, 24(2), 144–157. 10.1111/ddi.12668

[gcb15159-bib-0027] Bradie, J. , & Leung, B. (2017). A quantitative synthesis of the importance of variables used in MaxEnt species distribution models. Journal of Biogeography, 44(6), 1344–1361. 10.1111/jbi.12894

[gcb15159-bib-0028] Broennimann, O. , & Guisan, A. (2008). Predicting current and future biological invasions: Both native and invaded ranges matter. Biology Letters, 4(5), 585–589. 10.1098/rsbl.2008.0254 18664415 PMC2610080

[gcb15159-bib-0029] Brown, E. A. , Chain, F. J. J. , Zhan, A. , MacIsaac, H. J. , & Cristescu, M. E. (2016). Early detection of aquatic invaders using metabarcoding reveals a high number of non‐indigenous species in Canadian ports. Diversity and Distributions, 22(10), 1045–1059. 10.1111/ddi.12465

[gcb15159-bib-0030] Brown, J. L. (2014). SDMtoolbox: A python‐based GIS toolkit for landscape genetic, biogeographic and species distribution model analyses. Methods in Ecology and Evolution, 5(7), 694–700. 10.1111/2041-210X.12200 PMC5721907 29230356

[gcb15159-bib-0031] Bucklin, D. N. , Basille, M. , Benscoter, A. M. , Brandt, L. A. , Mazzotti, F. J. , Romañach, S. S. , … Watling, J. I. (2015). Comparing species distribution models constructed with different subsets of environmental predictors. Diversity and Distributions, 21(1), 23–35. 10.1111/ddi.12247

[gcb15159-bib-0032] Buixadé Farré, A. , Stephenson, S. R. , Chen, L. , Czub, M. , Dai, Y. , Demchev, D. , … Wighting, J. (2014). Commercial Arctic shipping through the Northeast Passage: Routes, resources, governance, technology, and infrastructure. Polar Geography, 37(4), 298–324. 10.1080/1088937X.2014.965769

[gcb15159-bib-0033] Bush E., & Lemmen D. S. (Eds.). (2019). Canada's changing climate report. Government of Canada. Retrieved from https://changingclimate.ca/CCCR2019/

[gcb15159-bib-0034] Byrne, M. , Gall, M. , Wolfe, K. , & Agüera, A. (2016). From pole to pole: The potential for the Arctic seastar *Asterias amurensis* to invade a warming Southern Ocean. Global Change Biology, 22(12), 3874–3887. 10.1111/gcb.13304 27029504

[gcb15159-bib-0035] CAFF (Conservation of Arctic Flora and Fauna) . (2013). Arctic biodiversity assessment: Status and trends in Arctic biodiversity. Akureyri, Iceland Retrieved from http://arcticlcc.org/assets/resources/ABA2013Science.pdf

[gcb15159-bib-0036] CAFF (Conservation of Arctic Flora and Fauna) and PAME (Protection of the Arctic Marine Envirnoment) . (2017). Arctic invasive alien species: Strategy and action plan. Akureyri, Iceland Retrieved from https://www.caff.is/invasive‐species

[gcb15159-bib-0037] Catford, J. A. , Vesk, P. A. , White, M. D. , & Wintle, B. A. (2011). Hotspots of plant invasion predicted by propagule pressure and ecosystem characteristics. Diversity and Distributions, 17(6), 1099–1110. 10.1111/j.1472-4642.2011.00794.x

[gcb15159-bib-0038] Chain, F. J. J. , Brown, E. A. , MacIsaac, H. J. , & Cristescu, M. E. (2016). Metabarcoding reveals strong spatial structure and temporal turnover of zooplankton communities among marine and freshwater ports. Diversity and Distributions, 22(5), 493–504. 10.1111/ddi.12427

[gcb15159-bib-0039] Chan, F. T. , Bailey, S. , Wiley, C. , & MacIsaac, H. (2013). Relative risk assessment for ballast‐mediated invasions at Canadian Arctic ports. Biological Invasions, 15(2), 295–308. 10.1007/s10530-012-0284-z

[gcb15159-bib-0040] Chan, F. T. , MacIsaac, H. J. , & Bailey, S. A. (2015). Relative importance of vessel hull fouling and ballast water as transport vectors of nonindigenous species to the Canadian Arctic. Canadian Journal of Fisheries and Aquatic Sciences, 72(8), 1230–1242. 10.1139/cjfas-2014-0473

[gcb15159-bib-0041] Chan, F. T. , MacIsaac, H. J. , & Bailey, S. A. (2016). Survival of ship biofouling assemblages during and after voyages to the Canadian Arctic. Marine Biology, 163(12), 250 10.1007/s00227-016-3029-1 27980347 PMC5106487

[gcb15159-bib-0042] Chan, F. T. , Stanislawczyk, K. , Sneekes, A. C. , Dvoretsky, A. , Gollasch, S. , Minchin, D. , … Bailey, S. A. (2019). Climate change opens new frontiers for marine species in the Arctic: Current trends and future invasion risks. Global Change Biology, 25(1), 25–38. 10.1111/gcb.14469 30295388 PMC7379606

[gcb15159-bib-0043] Chefaoui, R. M. , Serebryakova, A. , Engelen, A. H. , Viard, F. , & Serrão, E. A. (2019). Integrating reproductive phenology in ecological niche models changed the predicted future ranges of a marine invader. Diversity and Distributions, 25(5), 688–700. 10.1111/ddi.12910

[gcb15159-bib-0044] Cheung, W. W. L. , Lam, V. W. Y. , Sarmiento, J. L. , Kearney, K. , Watson, R. , & Pauly, D. (2009). Projecting global marine biodiversity impacts under climate change scenarios. Fish and Fisheries, 10(3), 235–251. 10.1111/j.1467-2979.2008.00315.x

[gcb15159-bib-0045] Chust, G. , Castellani, C. , Licandro, P. , Ibaibarriaga, L. , Sagarminaga, Y. , & Irigoien, X. (2013). Are *Calanus* spp. shifting poleward in the North Atlantic? A habitat modelling approach. ICES Journal of Marine Science, 71(2), 241–253. 10.1093/icesjms/fst147

[gcb15159-bib-0046] Chust, G. , Villarino, E. , Chenuil, A. , Irigoien, X. , Bizsel, N. , Bode, A. , … Borja, A. (2016). Dispersal similarly shapes both population genetics and community patterns in the marine realm. Scientific Reports, 6, 28730 10.1038/srep28730 27344967 PMC4921837

[gcb15159-bib-0047] Clark, G. F. , Stark, J. S. , Johnston, E. L. , Runcie, J. W. , Goldsworthy, P. M. , Raymond, B. , & Riddle, M. J. (2013). Light‐driven tipping points in polar ecosystems. Global Change Biology, 19(12), 3749–3761. 10.1111/gcb.12337 23893603

[gcb15159-bib-0048] Clarke, L. , Edmonds, J. , Jacoby, H. , Pitcher, H. , Reilly, J. , & Richels, R. (2007). Scenarios of greenhouse gas emissions and atmospheric concentrations. Sub‐Report 2.1a of Synthesis and Assessment Product 2.1 by the U.S. Climate Change Science Program and the Subcommittee on Global Change Research, Department of Energy, Office of Biological & Environmental Research, Washington, DC Retrieved from http://www.climatescience.gov/Library/sap/sap2‐1/finalreport/default.htm

[gcb15159-bib-0049] Cook, E. J. , Payne, R. D. , MacLeod, A. , & Brown, S. F. (2016). Marine biosecurity: Protecting indigenous marine species. Research and Reports in Biodiversity Studies, 5, 1–14. 10.2147/RRBS.S63402

[gcb15159-bib-0050] Costello, M. J. , Basher, Z. , McLeod, L. , Asaad, I. , Claus, S. , Vandepitte, L. , … Appeltans, W. (2017). Methods for the study of marine biodiversity In Walters M. & Scholes R. J. (Eds.), The GEO handbook on biodiversity observation networks (pp. 129–163). 10.1007/978-3-319-27288-7_6

[gcb15159-bib-0051] Cusson, M. , Archambault, P. , & Aitken, A. (2007). Biodiversity of benthic assemblages on the Arctic continental shelf: Historical data from Canada. Marine Ecology Progress Series, 331, 291–304. 10.3354/meps331291

[gcb15159-bib-0052] Davidson, I. C. , Zabin, C. J. , Chang, A. L. , Brown, C. W. , Sytsma, M. D. , & Ruiz, G. M. (2010). Recreational boats as potential vectors of marine organisms at an invasion hotspot. Aquatic Biology, 11(2), 179–191. 10.3354/ab00302

[gcb15159-bib-0053] Dawson, J. , Pizzolato, L. , Howell, S. E. L. , Copland, L. , & Johnston, M. E. (2018). Temporal and spatial patterns of ship traffic in the Canadian Arctic from 1990 to 2015. Arctic, 71(1), 15–26. 10.14430/arctic4698

[gcb15159-bib-0054] de Rivera, C. E. , Steves, B. P. , Fofonoff, P. W. , Hines, A. H. , & Ruiz, G. M. (2011). Potential for high‐latitude marine invasions along western North America. Diversity and Distributions, 17(6), 1198–1209. 10.1111/j.1472-4642.2011.00790.x

[gcb15159-bib-0055] DFO . (2017). Screening of the Pacific Fishery Regulations (PFR) Schedule VIII Species for Risk of Invasiveness. DFO Canadian Science Advisory Secretariat Science Response (2017/040). Retrieved from http://publications.gc.ca/site/eng/9.847598/publication.html

[gcb15159-bib-0056] Dispas, A. (2019). Étude de référence sur la biodiversité du mésozooplancton dans quatre ports de l'Arctique canadien en vue d'une augmentation de l'activité maritime, de l'exploitation des ressources et du réchauffement climatique. Master's thesis. Retrieved from http://semaphore.uqar.ca/id/eprint/1481/

[gcb15159-bib-0057] Dormann, C. F. , Elith, J. , Bacher, S. , Buchmann, C. , Carl, G. , Carré, G. , … Lautenbach, S. (2013). Collinearity: A review of methods to deal with it and a simulation study evaluating their performance. Ecography, 36(1), 27–46. 10.1111/j.1600-0587.2012.07348.x

[gcb15159-bib-0058] Doucette, G. J. , & Harrison, P. J. (1990). Some effects of iron and nitrogen stress on the red tide dinoflagellate *Gymnodinium sanguineum* . Marine Ecology Progress Series, 62(3), 293–306. 10.3354/meps062293

[gcb15159-bib-0059] Drake, J. M. , & Lodge, D. M. (2004). Global hot spots of biological invasions: Evaluating options for ballast–water management. Proceedings of the Royal Society of London B: Biological Sciences, 271(1539), 575–580. 10.1098/rspb.2003.2629 PMC1691629 15156914

[gcb15159-bib-0060] Drake, J. M. , & Lodge, D. M. (2007). Hull fouling is a risk factor for intercontinental species exchange in aquatic ecosystems. Aquatic Invasions, 2(2), 121–131. 10.3391/ai.2007.2.2.7

[gcb15159-bib-0061] Drolet, D. , DiBacco, C. , Locke, A. , McKenzie, C. H. , McKindsey, C. W. , Moore, A. M. , … Therriault, T. W. (2016). Evaluation of a new screening‐level risk assessment tool applied to non‐indigenous marine invertebrates in Canadian coastal waters. Biological Invasions, 18(1), 279–294. 10.1007/s10530-015-1008-y

[gcb15159-bib-0062] Duffy, G. A. , Coetzee, B. W. , Latombe, G. , Akerman, A. H. , McGeoch, M. A. , & Chown, S. L. (2017). Barriers to globally invasive species are weakening across the Antarctic. Diversity and Distributions, 23(9), 982–996. 10.1111/ddi.12593

[gcb15159-bib-0063] Dvoretsky, A. G. , & Dvoretsky, V. G. (2009). Fouling community of the red king crab, *Paralithodes camtschaticus* (Tilesius 1815), in a subarctic fjord of the Barents Sea. Polar Biology, 32(7), 1047–1054. 10.1007/s00300-009-0604-7

[gcb15159-bib-0064] Early, R. , Bradley, B. A. , Dukes, J. S. , Lawler, J. J. , Olden, J. D. , Blumenthal, D. M. , … Tatem, A. J. (2016). Global threats from invasive alien species in the twenty‐first century and national response capacities. Nature Communications, 7, 12485 10.1038/ncomms12485 PMC4996970 27549569

[gcb15159-bib-0065] Edelist, D. , Rilov, G. , Golani, D. , Carlton, J. T. , & Spanier, E. (2013). Restructuring the Sea: Profound shifts in the world's most invaded marine ecosystem. Diversity and Distributions, 19(1), 69–77. 10.1111/ddi.12002

[gcb15159-bib-0066] Ehrenfeld, J. G. (2010). Ecosystem consequences of biological invasions. Annual Review of Ecology, Evolution and Systematics, 41, 59–80. 10.1146/annurev-ecolsys-102209-144650

[gcb15159-bib-0067] Elith, J. , H. Graham, C. , P. Anderson, R. , Dudík, M. , Ferrier, S. , Guisan, A. , … Zimmermann, N. E. (2006). Novel methods improve prediction of species' distributions from occurrence data. Ecography, 29(2), 129–151. 10.1111/j.2006.0906-7590.04596.x

[gcb15159-bib-0068] Elith, J. , Phillips, S. J. , Hastie, T. , Dudík, M. , Chee, Y. E. , & Yates, C. J. (2011). A statistical explanation of MaxEnt for ecologists. Diversity and Distributions, 17(1), 43–57. 10.1111/j.1472-4642.2010.00725.x

[gcb15159-bib-0069] Floerl, O. (2014). Management challenges and opportunities for marine biosecurity in the Arctic In Fernandez L., Brooks A. K., & Vestergaard N. (Eds.), Marine invasive species in the Arctic (pp. 57–67). Copenhagen, Denmark: TemaNord.

[gcb15159-bib-0070] Gallardo, B. , Zieritz, A. , & Aldridge, D. C. (2015). The importance of the human footprint in shaping the global distribution of terrestrial, freshwater and marine invaders. PLoS ONE, 10(5), e0125801 10.1371/journal.pone.0125801 26018575 PMC4446263

[gcb15159-bib-0071] García Molinos, J. , Halpern, B. S. , Schoeman, D. S. , Brown, C. J. , Kiessling, W. , Moore, P. J. , … Burrows, M. T. (2015). Climate velocity and the future global redistribution of marine biodiversity. Nature Climate Change, 6, 83 10.1038/nclimate2769

[gcb15159-bib-0072] García‐Roselló, E. , Guisande, C. , Manjarrés‐Hernández, A. , González‐Dacosta, J. , Heine, J. , Pelayo‐Villamil, P. , … Lobo, J. M. (2015). Can we derive macroecological patterns from primary Global Biodiversity Information Facility data? Global Ecology and Biogeography, 24(3), 335–347. 10.1111/geb.12260

[gcb15159-bib-0073] Gíslason, Ó. S. , Halldórsson, H. P. , Pálsson, M. F. , Pálsson, S. , Davíðsdóttir, B. , & Svavarsson, J. (2014). Invasion of the Atlantic rock crab (*Cancer irroratus*) at high latitudes. Biological Invasions, 16(9), 1865–1877. 10.1007/s10530-013-0632-7

[gcb15159-bib-0074] Golder (Golder Associates Ltd.) . (2018). 2017 marine environmental effects monitoring program (MEEMP) and aquatic invasive species (AIS) monitoring program. (Report No. 1663724–048‐R‐Rev0). Mary River Project; submitted to Baffinland Iron Mines Corporation, Oakville, ON Retrieved from http://www.baffinland.com/document‐portal‐new/?cat=4&archive=1&lang=en

[gcb15159-bib-0075] Goldsmit, J. , Archambault, P. , Chust, G. , Villarino, E. , Liu, G. , Lukovich, J. V. , … Howland, K. L. (2018). Projecting present and future habitat suitability of ship‐mediated aquatic invasive species in the Canadian Arctic. Biological Invasions, 20(2), 501–517. 10.1007/s10530-017-1553-7

[gcb15159-bib-0076] Goldsmit, J. , Howland, K. L. , & Archambault, P. (2014). Establishing a baseline for early detection of non‐indigenous species in ports of the Canadian Arctic. Aquatic Invasions, 9(3), 327–342. 10.3391/ai.2014.9.3.08

[gcb15159-bib-0077] Goldsmit, J. , McKindsey, C. , Archambault, P. , & Howland, K. L. (2019). Ecological risk assessment of predicted marine invasions in the Canadian Arctic. PLoS ONE, 14(2), e0211815 10.1371/journal.pone.0211815 30730941 PMC6366784

[gcb15159-bib-0078] Goldsmit, J. , Nudds, S. H. , Stewart, D. B. , Higdon, J. W. , Hannah, C. G. , & Howland, K. L. (2019). Where else? Assessing zones of alternate ballast water exchange in the Canadian eastern Arctic. Marine Pollution Bulletin, 139, 74–90. 10.1016/j.marpolbul.2018.11.062 30686452

[gcb15159-bib-0079] Guisan, A. , Graham, C. H. , Elith, J. , & Huettmann, F. (2007). Sensitivity of predictive species distribution models to change in grain size. Diversity and Distributions, 13(3), 332–340. 10.1111/j.1472-4642.2007.00342.x

[gcb15159-bib-0080] Guisan, A. , & Zimmermann, N. E. (2000). Predictive habitat distribution models in ecology. Ecological Modelling, 135(2), 147–186. 10.1016/S0304-3800(00)00354-9

[gcb15159-bib-0081] Guisan, A. , Zimmermann, N. E. , Elith, J. , Graham, C. H. , Phillips, S. , & Peterson, A. T. (2007). What matters for predicting the occurrences of trees: Techniques, data, or species' characteristics? Ecological Monographs, 77(4), 615–630. 10.1890/06-1060.1

[gcb15159-bib-0082] Hallegraeff, G. M. (2010). Ocean climate change, phytoplankton community responses, and harmful algal blooms: A formidable predictive challenge. Journal of Phycology, 46(2), 220–235. 10.1111/j.1529-8817.2010.00815.x

[gcb15159-bib-0083] Hansen, H. S. B. (2015). Snow crab (*Chionoecetes opilio*) in the Barents Sea. Diet, biology and management. Master's thesis. Retrieved from https://munin.uit.no/handle/10037/7746

[gcb15159-bib-0084] Hao, T. , Elith, J. , Guillera‐Arroita, G. , & Lahoz‐Monfort, J. J. (2019). A review of evidence about use and performance of species distribution modelling ensembles like BIOMOD. Diversity and Distributions, 25(5), 839–852. 10.1111/ddi.12892

[gcb15159-bib-0085] Haug, T. , Bogstad, B. , Chierici, M. , Gjøsæter, H. , Hallfredsson, E. H. , Høines, Å. S. , … Sunnanå, K. (2017). Future harvest of living resources in the Arctic Ocean north of the Nordic and Barents Seas: A review of possibilities and constraints. Fisheries Research, 188, 38–57. 10.1016/j.fishres.2016.12.002

[gcb15159-bib-0086] Hecky, R. E. , & Kilham, P. (1988). Nutrient limitation of phytoplankton in freshwater and marine environments: A review of recent evidence on the effects of enrichment. Limnology and Oceanography, 33(4, part2), 796–822. 10.4319/lo.1988.33.4part2.0796

[gcb15159-bib-0087] Hewitt, C. L. , Campbell, M. L. , & Gollasch, S. (2006). Alien species in aquaculture. Considerations for responsible use. Gland, Switzerland and Cambridge, UK: IUCN Retrieved from https://www.iucn.org/content/alien‐species‐aquaculture‐considerations‐responsible‐use‐0

[gcb15159-bib-0088] Hijmans, R. J. (2012). Cross‐validation of species distribution models: Removing spatial sorting bias and calibration with a null model. Ecology, 93(3), 679–688. 10.1890/11-0826.1 22624221

[gcb15159-bib-0089] Hijmans, R. J. , & Graham, C. H. (2006). The ability of climate envelope models to predict the effect of climate change on species distributions. Global Change Biology, 12(12), 2272–2281. 10.1111/j.1365-2486.2006.01256.x

[gcb15159-bib-0090] Hughes, K. A. , Pescott, O. L. , Peyton, J. , Adriaens, T. , Cottier‐Cook, E. J. , Key, G. , … Roy, H. E. (2020). Invasive non‐native species likely to threaten biodiversity and ecosystems in the Antarctic Peninsula region. Global Change Biology, 26(4), 2702–2716. 10.1111/gcb.14938 PMC7154743 31930639

[gcb15159-bib-0091] Ibanez, I. , Silander Jr., J. A. , Allen, J. M. , Treanor, S. A. , & Wilson, A. (2009). Identifying hotspots for plant invasions and forecasting focal points of further spread. Journal of Applied Ecology, 46(6), 1219–1228. 10.1111/j.1365-2664.2009.01736.x

[gcb15159-bib-0092] Jahn, A. (2018). Reduced probability of ice‐free summers for 1.5° C compared to 2° C warming. Nature Climate Change, 8(5), 409 10.1038/s41558-018-0127-8

[gcb15159-bib-0093] Jensen, L. Ø. , Mousing, E. A. , & Richardson, K. (2017). Using species distribution modelling to predict future distributions of phytoplankton: Case study using species important for the biological pump. Marine Ecology, 38(3), e12427 10.1111/maec.12427

[gcb15159-bib-0094] Jiménez‐Valverde, A. , & Lobo, J. M. (2007). Threshold criteria for conversion of probability of species presence to either–or presence–absence. Acta Oecologica, 31(3), 361–369. 10.1016/j.actao.2007.02.001

[gcb15159-bib-0095] Joli, N. , Gosselin, M. , Ardyna, M. , Babin, M. , Onda, D. F. , Tremblay, J.‐É. , & Lovejoy, C. (2018). Need for focus on microbial species following ice melt and changing freshwater regimes in a Janus Arctic Gateway. Scientific Reports, 8(1), 9405 10.1038/s41598-018-27705-6 29925879 PMC6010473

[gcb15159-bib-0096] Jones, M. C. , & Cheung, W. W. (2015). Multi‐model ensemble projections of climate change effects on global marine biodiversity. ICES Journal of Marine Science, 72(3), 741–752. 10.1093/icesjms/fsu172

[gcb15159-bib-0097] Jørgensen, L. L. , & Nilssen, E. M. (2011). The invasive history, impact and management of the red king crab *Paralithodes camtschaticus* off the coast of Norway In In the wrong place – Alien marine crustaceans: Distribution, biology and impacts (pp. 521–536). Dordrecht, Netherlands: Springer 10.1007/978-94-007-0591-3_18

[gcb15159-bib-0098] Jueterbock, A. , Smolina, I. , Coyer, J. A. , & Hoarau, G. (2016). The fate of the Arctic seaweed *Fucus distichus* under climate change: An ecological niche modeling approach. Ecology and Evolution, 6(6), 1712–1724. 10.1002/ece3.2001 27087933 PMC4801954

[gcb15159-bib-0099] Jueterbock, A. , Tyberghein, L. , Verbruggen, H. , Coyer, J. A. , Olsen, J. L. , & Hoarau, G. (2013). Climate change impact on seaweed meadow distribution in the North Atlantic rocky intertidal. Ecology and Evolution, 3(5), 1356–1373. 10.1002/ece3.541 23762521 PMC3678489

[gcb15159-bib-0100] Kaluza, P. , Kölzsch, A. , Gastner, M. T. , & Blasius, B. (2010). The complex network of global cargo ship movements. Journal of the Royal Society Interface, 7(48), 1093–1103. 10.1098/rsif.2009.0495 PMC2880080 20086053

[gcb15159-bib-0101] Kaschner K., Kesner‐Reyes K., Garilao C., Rius‐Barile J., Rees T., & Froese R. (Eds.). (2016). AquaMaps: Predicted range maps for aquatic species. Retrieved from www.aquamaps.org, Version 08/2016

[gcb15159-bib-0102] Katsanevakis, S. , Coll, M. , Piroddi, C. , Steenbeek, J. , Ben Rais Lasram, F. , Zenetos, A. , & Cardoso, A. C. (2014). Invading the Mediterranean Sea: Biodiversity patterns shaped by human activities. Frontiers in Marine Science, 1, 32 10.3389/fmars.2014.00032

[gcb15159-bib-0103] Kelly, R. , Leach, K. , Cameron, A. , Maggs, C. A. , & Reid, N. (2014). Combining global climate and regional landscape models to improve prediction of invasion risk. Diversity and Distributions, 20(8), 884–894. 10.1111/ddi.12194

[gcb15159-bib-0104] Kenchington, E. , Link, H. , Roy, V. , Archambault, P. , Siferd, T. , Treble, M. , & Wareham, V. (2011). Identification of mega‐ and macrobenthic ecologically and biologically significant areas (EBSAs) in the Hudson Bay Complex, the Western and Eastern Canadian Arctic. Canadian Science Advisory Secretariat Research Document (2011/071). Retrieved from http://publications.gc.ca/site/eng/454975/publication.html

[gcb15159-bib-0105] Kordas, R. L. , Harley, C. D. G. , & O'Connor, M. I. (2011). Community ecology in a warming world: The influence of temperature on interspecific interactions in marine systems. Journal of Experimental Marine Biology and Ecology, 400(1–2), 218–226. 10.1016/j.jembe.2011.02.029

[gcb15159-bib-0106] Kube, S. , Postel, L. , Honnef, C. , & Augustin, C. B. (2007). *Mnemiopsis leidyi* in the Baltic Sea: Distribution and overwintering between autumn 2006 and spring 2007. Aquatic Invasions, 2(2), 137–145. 10.3391/ai.2007.2.2.9

[gcb15159-bib-0107] Lacoursière‐Roussel, A. , Howland, K. , Normandeau, E. , Grey, E. , Archambault, P. , Deiner, K. , … Bernatchez, L. (2018). eDNA metabarcoding as a new surveillance tool for coastal Arctic biodiversity. Ecology and Evolution, 8(16), 7763–7777. 10.1002/ece3.4213 30250661 PMC6144963

[gcb15159-bib-0108] Laget, F. (2017). Transport d'espèces de dinoflagellés potentiellement non‐indigènes dans l'Arctique canadien, suite au déversement des eaux de ballast par un navire domestique. Master's thesis. Retrieved from http://semaphore.uqar.ca/1342/

[gcb15159-bib-0109] Lambert, G. , Shenkar, N. , & Swalla, B. J. (2010). First Pacific record of the north Atlantic ascidian *Molgula citrine* – Bioinvasion or circumpolar distribution. Aquatic Invasions, 5(4), 369–378. 10.3391/ai.2010.5.4.06

[gcb15159-bib-0110] Leduc, N. , Lacoursière‐Roussel, A. , Howland, K. L. , Archambault, P. , Sevellec, M. , Normandeau, E. , … Bernatchez, L. (2019). Comparing eDNA metabarcoding and species collection for documenting Arctic metazoan biodiversity. Environmental DNA, 1(4), 342–358. 10.1002/edn3.35

[gcb15159-bib-0111] Leidenberger, S. , De Giovanni, R. , Kulawik, R. , Williams, A. R. , & Bourlat, S. J. (2015). Mapping present and future potential distribution patterns for a meso‐grazer guild in the Baltic Sea. Journal of Biogeography, 42(2), 241–254. 10.1111/jbi.12395 25653464 PMC4305211

[gcb15159-bib-0112] Leidenberger, S. , Obst, M. , Kulawik, R. , Stelzer, K. , Heyer, K. , Hardisty, A. , & Bourlat, S. J. (2015). Evaluating the potential of ecological niche modelling as a component in marine non‐indigenous species risk assessments. Marine Pollution Bulletin, 97(1–2), 470–487. 10.1016/j.marpolbul.2015.04.033 26066862

[gcb15159-bib-0113] Levitus, S. , Antonov, J. I. , Boyer, T. P. , Baranova, O. K. , Garcia, H. E. , Locarnini, R. A. , … Yarosh, E. S. (2012). World ocean heat content and thermosteric sea level change (0–2000 m), 1955–2010. Geophysical Research Letters, 39(10). 10.1029/2012GL051106

[gcb15159-bib-0114] Li, X. , Liu, X. , Kraus, F. , Tingley, R. , & Li, Y. (2016). Risk of biological invasions is concentrated in biodiversity hotspots. Frontiers in Ecology and the Environment, 14(8), 411–417. 10.1002/fee.1321

[gcb15159-bib-0115] Lind, S. , Ingvaldsen, R. B. , & Furevik, T. (2018). Arctic warming hotspot in the northern Barents Sea linked to declining sea‐ice import. Nature Climate Change, 8(7), 634 10.1038/s41558-018-0205-y

[gcb15159-bib-0116] Liu, C. , White, M. , & Newell, G. (2013). Selecting thresholds for the prediction of species occurrence with presence‐only data. Journal of Biogeography, 40(4), 778–789. 10.1111/jbi.12058

[gcb15159-bib-0117] Lobo, J. M. (2008). More complex distribution models or more representative data? Biodiversity Informatics, 5, 14–19. 10.17161/bi.v5i0.40

[gcb15159-bib-0118] Locke, A. , & Hanson, J. M. (2009). Rapid response to nonindigenous species. 3. A proposed framework. Aquatic Invasions, 4(1), 259–273. 10.3391/ai.2009.4.1.26

[gcb15159-bib-0119] Locke, A. , Mandrak, N. E. , & Therriault, T. W. (2011). A Canadian rapid response framework for aquatic invasive species. Canadian Science Advisory Secretariat Research Document (2010/114). Retrieved from http://publications.gc.ca/site/eng/404076/publication.html

[gcb15159-bib-0120] Lockwood, J. L. , Hoopes, M. F. , & Marchetti, M. P. (2007). Invasion ecology. Oxford, UK: Blackwell Publishing.

[gcb15159-bib-0121] Lowen, J. B. , McKindsey, C. W. , Therriault, T. W. , & DiBacco, C. (2016). Effects of spatial resolution on predicting the distribution of aquatic invasive species in nearshore marine environments. Marine Ecology Progress Series, 556, 17–30. 10.3354/meps11765

[gcb15159-bib-0122] MacDonald, I. R. , Bluhm, B. A. , Iken, K. , Gagaev, S. , & Strong, S. (2010). Benthic macrofauna and megafauna assemblages in the Arctic deep‐sea Canada Basin. Deep Sea Research Part II: Topical Studies in Oceanography, 57(1), 136–152. 10.1016/j.dsr2.2009.08.012

[gcb15159-bib-0123] Mackey, A. P. , Atkinson, A. , Hill, S. L. , Ward, P. , Cunningham, N. J. , Johnston, N. M. , & Murphy, E. J. (2012). Antarctic macrozooplankton of the southwest Atlantic sector and Bellingshausen Sea: Baseline historical distributions (Discovery Investigations, 1928–1935) related to temperature and food, with projections for subsequent ocean warming. Deep Sea Research Part II: Topical Studies in Oceanography, 59, 130–146. 10.1016/j.dsr2.2011.08.011

[gcb15159-bib-0124] Marbuah, G. , Gren, I.‐M. , & McKie, B. (2014). Economics of harmful invasive species: A review. Diversity, 6(3), 500–523. 10.3390/d6030500

[gcb15159-bib-0125] Marcelino, V. R. , & Verbruggen, H. (2015). Ecological niche models of invasive seaweeds. Journal of Phycology, 51(4), 606–620. 10.1111/jpy.12322 26986785

[gcb15159-bib-0126] Marchese, C. (2015). Biodiversity hotspots: A shortcut for a more complicated concept. Global Ecology and Conservation, 3, 297–309. 10.1016/j.gecco.2014.12.008

[gcb15159-bib-0127] McCarthy, A. H. , Peck, L. S. , Hughes, K. A. , & Aldridge, D. C. (2019). Antarctica: The final frontier for marine biological invasions. Global Change Biology, 25(7), 2221–2241. 10.1111/gcb.14600 31016829 PMC6849521

[gcb15159-bib-0128] Meißner, K. , Fiorentino, D. , Schnurr, S. , Martinez Arbizu, P. , Huettmann, F. , Holst, S. , … Svavarsson, J. (2014). Distribution of benthic marine invertebrates at northern latitudes – An evaluation applying multi‐algorithm species distribution models. Journal of Sea Research, 85, 241–254. 10.1016/j.seares.2013.05.007

[gcb15159-bib-0129] Melia, N. , Haines, K. , & Hawkins, E. (2016). Sea ice decline and 21st century trans‐Arctic shipping routes. Geophysical Research Letters, 43(18), 9720–9728. 10.1002/2016GL069315

[gcb15159-bib-0130] Merow, C. , Smith, M. J. , & Silander, J. A. (2013). A practical guide to MaxEnt for modeling species' distributions: What it does, and why inputs and settings matter. Ecography, 36(10), 1058–1069. 10.1111/j.1600-0587.2013.07872.x

[gcb15159-bib-0131] Miller, A. W. , & Ruiz, G. M. (2014). Arctic shipping and marine invaders. Nature Climate Change, 4(6), 413–416. 10.1038/nclimate2244

[gcb15159-bib-0132] Molnar, J. L. , Gamboa, R. L. , Revenga, C. , & Spalding, M. D. (2008). Assessing the global threat of invasive species to marine biodiversity. Frontiers in Ecology and the Environment, 6(9), 485–492. 10.1890/070064

[gcb15159-bib-0133] Moore, A. M. , Lowen, J. B. , & DiBacco, C. (2018). Assessing invasion risk of *Didemnum vexillum* to Atlantic Canada. Management of Biological Invasions, 9(1), 11–25. 10.3391/mbi.2018.9.1.02

[gcb15159-bib-0134] Moore, C. M. , Mills, M. M. , Arrigo, K. R. , Berman‐Frank, I. , Bopp, L. , Boyd, P. W. , … Ulloa, O. (2013). Processes and patterns of oceanic nutrient limitation. Nature Geoscience, 6(9), 701–710. 10.1038/ngeo1765

[gcb15159-bib-0135] Moss, R. , Babiker, W. , Brinkman, S. , Calvo, E. , Carter, T. , Edmonds, J. , … Jones, R. N. (2008). Towards new scenarios for the analysis of emissions: Climate change, impacts and response strategies. Geneva, Switzerland: Intergovernmental Panel on Climate Change Secretariat (IPCC) Retrieved from https://www.ipcc.ch/publication/ipcc‐expert‐meeting‐report‐towards‐new‐scenarios‐for‐analysis‐of‐emissions‐climate‐change‐impacts‐and‐response‐strategies/

[gcb15159-bib-0136] Myers, N. , Mittermeier, R. A. , Mittermeier, C. G. , Da Fonseca, G. A. B. , & Kent, J. (2000). Biodiversity hotspots for conservation priorities. Nature, 403(6772), 853 10.1038/35002501 10706275

[gcb15159-bib-0137] Niederdrenk, A. L. , & Notz, D. (2018). Arctic sea ice in a 1.5 C warmer world. Geophysical Research Letters, 45(4), 1963–1971. 10.1002/2017GL076159

[gcb15159-bib-0138] Niemi, A. , Ferguson, S. , Hedges, K. , Melling, H. , Michel, C. , Ayles, B. , … Zimmerman, S. (2019). State of Canada's Arctic seas. Canadian Technical Report of Fisheries and Aquatic Science 3344:xv + 189 pp.

[gcb15159-bib-0139] Niimi, A. J. (2004). Environmental and economic factors can increase the risk of exotic species introductions to the Arctic region through increased ballast water discharge. Environmental Management, 33(5), 712–718. 10.1007/s00267-004-3072-4 15503388

[gcb15159-bib-0140] O'Donnell, J. , Gallagher, R. V. , Wilson, P. D. , Downey, P. O. , Hughes, L. , & Leishman, M. R. (2012). Invasion hotspots for non‐native plants in Australia under current and future climates. Global Change Biology, 18(2), 617–629. 10.1111/j.1365-2486.2011.02537.x

[gcb15159-bib-0141] Overland, J. , Dunlea, E. , Box, J. E. , Corell, R. , Forsius, M. , Kattsov, V. , … Wang, M. (2019). The urgency of Arctic change. Polar Science, 21, 6–13. 10.1016/j.polar.2018.11.008

[gcb15159-bib-0142] Pascual, M. , Fuentes, V. , Canepa, A. , Atienza, D. , Gili, J. M. , & Purcell, J. E. (2015). Temperature effects on asexual reproduction of the scyphozoan *Aurelia aurita* sl: Differences between exotic (Baltic and Red seas) and native (Mediterranean Sea) populations. Marine Ecology, 36(4), 994–1002. 10.1111/maec.12196

[gcb15159-bib-0143] Pearce, F. , Peeler, E. , & Stebbing, P. (2012). Modelling the risk of the introduction and spread of non‐indigenous species in the UK and Ireland. (Project report for E5405W). Centre for Environment, Fisheries and Aquaculture Science (Cefas). Retrieved from http://www.nonnativespecies.org/downloadDocument.cfm?id=791

[gcb15159-bib-0144] Pearson, R. G. (2007). Species' distribution modeling for conservation educators and practitioners. Synthesis. American Museum of Natural History. Retrieved from http://ncep.amnh.org

[gcb15159-bib-0145] Peck, L. S. (2005). Prospects for survival in the Southern Ocean: Vulnerability of benthic species to temperature change. Antarctic Science, 17(4), 497–507. 10.1017/S0954102005002920

[gcb15159-bib-0146] Pendleton, S. L. , Miller, G. H. , Lifton, N. , Lehman, S. J. , Southon, J. , Crump, S. E. , & Anderson, R. S. (2019). Rapidly receding Arctic Canada glaciers revealing landscapes continuously ice‐covered for more than 40,000 years. Nature Communications, 10(1), 445 10.1038/s41467-019-08307-w PMC6347664 30683866

[gcb15159-bib-0147] Phillips, S. J. , Anderson, R. P. , & Schapire, R. E. (2006). Maximum entropy modeling of species geographic distributions. Ecological Modelling, 190(3), 231–259. 10.1016/j.ecolmodel.2005.03.026

[gcb15159-bib-0148] Phillips, S. J. , & Dudík, M. (2008). Modeling of species distributions with Maxent: New extensions and a comprehensive evaluation. Ecography, 31(2), 161–175. 10.1111/j.0906-7590.2008.5203.x

[gcb15159-bib-0149] Phillips, S. J. , Dudík, M. , Elith, J. , Graham, C. H. , Lehmann, A. , Leathwick, J. , & Ferrier, S. (2009). Sample selection bias and presence‐only distribution models: Implications for background and pseudo‐absence data. Ecological Applications, 19(1), 181–197. 10.1890/07-2153.1 19323182

[gcb15159-bib-0150] Piepenburg, D. , Archambault, P. , Ambrose, W. G. , Blanchard, A. L. , Bluhm, B. A. , Carroll, M. L. , … Włodarska‐Kowalczuk, M. (2011). Towards a pan‐Arctic inventory of the species diversity of the macro‐and megabenthic fauna of the Arctic shelf seas. Marine Biodiversity, 41(1), 51–70. 10.1007/s12526-010-0059-7

[gcb15159-bib-0151] Poloczanska, E. S. , Brown, C. J. , Sydeman, W. J. , Kiessling, W. , Schoeman, D. S. , Moore, P. J. , … Duarte, C. M. (2013). Global imprint of climate change on marine life. Nature Climate Change, 3(10), 919–925. 10.1038/nclimate1958

[gcb15159-bib-0152] Poloczanska, E. S. , Burrows, M. T. , Brown, C. J. , García Molinos, J. , Halpern, B. S. , Hoegh‐Guldberg, O. , … Sydeman, W. J. (2016). Responses of marine organisms to climate change across oceans. Frontiers in Marine Science, 3, 62 10.3389/fmars.2016.00062

[gcb15159-bib-0153] R Core Team . (2019). R: A language and environment for statistical computing. Vienna, Austria: R Foundation for statistical computing Retrieved from https://www.R‐project.org/

[gcb15159-bib-0154] Ramírez, F. , Afán, I. , Davis, L. S. , & Chiaradia, A. (2017). Climate impacts on global hot spots of marine biodiversity. Science Advances, 3(2), e1601198 10.1126/sciadv.1601198 28261659 PMC5321448

[gcb15159-bib-0155] Reiss, H. , Birchenough, S. , Borja, A. , Buhl‐Mortensen, L. , Craeymeersch, J. , Dannheim, J. , … Degraer, S. (2014). Benthos distribution modelling and its relevance for marine ecosystem management. ICES Journal of Marine Science, 72(2), 297–315. 10.1093/icesjms/fsu107

[gcb15159-bib-0156] Reiss, H. , Cunze, S. , König, K. , Neumann, H. , & Kröncke, I. (2011). Species distribution modelling of marine benthos: A North Sea case study. Marine Ecology Progress Series, 442, 71–86. 10.3354/meps09391

[gcb15159-bib-0157] Renaud, P. E. , Wallhead, P. , Kotta, J. , Włodarska‐Kowalczuk, M. , Bellerby, R. G. J. , Rätsep, M. , … Kukliński, P. (2019). Arctic Sensitivity? Suitable habitat for benthic taxa is surprisingly robust to climate change. Frontiers in Marine Science, 6, 538 10.3389/fmars.2019.00538

[gcb15159-bib-0158] Ricciardi, A. , Blackburn, T. M. , Carlton, J. T. , Dick, J. T. A. , Hulme, P. E. , Iacarella, J. C. , … Aldridge, D. C. (2017). Invasion science: A horizon scan of emerging challenges and opportunities. Trends in Ecology & Evolution, 32(6), 464–474. 10.1016/j.tree.2017.03.007 28395941

[gcb15159-bib-0159] Ricciardi, A. , Hoopes, M. F. , Marchetti, M. P. , & Lockwood, J. L. (2013). Progress toward understanding the ecological impacts of nonnative species. Ecological Monographs, 83(3), 263–282. 10.1890/13-0183.1

[gcb15159-bib-0160] Richardson, M. G. (1979). The distribution of Antarctic marine macroalgae related to depth and substrate. British Antarctic Survey Bulletin, 49, 1–13.

[gcb15159-bib-0161] Rilov, G. , & Galil, B. (2009). Marine bioinvasions in the Mediterranean Sea–History, distribution and ecology In Rilov G. & Crooks J. A. (Eds.), Biological invasions in marine ecosystems (pp. 549–575). Berlin, Germany: Springer.

[gcb15159-bib-0162] Robinson, L. M. , Elith, J. , Hobday, A. J. , Pearson, R. G. , Kendall, B. E. , Possingham, H. P. , & Richardson, A. J. (2011). Pushing the limits in marine species distribution modelling: Lessons from the land present challenges and opportunities. Global Ecology and Biogeography, 20(6), 789–802. 10.1111/j.1466-8238.2010.00636.x

[gcb15159-bib-0163] Robinson, N. M. , Nelson, W. A. , Costello, M. J. , Sutherland, J. E. , & Lundquist, C. J. (2017). A systematic review of marine‐based species distribution models (SDMs) with recommendations for best practice. Frontiers in Marine Science, 4, 421 10.3389/fmars.2017.00421

[gcb15159-bib-0164] Ruiz, G. M. , Fofonoff, P. W. , Steves, B. P. , & Carlton, J. T. (2015). Invasion history and vector dynamics in coastal marine ecosystems: A North American perspective. Aquatic Ecosystem Health & Management, 18(3), 299–311. 10.1080/14634988.2015.1027534

[gcb15159-bib-0165] Ruiz, G. M. , Fofonoff, P. W. , Steves, B. , Foss, S. F. , & Shiba, S. N. (2011). Marine invasion history and vector analysis of California: A hotspot for western North America. Diversity and Distributions, 17(2), 362–373. 10.1111/j.1472-4642.2011.00742.x

[gcb15159-bib-0166] Ruiz, G. M. , & Hewitt, C. (2009). Latitudinal patterns of biological invasions in marine ecosystems: A polar perspective In Krupnik I., Lang M. A., & Miller S. E. (Eds.), Proceedings of Smithsonian at the poles: Contributions to international polar year science (pp. 347–358). IPY 10.5479/si.097884601X.26

[gcb15159-bib-0167] Sardain, A. , Sardain, E. , & Leung, B. (2019). Global forecasts of shipping traffic and biological invasions to 2050. Nature Sustainability, 2(4), 274–282. 10.1038/s41893-019-0245-y

[gcb15159-bib-0168] Screen, J. A. (2018). Arctic sea ice at 1.5 and 2° C. Nature Climate Change, 8(5), 362–363

[gcb15159-bib-0169] Semmens, B. X. , Buhle, E. R. , Salomon, A. K. , & Pattengill‐Semmens, C. V. (2004). A hotspot of non‐native marine fishes: Evidence for the aquarium trade as an invasion pathway. Marine Ecology Progress Series, 266, 239–244. 10.3354/meps266239

[gcb15159-bib-0170] Seneviratne, S. I. , Rogelj, J. , Séférian, R. , Wartenburger, R. , Allen, M. R. , Cain, M. , … Warren, R. F. (2018). The many possible climates from the Paris Agreement's aim of 1.5 C warming. Nature, 558(7708), 41 10.1038/s41586-018-0181-4 29875489

[gcb15159-bib-0171] Sigmond, M. , Fyfe, J. C. , & Swart, N. C. (2018). Ice‐free Arctic projections under the Paris Agreement. Nature Climate Change, 8(5), 404–408. 10.1038/s41558-018-0124-y

[gcb15159-bib-0172] Simberloff, D. , Martin, J.‐L. , Genovesi, P. , Maris, V. , Wardle, D. A. , Aronson, J. , … Vilà, M. (2013). Impacts of biological invasions: What's what and the way forward. Trends in Ecology & Evolution, 28(1), 58–66. 10.1016/j.tree.2012.07.013 22889499

[gcb15159-bib-0173] Smith, A. B. , & Santos, M. J. (2019). Testing the ability of species distribution models to infer variable importance. bioRxiv, 715904 10.1101/715904

[gcb15159-bib-0174] Smith, L. C. , & Stephenson, S. R. (2013). New Trans‐Arctic shipping routes navigable by midcentury. Proceedings of the National Academy of Sciences of the United States of America, 110(13), E1191–E1195. 10.1073/pnas.1214212110 23487747 PMC3612651

[gcb15159-bib-0175] Snickars, M. , Gullström, M. , Sundblad, G. , Bergström, U. , Downie, A. L. , Lindegarth, M. , & Mattila, J. (2014). Species–environment relationships and potential for distribution modelling in coastal waters. Journal of Sea Research, 85, 116–125. 10.1016/j.seares.2013.04.008

[gcb15159-bib-0176] Solecki, W. , Rosenzweig, C. , Dhakal, S. , Roberts, D. , Barau, A. S. , Schultz, S. , & Ürge‐Vorsatz, D. (2018). City transformations in a 1.5 C warmer world. Nature Climate Change, 8(3), 177–181. 10.1038/s41558-018-0101-5

[gcb15159-bib-0177] Spalding, M. D. , Fox, H. E. , Allen, G. R. , Davidson, N. , Ferdaña, Z. A. , Finlayson, M. , … Robertson, J. (2007). Marine ecoregions of the world: A bioregionalization of coastal and shelf areas. BioScience, 57(7), 573–583. 10.1641/B570707

[gcb15159-bib-0178] Speer, L. , & Laughlin, T. L. (2011). IUCN/NRDC workshop to identify areas of ecological and biological significance or vulnerability in the Arctic marine environment: Workshop report. Workshop organized by International union for the Conservation of Nature, Natural Resources Defense Council. La Jolla, USA. Retrieved from https://portals.iucn.org/library/node/12787

[gcb15159-bib-0179] Stammerjohn, S. , Massom, R. , Rind, D. , & Martinson, D. (2012). Regions of rapid sea ice change: An inter‐hemispheric seasonal comparison. Geophysical Research Letters, 39(6). 10.1029/2012GL050874

[gcb15159-bib-0180] Stelzer, K. , Heyer, K. , Bourlat, S. , & Obst, M. (2013). Application of niche modeling and earth observation for the risk assessment and monitoring of invasive species in the Baltic Sea. MarCoast II‐Marine and Coastal Environmental Information Services Ballast Water Option. Report to the European Space Agency. Retrieved from https://zenodo.org/record/886349#.XrSZtflKj3g

[gcb15159-bib-0181] Stephenson, S. A. , & Hartwig, L. (2010). The Arctic marine workshop. Canadian Manuscript Report of Fisheries and Aquatic Sciences (2934). Retrieved from http://www.dfo‐mpo.gc.ca/Library/341178.pdf

[gcb15159-bib-0182] Streftaris, N. , Zenetos, A. , & Papathanassiou, E. (2005). Globalisation in marine ecosystems: The story of non‐indigenous marine species across European seas. Oceanography and Marine Biology: An Annual Review, 43, 419–453. 10.1201/9781420037449.ch8

[gcb15159-bib-0183] Svavarsson, J. , & Dungal, P. (2008). Leyndardómar sjávarins við Ísland. Reykjavík, Iceland.

[gcb15159-bib-0184] Templeman, N. D. (2007). Placentia Bay‐Grand Banks large ocean management area ecologically and biologically significant areas. Canadian Science Advisory Secretariat Research Document (2007/052). Retrieved from http://www.icomnl.ca/files/CSAS%20Report%20PBGB%20EBSAs.PDF

[gcb15159-bib-0185] Therriault, T. W. , Nelson, J. C. , Carlton, J. T. , Liggan, L. , Otani, M. , Kawai, H. , … Clarke Murray, C. (2018). The invasion risk of species associated with Japanese tsunami marine debris in Pacific North America and Hawaii. Marine Pollution Bulletin, 132, 82–89. 10.1016/j.marpolbul.2017.12.063 29395102

[gcb15159-bib-0186] Thuiller, W. , Georges, D. , Engler, R. , & Breiner, F. (2020). biomod2: Ensemble platform for species distribution modeling. R package version 3.4.6. Retrieved from https://CRAN.R‐project.org/package=biomod2

[gcb15159-bib-0187] Thuiller, W. , Lafourcade, B. , Engler, R. , & Araújo, M. B. (2009). BIOMOD – A platform for ensemble forecasting of species distributions. Ecography, 32, 369–373. 10.1111/j.1600-0587.2008.05742.x

[gcb15159-bib-0188] Tidbury, H. J. , Taylor, N. G. H. , Copp, G. H. , Garnacho, E. , & Stebbing, P. D. (2016). Predicting and mapping the risk of introduction of marine non‐indigenous species into Great Britain and Ireland. Biological Invasions, 18(11), 3277–3292. 10.1007/s10530-016-1219-x

[gcb15159-bib-0189] Torres, U. , Godsoe, W. , Buckley, H. L. , Parry, M. , Lustig, A. , & Worner, S. P. (2018). Using niche conservatism information to prioritize hotspots of invasion by non‐native freshwater invertebrates in New Zealand. Diversity and Distributions, 24(12), 1802–1815. 10.1111/ddi.12818

[gcb15159-bib-0190] Tovar‐Sánchez, A. , Duarte, C. M. , Alonso, J. C. , Lacorte, S. , Tauler, R. , & Galbán‐Malagón, C. (2010). Impacts of metals and nutrients released from melting multiyear Arctic sea ice. Journal of Geophysical Research: Oceans, 115(C7). 10.1029/2009JC005685

[gcb15159-bib-0191] Townhill, B. L. , Tinker, J. , Jones, M. , Pitois, S. , Creach, V. , Simpson, S. D. , … Pinnegar, J. K. (2018). Harmful algal blooms and climate change: Exploring future distribution changes. ICES Journal of Marine Science, 75(6), 1882–1893. 10.1093/icesjms/fsy113

[gcb15159-bib-0192] Tremblay, P. (2017). Évaluation du risque potentiel d'introduction d'espèces non‐indigènes de mésozooplancton suite au déversement des eaux de ballast d'un navire domestique dans l'Arctique canadien. Master's thesis. Retrieved from http://semaphore.uqar.ca/1300/

[gcb15159-bib-0193] Turbelin, A. J. , Malamud, B. D. , & Francis, R. A. (2017). Mapping the global state of invasive alien species: Patterns of invasion and policy responses. Global Ecology and Biogeography, 26(1), 78–92. 10.1111/geb.12517

[gcb15159-bib-0194] Valle, M. , Chust, G. , del Campo, A. , Wisz, M. S. , Olsen, S. M. , Garmendia, J. M. , & Borja, Á. (2014). Projecting future distribution of the seagrass *Zostera noltii* under global warming and sea level rise. Biological Conservation, 170, 74–85. 10.1016/j.biocon.2013.12.017

[gcb15159-bib-0195] Verbruggen, H. , Tyberghein, L. , Belton, G. S. , Mineur, F. , Jueterbock, A. , Hoarau, G. , … De Clerck, O. (2013). Improving transferability of introduced species' distribution models: New tools to forecast the spread of a highly invasive seaweed. PLoS ONE, 8(6), e68337 10.1371/journal.pone.0068337 23950789 PMC3732097

[gcb15159-bib-0196] Verlaque, M. (2001). Checklist of the macroalgae of Thau Lagoon (Hérault, France), a hot spot of marine species introduction in Europe. Oceanologica Acta, 24(1), 29–49. 10.1016/S0399-1784(00)01127-0

[gcb15159-bib-0197] Villarino, E. , Chust, G. , Licandro, P. , Butenschön, M. , Ibaibarriaga, L. , Larrañaga, A. , & Irigoien, X. (2015). Modelling the future biogeography of North Atlantic zooplankton communities in response to climate change. Marine Ecology Progress Series, 531, 121–142. 10.3354/meps11299

[gcb15159-bib-0198] Wagner, F. J. E. (1977). Recent mollusc distribution patterns and palaeobathymetry, southeastern Beaufort Sea. Canadian Journal of Earth Sciences, 14(9), 2013–2028. 10.1139/e77-173

[gcb15159-bib-0199] Ware, C. , Berge, J. , Jelmert, A. , Olsen, S. M. , Pellissier, L. , Wisz, M. , … Alsos, I. G. (2016). Biological introduction risks from shipping in a warming Arctic. Journal of Applied Ecology, 53, 340–349. 10.1111/1365-2664.12566

[gcb15159-bib-0200] Wassmann, P. , Duarte, C. M. , Agusti, S. , & Sejr, M. K. (2011). Footprints of climate change in the Arctic marine ecosystem. Global Change Biology, 17(2), 1235–1249. 10.1111/j.1365-2486.2010.02311.x

[gcb15159-bib-0201] Weinert, M. , Mathis, M. , Kröncke, I. , Neumann, H. , Pohlmann, T. , & Reiss, H. (2016). Modelling climate change effects on benthos: Distributional shifts in the North Sea from 2001 to 2099. Estuarine, Coastal and Shelf Science, 175, 157–168. 10.1016/j.ecss.2016.03.024

[gcb15159-bib-0202] Wells, M. L. , Mayer, L. M. , & Guillard, R. R. L. (1991). Evaluation of iron as a triggering factor for red tide blooms. Marine Ecology Progress Series, 69, 93–102. 10.3354/meps069093

[gcb15159-bib-0203] Wisz, M. S. , Broennimann, O. , Grønkjær, P. , Møller, P. R. , Olsen, S. M. , Swingedouw, D. , … Pellissier, L. (2015). Arctic warming will promote Atlantic‐Pacific fish interchange. Nature Climate Change, 5(3), 261–265. 10.1038/nclimate2500

[gcb15159-bib-0204] Wisz, M. S. , Pottier, J. , Kissling, W. D. , Pellissier, L. , Lenoir, J. , Damgaard, C. F. , … Svenning, J.‐C. (2013). The role of biotic interactions in shaping distributions and realised assemblages of species: Implications for species distribution modelling. Biological Reviews of the Cambridge Philosophical Society, 88(1), 15–30. 10.1111/j.1469-185X.2012.00235.x 22686347 PMC3561684

[gcb15159-bib-0205] Xu, J. , Wickramarathne, T. L. , Chawla, N. V. , Grey, E. K. , Steinhaeuser, K. , Keller, R. P. , … Lodge, D. M. (2014). Improving management of aquatic invasions by integrating shipping network, ecological, and environmental data: data mining for social good In Proceedings of the 20th ACM SIGKDD International Conference on Knowledge Discovery and Data Mining *,* USA, 1699–1708. 10.1145/2623330.2623364

[gcb15159-bib-0206] Zimina, O. L. (2014). Finding the snow crab *Chionoecetes opilio* (O. Fabricius, 1788) (Decapoda: Majidae) in the Kara Sea. Russian Journal of Marine Biology, 40(6), 490–492. 10.1134/S1063074014060224

