# Supplementary material for: What and where? Predicting invasion hotspots in the Arctic marine realm
Source: Glob Chang Biol. 2020 Jul 10;26(9):4752–71. doi: 10.1111/gcb.15159 (PMC7496761; doi:10.1111/gcb.15159)
Supplement: Supplementary file 1 — Fig S1 [file GCB-26-4752-s001.docx]

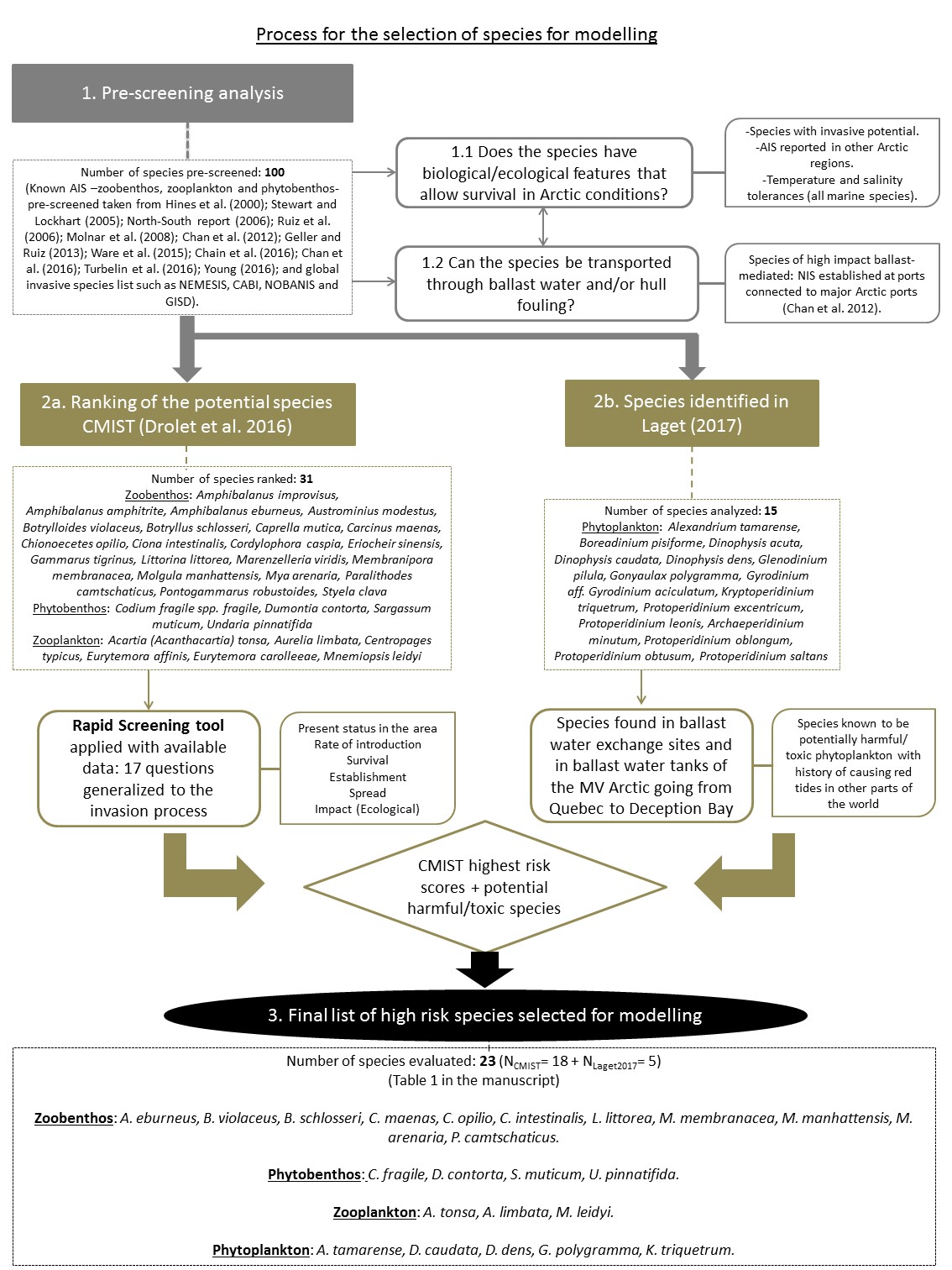


**Figure S1**: Process for selecting the species for modelling.

Note: *G. tigrinus* and *E. sinensis* were selected using CMIST but their models were not included in the present study (euryhaline species that resulted in low performance models with the environmental layers considered). Instead, three species of special interest were added: *D. contorta*, which is present in Arctic regions (Mathieson, Moore, & Short, 2010); *A. eburneus,* which has been found in hull fouling on ships arriving in Canadian Arctic ports (Chan, MacIsaac, & Bailey, 2015); and *C. intestinalis,* which has been reported in some Arctic regions and may belong to a coldwater subspecies complex - f. *gelatinosa* or f. *longissimi* - rather than *C. intestinalis* f., *typical* (Sanamyan & Sanamyan, 2007).

**References**

Chain, F. J. J., Brown, E. A., MacIsaac, H. J., & Cristescu, M. E. (2016). Metabarcoding reveals strong spatial structure and temporal turnover of zooplankton communities among marine and freshwater ports. *Diversity and Distributions, 22*(5), 493-504.

Chan, F. T., Bronnenhuber, J. E., Bradie, J. N., Howland, K., Simard, N. and Bailey, S. A. 2012. *Risk assessment for ship-mediated introductions of aquatic nonindigenous species to the Canadian Arctic*. Canadian Science Advisory Secretariat Research Document 2011/105. vi + 93 p. Retrieved from: http://publications.gc.ca/site/eng/457830/publication.html

Chan, F. T., MacIsaac, H. J., & Bailey, S. A. (2015). Relative importance of vessel hull fouling and ballast water as transport vectors of nonindigenous species to the Canadian Arctic. *Canadian Journal of Fisheries and Aquatic Sciences, 72*(8), 1230-1242.

Chan, F. T., MacIsaac, H. J., & Bailey, S. A. (2016). Survival of ship biofouling assemblages during and after voyages to the Canadian Arctic. *Marine Biology, 163*(12), 250.

Drolet, D., DiBacco, C., Locke, A., McKenzie, C. H., McKindsey, C. W., Moore, A. M., . . . Therriault, T. W. (2016). Evaluation of a new screening-level risk assessment tool applied to non-indigenous marine invertebrates in Canadian coastal waters. *Biological Invasions, 18*(1), 279-294.

Geller, J. B., & Ruiz, G. M. (2013). *Marine invasive species technical support – Quantitative survey of nonindigenous species (NIS) in Prince William Sound: Plankton*. Prince William Sound Regional Citizens’ Advisory Council, Contract number: 952.13.01. Retrieved from:

http://www.pwsrcac.org

Hines, A. H., Ruiz, G. M., & Fofonoff, P. W. (2000). *Summary of NIS in Prince William Sound and Alaska. Biological invasions of cold-water coastal ecosystems: ballast-mediated introductions in Port Valdez / Prince William Sound, Alaska*. Prince William Sound: Regional Citizens’ Advisory Council of Prince William Sound. Retrieved from: https://www.anstaskforce.gov/EcoSurveys/tech0050.pdf

Laget, F. (2017). *Transport d’espèces de dinoflagellés potentiellement non-indigènes dans l’Arctique canadien, suite au déversement des eaux de ballast par un navire domestique.* (Master's thesis), Retrieved from: http://semaphore.uqar.ca/1342/

Mathieson, A. C., Moore, G. E., & Short, F. T. (2010). A floristic comparison of seaweeds from James Bay and three contiguous northeastern Canadian Arctic sites. *Rhodora, 112*(952), 396-434.

Molnar, J. L., Gamboa, R. L., Revenga, C., & Spalding, M. D. (2008). Assessing the global threat of invasive species to marine biodiversity. *Frontiers in Ecology and the Environment, 6*(9), 485-492.

North-South Consultants (2006). *Potential dispersal of aquatic invasive species into Hudson Bay from ballast water from ships travelling from ports in Europe and North America*. File No. F2408-050083. Retrieved from: <http://www.dfo-mpo.gc.ca/Library/344957.pdf>

Ruiz, G. M., Huber, T., Larson, K., McCann, L., Steves, B., Fofonoff, P., & Hines, A. H. (2006). *Biological Invasions in Alaska's Coastal Marine Ecosystems: Establishing a Baseline.* Prince William Sound: Regional Citizens’ Advisory Council of Prince William Sound. Retrieved from: http://www.vliz.be/imisdocs/publications/238416.pdf

Sanamyan, K., & Sanamyan, N. (2007). Poorly known Ascidiacea collected in the vicinity of the Commander Islands and East Kamchatka, NW Pacific. *Zootaxa, 1579*(1), 55-68.

Stewart, D. B., & Lockhart, W. L. (2005). *An overview of the Hudson Bay marine ecosystem*. Canadian Technical Report of Fisheries and Aquatic Sciences 2586: vi + 487 p. Retrieved from: http://www.dfo-mpo.gc.ca/Library/314704.htm

Turbelin, A. J., Malamud, B. D., & Francis, R. A. (2017). Mapping the global state of invasive alien species: patterns of invasion and policy responses. *Global Ecology and Biogeography, 26*(1), 78-92.

Ware, C., Berge, J., Jelmert, A., Olsen, S. M., Pellissier, L., Wisz, M. S., . . . Alsos, I. G. (2015). Biological introduction risks from shipping in a warming Arctic. *Journal of Applied Ecology, 53*, 340-349.

Young, R. (2016). *Molecular species delimitation and biogeography of Canadian marine planktonic crustaceans* (Doctoral dissertation). Retrieved from: https://atrium.lib.uoguelph.ca/xmlui/handle/10214/9753
