# Supplementary material for: What and where? Predicting invasion hotspots in the Arctic marine realm
Source: Glob Chang Biol. 2020 Jul 10;26(9):4752–71. doi: 10.1111/gcb.15159 (PMC7496761; doi:10.1111/gcb.15159)
Supplement: Supplementary file 2 — Fig S2 [file GCB-26-4752-s002.docx]

**
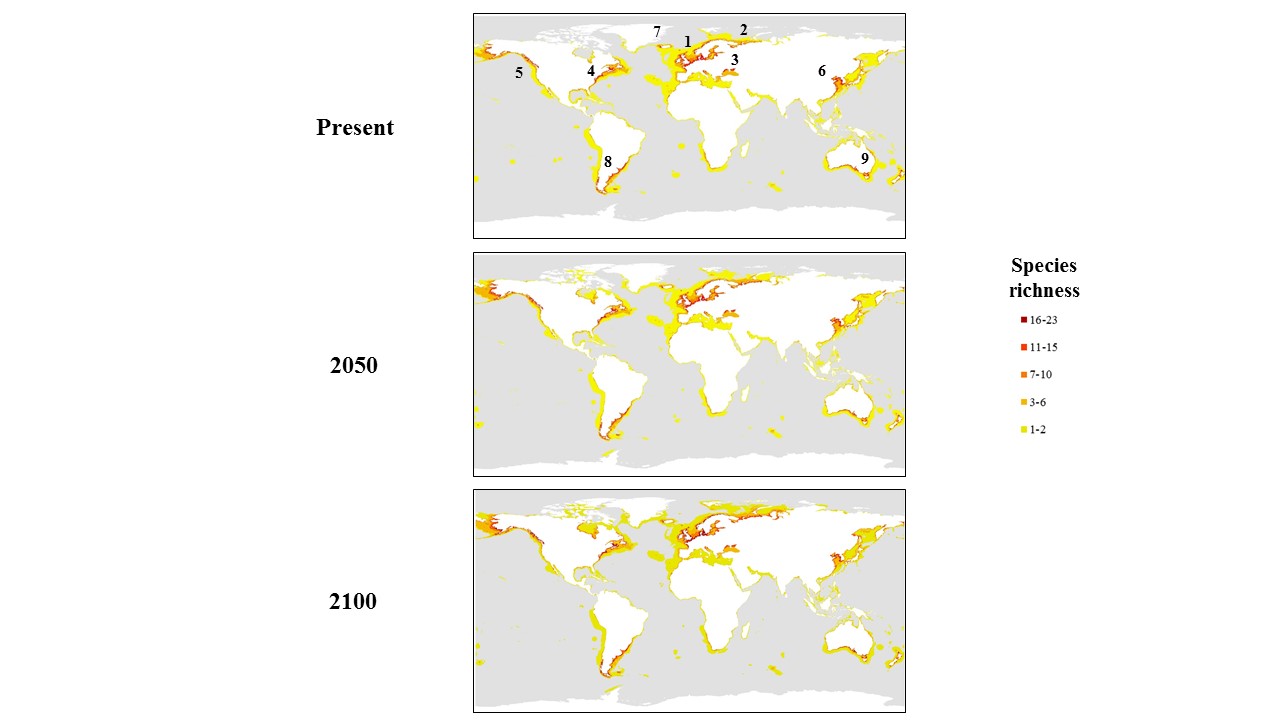
**

**Figure S2**: Predicted total species richness at a global scale. Colors represent the number of overlapped species for present and future (2050 and 2100). Realms and ecoregions as delineated in Spalding et al. (2007). 1: Northern European Seas; 2: White Sea; 3: Black Sea; 4: Northwest Atlantic; 5: Northeast Pacific; 6: Northwest Pacific; 7: Iceland; 8: Temperate South America; 9: Temperate Australasia.

**References:**

Spalding, M. D., Fox, H. E., Allen, G. R., Davidson, N., Ferdaña, Z. A., Finlayson, M. A. X., . . . Lourie, S. A. (2007). Marine ecoregions of the world: a bioregionalization of coastal and shelf areas. *Bioscience, 57*(7), 573-583.
