# Supplementary material for: What and where? Predicting invasion hotspots in the Arctic marine realm
Source: Glob Chang Biol. 2020 Jul 10;26(9):4752–71. doi: 10.1111/gcb.15159 (PMC7496761; doi:10.1111/gcb.15159)
Supplement: Supplementary file 3 — Fig S3 [file GCB-26-4752-s003.docx]

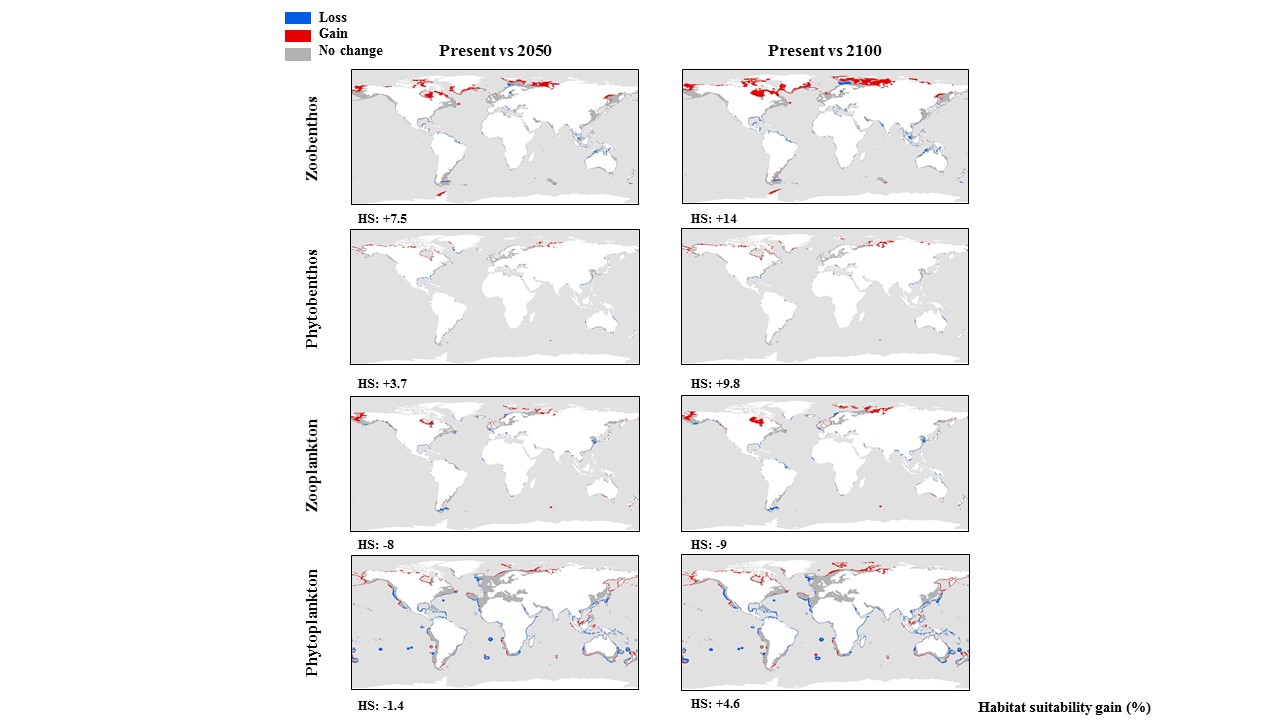


**Figure S3**: Habitat suitability change of each ecological group at a global scale comparing present *vs.* future scenarios (2050 and 2100). Percentage of gain (+) or loss (-) in habitat suitability (HS) is shown for each taxonomic group. Coloured areas correspond to suitable habitats only in the present (loss), only in the future (gain), and both in the present and the future (no change).
