# Supplementary material for: What and where? Predicting invasion hotspots in the Arctic marine realm
Source: Glob Chang Biol. 2020 Jul 10;26(9):4752–71. doi: 10.1111/gcb.15159 (PMC7496761; doi:10.1111/gcb.15159)
Supplement: Supplementary file 4 — Table S1 [file GCB-26-4752-s004.docx]

**Table S1**: Known native and non-native ranges, reported impacts and occurrence points of the species modeled. Native and non-native ranges described according to Spalding et al. (2007) nomenclature (ecoregions, province and/or realm)

| **Species** | **Native range** | **Non-native range** | **Known caused impact** | **References of impact** | **Occurrence points** | **Reference of occurrence** |
| --- | --- | --- | --- | --- | --- | --- |
| ***Amphibalanus eburneus*** | Native to the Cold and Warm Temperate Northwest Atlantic, Tropical Northwestern and Southwestern Atlantic, and Warm Temperate Southwestern Atlantic | Northern European Seas, Lusitanian, Mediterranean Sea, Western and Eastern Indo-Pacific (Bay of Bengal and, Hawaii), Warm Temperate Northeast Pacific and Tropical East Pacific | -Competition for space and food that can reduce the biomass of native species of molluscs and hydroids.  -Competition for settling space with other taxa such as barnacles and oysters. | Boudreaux, Walters, and Rittschof (2009); Saitsev and Ozturk (2001); Wolff (2005); NEMESIS | 119 | GBIF; OBIS; NEMESIS |
| ***Botrylloides violaceus*** | Cold and Warm Temperate Northwest Pacific | Cold and Warm Temperate Northeast Pacific, Cold Temperate Northwest Atlantic, Northern European Seas, Lusitanian, and Mediterranean Sea | -Reduce productivity and coverage of native eelgrass (*Zostera marina*) beds.  -Alters plant, invertebrate and possibly fish communities that could impact species dominance and diversity, and community dynamics.  -It can displace other fouling organisms, such as tunicates, bryozoans, barnacles, and mussels through competition for space and food.  It can impact habitat structure by overgrowing and smothering existing species. It can also create large areas of bare substrate for organisms to colonize.  -It can impact the food web and trophic structure of invaded ecosystems by inducing changes in plant, invertebrate and possibly fish communities.  -Under extreme conditions, water quality could be impacted by the amount of ammonia excreted by the tunicate colonies. | C. E. Carver, A. L. Mallet, and Bénékidte Vercaemer (2006a); Dijkstra and Harris (2009); Dijkstra, Harris, and Westerman (2007); McKenzie, Matheson, Caines, and Wells (2016); Simkanin et al. (2016); Therriault and Herborg (2008) and refs. therein; Wong and Vercaemer (2012); NEMESIS | 365 | Kott (2003); Vaz-Pinto et al. (2014); GBIF; OBIS |
| ***Botryllus schlosseri*** | The origin and species status are uncertain, although it is believed it is from European origin (Northern European Seas, Lusitanian, Mediterranean Sea, and Black Sea). | Cold Temperate Northeast Pacific, Warm Temperate Southeastern Pacific, Warm Temperate Southwestern Atlantic, Cold and Warm Temperate Northwest Atlantic Temperate Southern Africa (Agulhas), Central Indo-Pacific (South China Sea, Sunda Shelf, South Kuroshio, and Western Coral Triangle, Northeast and Northwest Australian Shelfs, Sahul Shelf), and Temperate Australasia (East Central, Southeast, and Southwest Australian Shelfs, Southern New Zealand) | -It can displace native species. Compete for space by overgrowing and smothering existing species, including other fouling species and algae.  -Compete for food with filter feeding taxa such as zooplankton and bivalves.  -Surface fouling could reduce productivity and coverage of eelgrass beds.  -It can alter trophic structure of aquatic ecosystems by altering plant, invertebrate, and possibly fish communities.  -Reduction in community species diversity.  -Under extreme conditions, water quality could be impacted by the amount of ammonia excreted by the tunicate colonies. | Carver et al. (2006a); Dijkstra et al. (2007); Dijkstra, Sherman, and Harris (2007); Molnar, Gamboa, Revenga, and Spalding (2008); Pederson et al. (2005); Therriault and Herborg (2008); Wong and Vercaemer (2012); NEMESIS | 1532 | Ben-Shlomo, Reem, Douek, and Rinkevich (2010); Canning-Clode, Fofonoff, McCann, Carlton, and Ruiz (2013); Mead, Carlton, Griffiths, and Rius (2011); Turon, Cañete, Sellanes, Rocha, and López-Legentil (2016); GBIF; OBIS |
| ***Carcinus maenas*** | Atlantic Europe (Northern European Seas, Lusitanian, Mediterranean Sea) | Cold Temperate Northwest Atlantic, Cold Temperate Northeast Pacific, Temperate South America (Magellanic), Temperate Southern Africa (Benguela), Easte Central and Southeast Australian Shelf, Warm Temperate Northwest Pacific | -They prey on algae, sessile and mobile epifauna, and shallowly buried infauna.  -Predation has significantly reduced many native shellfish populations.  -Can fundamentally alter marine communities.  -Bioperturbation by digging the top few centimeters of sediment can alter the natural habitat, particularly where the crabs are abundant and water is shallow, and where beds of bivalves such as soft-shelled clams or scallops are present. This can also reduce the coverage of ecologically important eelgrass beds which provide habitat that is critical to the ecology of migratory birds.  -They are host to a broad range of parasites, pathogens and epifaunal organisms. | Carlton and Cohen (2003); Cohen, Carlton, and Fountain (1995); Compton, Leathwick, and Inglis (2010); Crothers (1968); Grosholz and Ruiz (2002); Stewart and Howland (2009) | 2266 | GBIF; OBIS; NEMESIS |
| ***Chionoecetes opilio*** | Arctic / Sub-Arctic species found in Beaufort, Chukchi, and Eastern Bering Seas, Cold Temperate Northeast and Northwest Pacific, Cold Temperate Northwest Atlantic, and West Greenland Shelf | Arctic (North and East Barents Sea) | -Generalist feeders known to feed on algae, mollusks, crustaceans, polychaetes, echinoderms and fish. This can lead to shifts in energy fluxes, nutrient cycles and thus, affect critical ecosystem services, biodiversity and fisheries.  -Competition for food with other crabs and modification of the food web.  -Reduce stability of local habitats through burrowing activity. | Alvsvåg, Agnalt, and Jørstad (2009); Gilbey, Attrill, and Coleman (2008); Hänfling, Edwards, and Gherardi (2011); Lovvorn (2010); Veldhuizen and Stanish (1999); Wieczorek and Hooper (1995) | 9803 | Hansen (2015); GBIF |
| ***Ciona intestinalis*** | Difficult to determine: Europe (Northern European Seas, Lusitanian, Mediterranean Seam Black Sea)? | Temperate South America (Magellanic, Warm Temperate Southwestern Atlantic and Southeastern Pacific), Cold and Warm Temperate Northeast Pacific, Temperate Southern Africa (Benguela), Central Indo-Pacific (South China Sea), Temperate Australasia (Southwest, Southeast and East Central Australian Shelf, Southern New Zealand) | -It may outcompete species that settle later because their larger size allows for greater energetic reserves and/or greater feeding capacity.  -Dense aggregations can change species richness and community composition, with a moderate impact on biodiversity.  -Aggregations may impact food web and trophic structure of aquatic ecosystems by inducing changes in plant, invertebrate and possibly fish communities.  -Aggregations can also decrease water circulation, limiting oxygen and food. | C. E. Carver, A. L. Mallet, and B. Vercaemer (2006b); Therriault and Herborg (2008) | 961 | GBIF; OBIS |
| ***Littorina littorea*** | Northern European Seas, White Sea. Lusitanian, Mediterranean Sea | Cold Temperate Northwest Atlantic, Northern Grand Banks – Southern Labrador, Cold Temperate Northeast Pacific | -It can alter diversity, abundance, and distribution of many animal and plant species on rocky as well as soft bottom shores. It can change intertidal ecosystems via grazing activities, altering the distribution and abundance of algae on rocky shores and converting soft-sediment habitats to hard substrates.  -It competes with and may have displaced native North American *Littorina saxatilis* from portions of the mid and low intertidal zone.  -Quantity of green algae can be markedly reduced by the presence of *L. littorea*.  -Grazing can quantitatively reduce recruitment of many benthic intertidal organisms; larger sessile organisms (e.g., rockweeds) escape grazing and then benefit from being cleaned by superficial grazing of *L. littorea* on their surfaces.  -Can displace mudsnails from mudflats, affecting the composition of mudflat infauna.  -It is host to obligate and facultative endosymbionts from seven animal phyla, including Annelida, Arthropoda, Gnathostomulida, Nematoda, Nemertea, and Platyhelminthes, as well as ciliates and algae. | Brenchley and Carlton (1983); Buckland-Nicks, Chisholm, and Gibson (2013); Eastwood, Donahue, and Fowler (2007); Lubchenco (1983); Yamada and Mansour (1987); CABI; NEMESIS. | 1823 | GBIF; OBIS |
| ***Membranipora membranacea*** | Northern European Seas, Lusitanian | Cold Temperate Northwest Atlantic, Cold Temperate Northeast Pacific, Temperate South America (Warm Temperate Southwestern Atlantic and Southeastern Pacific, Magellanic), Temperate Australasia (Southwest and East Central Australian Shelf, Northern and Southern New Zealand) | -It is abundant on kelps of the genus *Laminaria*. Heavy settlement reduces kelp survival and biomass, and favours dominance by algae that provide less cover.  -Recurrent seasonal outbreaks can have devastating effect on native kelp populations, facilitating at the same time, the establishment and growth of other invasive species such as the green alga *Codium fragile*.  -Changes in habitat structure and benthic community organization. | Berman, Harris, Lambert, Buttrick, and Dufresne (1992); Chapman, Scheibling, and Chapman (2002); Harris and Tyrrell (2001); Levin, Coyer, Petrik, and Good (2002); Scheibling and Gagnon (2009); NEMESIS | 1516 | GBIF; OBIS; NEMESIS |
| ***Molgula manhattensis*** | Cold and Warm Temperate Northwest Atlantic | Northern European Seas, Black Sea, East Central and Southeast Australian Shelf, Cold Temperate Northeast and Northwest Pacific | -It can rapidly settle and overgrow most other fouling community organisms.  -It can out-compete native species for food and space, and potentially by consuming the spawn or larvae of other marine species.  -It can influence biodeposition, transport, and composition of suspended sediments in estuaries due to a high production rate of solids per unit tissue weight relative to other suspension feeders.  -It may transport viable cells and cysts of toxic phytoplankton. | Andrews (1953); Calder (1966); Otsuka and Dauer (1982); Haven and Morales‐Alamo (1966); Rosa et al. (2013) | 493 | GBIF; OBIS |
| ***Mya arenaria*** | Cold and Warm Temperate Northwest Atlantic, Northern Grand Banks- Southern Labrador | Northern European Seas, Mediterranean Sea, White Sea, Black Sea, Cold and Warm Temperate Northwest Pacific, Cold Temperate Northeast Pacific, Eastern Bering Sea | -It can compete and replace native bivalves such as reported with *Macoma nasuta* in San Francisco Bay, *Macoma balthica* in the Baltic Sea, *Lentidium mediterraneum* in the Black Sea, and *Cerastoderma edule* in the Skagerrak, Sweden.  -During periods of exceptional abundance, it can change the community, affecting phytoplankton abundance, and in turn, zooplankton, mysids, and fish recruitment.  -They can form so-called death assemblage, which can persist for 100 years and form habitats for other species.  -Powerful burrower and filterer, with the potential to alter habitats and sediment characteristics through bioturbation and deposition of peudofeces and also through suspension feeding, increasing water clarity, and light penetration. | Cohen et al. (1995); Obolewski and Piesik (2005); Skolka and Preda (2010); Strasser (1998); NEMESIS, NOBANIS | 2116 | GBIF; OBIS |
| ***Paralithodes camtschaticus*** | Cold Temperate Northeast and Northwest Pacific, Eastern Bering Sea | North and East Barents Sea, Northern European Seas (Northern Norway and Finnmark) | - It is a large general predator. It can predate on 100 different species (invertebrates, algae and fish remnants).  -Modification in the community structure: decrease in biomass of sipunculids, echinoderms and bivalves.  -Competition with fish such as huddock, plaice, wolffish and cod.  -Reduce stability of local habitats through burrowing activity.  -Feed on epibenthic organisms that play an important role in the functioning of benthic systems.  - Crab carapace is a favoured substratum for the leech *Johanssonia arctica*, a vector for a Trypanosome infection in Atlantic cod. | Anisimova, Berenboim, Gerasimova, Manushin, and Pinchukov (2005); Gilbey et al. (2008); Hemmingsen, Jansen, and MacKenzie (2005); Jørgensen (2005); Rzhavsky, Kuzmin, and Udalov ; Veldhuizen and Stanish (1999) | 686 | Dvoretsky and Dvoretsky (2009); Jørgensen and Nilssen (2011); Oug, Cochrane, Sundet, Norling, and Nilsson (2011); GBIF |
| ***Codium fragile* spp. *fragile*** | Cold and Warm Temperate Northwest Pacific | Cold and Warm Temperate Northwest Atlantic, Northern European Seas, Lusitanian, Mediterranean Sea, Temperate Australasia, Cold Temperate Northeast Pacific, Warm Temperate Southeastern Pacific | -Competition with natives, decreasing kelp cover, biomass and abundance.  -It can affect community structure and composition affecting other native species by shifting habitat selection and feeding behaviours.  -With the presence of other invader *M. membranacea*, it can reduce growth, abundance and survival of kelp, resulting in defoliation of kelp plants and displacing by gap formation in kelp beds.  -It can decrease the epifauna diversity and density, and increase the epiflora density.  -It can increase sedimentation since it is a "low lying" alga, making it difficult for some large invertebrates and fish to move among the plants and live in the space between the bushy parts of the algae and the seabed.  -Host of an epiphyte algae, *Neosiphonia harveyi*, which is also a NIS from the Pacific that has invaded the NW Atlantic.  -It can have an economic impact on shellfish and fishing industries. | Chavanich, Harris, Je, and Kang (2006); Harris and Jones (2005); Harris and Tyrrell (2001); Levin et al. (2002); Mathieson, Pederson, Neefus, Dawes, and Bray (2008); Schmidt and Scheibling (2006); Sorte, Williams, and Zerebecki (2010); Trowbridge (1999) | 99 | Armitage, Sjøtun, and Jensen (2014); Bulleri and Airoldi (2005); Carlton and Scanlon (1985); Chavanich et al. (2006); Drouin, McKindsey, and Johnson (2012); Josselyn and West (1985); Madariaga, Rivadeneira, Tala, and Thiel (2014); Mathieson, Pederson, et al. (2008); McDonald, Huisman, Hart, Dixon, and Lewis (2015); Provan, Murphy, and Maggs (2005); Scheibling and Gagnon (2006); GBIF |
| ***Dumontia contorta*** | Northern European Seas, Lusitanian, White Sea, North and East Barents Sea | Cold Temperate Northwest Atlantic, Northern Gran Banks –Southern Labrador, Hudson Complex, Cold Temperate Northeast Pacific, Cold Temperate Northwest Pacific (Sea of Japan) | -It can impact native macroalgal community structure and diversity by altering the physical, chemical, and biotic characteristics of the habitat.  -They can act as a substrate of other organisms, especially macrofauna associated with macroalgae in kelp forests such as Bryozoa, Polychaeta and Hydrozoa, and also of other invasive organisms such as *Porphyra* algae. | Dunn (1917); Mathieson, Hehre, Dawes, and Neefus (2008); Mathieson, Moore, and Short (2010); Mathieson, Pederson, et al. (2008); Neefus, Mathieson, Bray, and Yarish (2008); Nyberg (2007); Ronowicz, Włodarska‐Kowalczuk, and Kukliński (2013); Włodarska-Kowalczuk, Kukliński, Ronowicz, Legeżyńska, and Gromisz (2009) | 897 | Kozhenkova (2009); Mathieson, Pederson, et al. (2008); G. Moore pers comm 2018; GBIF; OBIS |
| ***Sargassum muticum*** | Cold and Warm Temperate Northwest Pacific | Cold and Warm Temperate Northeast Pacific, Hawaii, Northern European Seas, Lusitanian, Mediterranean Sea | -Competition for nutrients, light and space on native macroalgal species.  -Decreases kelp density, seaweed cover and biomass.  -It can change the physical, chemical, and biotic characteristics of the habitat.  -It can increase the nitrate concentration of the habitat.  -Algal drift (wrack) promotes an increase in the abundance of sandy beach macrofauna by providing a food source or shelter for small invertebrates, which may have important effects on macrofaunal assemblages and ecosystem function on sandy beaches.  -A 5 m tall plant may host an average of 3,000 animals, including foraminifers, hydroids, flatworms, polychaete worms, leeches, snails, ostracods, cumaceans, isopods, gammarid and caprellid amphipods, opossum shrimp, euphausid shrimp, crabs and bryozoans. | Nicholson et al. (1981); Nyberg (2007); Sorte et al. (2010); Rodil, Olabarria, Lastra, and López (2008); White (2010) | 523 | Cheang et al. (2010); El Atouani et al. (2016); Sabour, Reani, El Magouri, and Haroun (2013); Sfriso and Facca (2013); GBIF; OBIS |
| ***Undaria pinnatifida*** | Cold and Warm Temperate Northwest Pacific | Northern European Seas, Lusitanian, Mediterranean Sea, Cold and Warm Temperate Northeast Pacific, Warm Temperate Southwestern Atlantic, Magellanic, Southeast Australian Shelf, and Northern and Southern New Zealand | -Decrease native seaweed richness and cover and competitor abundance.  - It produces several allelopathic substances, which inhibit settlement and germination of other seaweeds (Asiatic red, brown and green seaweeds).  -It can form mats or uniform meadows, which also may change the existing architectural structure.  -It can be ecological engineers, creating a habitat that is suitable for other invasive species to settle.  -Threat to natural ecosystems and associated fisheries through displacement of native species via the development of ‘mono-specific’ *Undaria* stands. | Forrest and Taylor (2002); Murphy, Johnson, and Viard (2016); Sorte et al. (2010); Wallentinus and Nyberg (2007); CABI | 87 | Martin and Cuevas (2006); Meretta, Matula, and Casas (2012); Pereyra, Arias, González, and Narvarte (2014); Primo, Hewitt, and Campbell (2010); Schiel and Thompson (2012); Sfriso and Facca (2013); GBIF; OBIS |
| ***Acartia (Acanthacartia) tonsa*** | Cold and Warm Temperate Northwest Atlantic, Tropical Northwestern Atlantic (although it is considered a cryptogenic species with a worldwide distribution) | Northern European Seas, Black Sea. As part of its cryptogenic worldwide distribution, it has also been recorded in Mediterranean Sea, Western Indo-Pacific, Tropical Atlantic, Temperate South America, Cold Temperate Northeast Pacific | -It can compete for food with native species, and affect abundance.  -It is omnivorous, so it is capable of feeding on both phytoplankton and protozoan prey.  -It can change energy and matter flows between pelagic and benthic compartments.  -It may impact on trophic food web due to its high abundance that can induce a second period of high zooplankton production.  -It may serve as PSP toxin vectors to higher trophic level given that PSP toxin can be accumulated in copepod grazers such as *A. tonsa*.  -Significant correlation between zooplankton blooms (including *A. tonsa* individuals) and cholera cases. | Andersen Borg (2009); Bollens, Cordell, Avent, and Hooff (2002); David, Sautour, and Chardy (2007); Gaudy and Viñas (1985); Huq et al. (2005); Jonsson and Tiselius (1990); Gubanova (2000); Kurashova and Abdullayeva (1984); Leppäkoski, Olenin, and Gollasch (2002); Pienimäki and Leppäkoski (2004); Teegarden and Cembella (1996); Telesh (2008) | 223 | GBIF; OBIS |
| ***Aurelia limbata*** | Most likely native to the Cold Temperate Northwest Pacific | Cold Temperate Northwest Atlantic (although taxonomic issues with *A. aurita*) | Challenging case on genetic and taxonomy: the impacts described here are the ones known for *Aurelia* sp.  -It competes with planktonfeeding fish or preys on their juveniles. It has even been considered a keystone species in the control of trophic structures. | Korsun, Fahrni, and Pawlowski (2012); Olesen (1995); NEMESIS | 28 | Chang, Kim, Yoon, and Ki (2016); Miyake, Lindsay, Hunt, and Hamatsu (2002); Radchenko (2013); Zavolokin (2010); GBIF; OBIS |
| ***Mnemiopsis leidyi*** | Cold and Warm Temperate  Northwest Atlantic, Tropical Northwestern Atlantic, Warm Temperate Southwestern Atlantic, Magellanic | Northern European Seas, Black Sea, Mediterranean Sea | -It is a generalist carnivorous feeder, and can cause rapid decline in ichthyoplankton and mesozooplankton abundance and species diversity.  -It is a real ecosystem engineer; it affects physical conditions of several recipient productive ecosystems; for example in the decrease in water transparency, hydrochemical change nutrients contents and biota.  -It can cause invasion cascading effects at the higher trophic levels, from a decreasing zooplankton stock to collapsing planktivorous fish.  -It competes with commercial planktonfeeding fish or preys on their juveniles, creating an economic impact.  -It can serve as intermediate hosts for biota such as *Hysterothylacium* larvae, and host an endoparasitic sea anemone (*Edwardsiella lineata* larvae). | Korsun et al. (2012); Purcell, Uye, and Lo (2007); Purcell and Arai (2001); Selander, Møller, Sundberg, and Tiselius (2010); Shiganova (1998); Shiganova (2004); Tulp (2006) | 389 | Malej et al. (2017); GBIF; OBIS |
| ***Alexandrium tamarense*** | Cosmopolitan species | Difficult to characterize the geographic  distribution of native and non-native ranges | -It can produce dangerous toxins, particularly when in large numbers. It produces potent PSP neurotoxins which can affect humans, other mammals, fish and birds.  -Responsible for numerous human illnesses and several deaths after consumption of infected shellfish. | Larsen and Moestrup (1989); Okolodkov (2005); Reyes-Vasquez, Ferraz-Reyes, and Vasquez (1979); Steidinger, Tangen, and Tomas (1996); Tamiyavanich, Kodama, and Fukuyo (1985) | 528 | Carignan and Carreto (2013); Matsuno, Ichinomiya, Yamaguchi, Imai, and Kikuchi (2014); GBIF; OBIS |
| ***Dinophysis caudata*** | Widely distributed in cold and temperate waters world-wide | Difficult to characterize the geographic  distribution of native and non-native ranges | -Toxic species that produces okadaic acid (OA), as well as dinophysistoxin-1 (DTX1).  -It has been associated with diarrheic shellfish poison (DSP) outbreaks in Chile, Portugal, Scandinavia and the USA. | Larsen (1992); Lee et al. (1989); Steidinger et al. (1996); Yasumoto (1990) | 4676 | Laget (2017); GBIF; OBIS, A. Rochon pers. comm. |
| ***Dinophysis dens*** | Northern European Seas, Lusitanian | Some records in Cold Temperate Northwest Atlantic and East Central and Southeast Australian Shelf, but no information on its status in these locations | -It can produce toxins associated to DSP. Shellfish fisheries closed. | Algaebase; HAEDAT | 56 | Laget (2017); GBIF; OBIS |
| ***Gonyaulax polygramma*** | Cosmopolitan species common in cold temperate to tropical waters worldwide | Unknown | -Although it is a non-toxin producing species, it takes part of red tide species, and it has been associated with massive fish and invertebrate kills due to anoxia and high sulfide and ammonia levels resulting from cell decomposition. -Responsible for harmful algal blooms in Korea and in the Gulf of Mexico. | Gárate-Lizárraga, del Socorro Muñetón-Gómez, and Maldonado-López (2011); Hallegraeff (1991); Kim et al. (2006); Koizumi, Kohno, Matsuyama, Uchida, and Honjo (1996) | 1972 | Laget (2017); Matsuno et al. (2014); GBIF; OBIS |
| ***Kryptoperidinium triquetrum*** | Cold Temperate Northwest Atlantic, Northern European Seas, Lusitanian, Mediterranean Sea, Black Sea, White Sea, North and East Barents Sea, Kara Sea, some records in Temperate Australasia, Cold Temperate Northwest and Northeast Pacific | Unknown | -Associated to 21 events of blooms and problems with their toxins around the globe (mainly around Europe). | HAEDAT; OBIS | 749 | Caroppo, Pagliara, Azzaro, Miserocchi, and Azzaro (2017); Seuthe, Iversen, and Narcy (2011); GBIF; OBIS |

**References :**

Alvsvåg, J., Agnalt, A. L., & Jørstad, K. E. (2009). Evidence for a permanent establishment of the snow crab (*Chionoecetes opilio*) in the Barents Sea. *Biological Invasions, 11*(3), 587-595.

Andersen Borg, M. (2009). Non-indigenous zooplankton: the role of predatory cladocerans and of copepods in trophic dynamics.

Andrews, J. D. (1953). *Fouling organisms of Chesapeake Bay*. Chesapeake Bay Institute, Johns Hopkins University, Baltimore, Maryland. Interim Survey Program Interim Report XVII. 16 pp. + 6 pp appendix.

Anisimova, N., Berenboim, B., Gerasimova, O., Manushin, I., & Pinchukov, M. (2005). On the effect of red king crab on some components of the Barents Sea ecosystem. *Report PINRO, Murmansk*.

Armitage, C. S., Sjøtun, K., & Jensen, K. H. (2014). Correlative evidence for competition between *Fucus serratus* and the introduced chlorophyte *Codium fragile* subsp. *fragile* on the southwest coast of Norway. *Botanica Marina, 57*(2), 85-97.

Ben-Shlomo, R., Reem, E., Douek, J., & Rinkevich, B. (2010). Population genetics of the invasive ascidian *Botryllus schlosseri* from South American coasts. *Marine Ecology Progress Series, 412*, 85-92.

Berman, J., Harris, L., Lambert, W., Buttrick, M., & Dufresne, M. (1992). Recent invasions of the Gulf of Maine: three contrasting ecological histories. *Conservation Biology, 6*(3), 435-441.

Bollens, S. M., Cordell, J. R., Avent, S., & Hooff, R. (2002). Zooplankton invasions: a brief review, plus two case studies from the northeast Pacific Ocean. *Hydrobiologia, 480*(1-3), 87-110.

Boudreaux, M. L., Walters, L. J., & Rittschof, D. (2009). Interactions between native barnacles, non-native barnacles, and the Eastern Oyster *Crassostrea virginica*. *Bulletin of Marine Science, 84*(1), 43-57.

Brenchley, G. A., & Carlton, J. T. (1983). Competitive displacement of native mud snails by introduced periwinkles in the New England intertidal zone. *The Biological Bulletin, 165*(3), 543-558.

Buckland-Nicks, J., Chisholm, S. A., & Gibson, G. (2013). The living community inside the common periwinkle, *Littorina littorea*. *Canadian Journal of Zoology, 91*(5), 293-301.

Bulleri, F., & Airoldi, L. (2005). Artificial marine structures facilitate the spread of a non‐indigenous green alga, *Codium fragile* ssp. *tomentosoides*, in the north Adriatic Sea. *Journal of Applied Ecology, 42*(6), 1063-1072.

Calder, D. R. (1966). Ecology of marine invertebrate fouling organisms in Hampton Roads, Virginia. M.S. Thesis, College of William and Mary, Williamsburg, VA. vii + 66 pp.

Canning-Clode, J., Fofonoff, P. W., McCann, L., Carlton, J. T., & Ruiz, G. M. (2013). Marine invasions on a subtropical island: fouling studies and new records in a recent marina on Madeira Island (Eastern Atlantic Ocean).

Carignan, M. O., & Carreto, J. I. (2013). Characterization of mycosporine‐serine‐glycine methyl ester, a major mycosporine‐like amino acid from dinoflagellates: a mass spectrometry study. *Journal of Phycology, 49*(4), 680-688.

Carlton, J. T., & Cohen, A. N. (2003). Episodic global dispersal in shallow water marine organisms: the case history of the European shore crabs *Carcinus maenas* and *C. aestuarii*. *Journal of Biogeography, 30*(12), 1809-1820. doi:10.1111/j.1365-2699.2003.00962.x

Carlton, J. T., & Scanlon, J. A. (1985). Progression and dispersal of an introduced alga: *Codium fragile* ssp. *tomentosoides* (Chlorophyta) on the Atlantic coast of North America. *Botanica Marina, 28*(4), 155-166.

Caroppo, C., Pagliara, P., Azzaro, F., Miserocchi, S., & Azzaro, M. (2017). Late summer phytoplankton blooms in the changing polar environment of the Kongsfjorden (Svalbard, Arctic). *Cryptogamie, Algologie*.

Carver, C. E., Mallet, A. L., & Vercaemer, B. (2006a). *Biological synopsis of the colonial tunicates (Botryllus schlosseri and Botrylloides violaceus)* Canadian Manuscript Report of Fisheries and Aquatic Science 2747: v + 42 p.

Carver, C. E., Mallet, A. L., & Vercaemer, B. (2006b). *Biological synopsis of the solitary tunicate Ciona intestinalis.*Canadian Manuscript Report of Fisheries and Aquatic Science 2746: v + 55 p.

Chang, S.-J., Kim, J. N., Yoon, W.-D., & Ki, J.-S. (2016). First record of two cold-water jellyfishes *Aurelia limbata* and *Parumbrosa polylobata* (Scyphozoa: Semaeostomeae: Ulmaridae) in Korean coastal waters. *Animal Systematics, Evolution and Diversity, 32*(4), 272-280.

Chapman, A. S., Scheibling, R. E., & Chapman, A. R. O. (2002). Species introductions and changes in marine vegetation of Atlantic Canada. *Alien invaders in Canada’s waters, wetlands and forests. Natural Resources Canada, Canadian Forest Service Science Branch, Ottawa*, 133-148.

Chavanich, S., Harris, L. G., Je, J.-G., & Kang, R.-S. (2006). Distribution pattern of the green alga *Codium fragile* (Suringar) Hariot, 1889 in its native range, Korea. *Aquatic Invasions, 1*(3), 99-108.

Cheang, C. C., Chu, K. H., Fujita, D., Yoshida, G., Hiraoka, M., Critchley, A., . . . Ang Jr, P. O. (2010). Low genetic variability of *Sargassum muticum* (Phaeophyceae) revealed by a global analysis of native and introduced populations. *Journal of Phycology, 46*(6), 1063-1074.

Cohen, A. N., Carlton, J. T., & Fountain, M. C. (1995). Introduction, dispersal and potential impacts of the green crab *Carcinus maenas* in San Francisco Bay, California. *Marine Biology, 122*(2), 225-237.

Compton, T. J., Leathwick, J. R., & Inglis, G. J. (2010). Thermogeography predicts the potential global range of the invasive European green crab (*Carcinus maenas*). *Diversity and Distributions, 16*(2), 243-255. doi:10.1111/j.1472-4642.2010.00644.x

Crothers, J. H. (1968). The biology of the shore crab, *Carcinus maenas* (L.). 2. The life of the adult crab. *Field Stud., 2*, 579-614.

David, V., Sautour, B., & Chardy, P. (2007). Successful colonization of the calanoid copepod *Acartia tonsa* in the oligo-mesohaline area of the Gironde estuary (SW France)–Natural or anthropogenic forcing? *Estuarine, Coastal and Shelf Science, 71*(3-4), 429-442.

Dijkstra, J., & Harris, L. G. (2009). Maintenance of diversity altered by a shift in dominant species: implications for species coexistence. *Marine Ecology Progress Series, 387*, 71-80.

Dijkstra, J., Harris, L. G., & Westerman, E. (2007). Distribution and long-term temporal patterns of four invasive colonial ascidians in the Gulf of Maine. *Journal of Experimental Marine Biology and Ecology, 342*(1), 61-68.

Dijkstra, J., Sherman, H., & Harris, L. G. (2007). The role of colonial ascidians in altering biodiversity in marine fouling communities. *Journal of Experimental Marine Biology and Ecology, 342*(1), 169-171.

Drouin, A., McKindsey, C. W., & Johnson, L. E. (2012). Detecting the impacts of notorious invaders: experiments versus observations in the invasion of eelgrass meadows by the green seaweed *Codium fragile*. *Oecologia, 168*(2), 491-502.

Dunn, G. A. (1917). Development of *Dumontia filiformis*. II. Development of sexual plants and general discussion of results. *Botanical Gazette, 63*(6), 425-467.

Dvoretsky, A. G., & Dvoretsky, V. G. (2009). Fouling community of the red king crab, *Paralithodes camtschaticus* (Tilesius 1815), in a subarctic fjord of the Barents Sea. *Polar Biology, 32*(7), 1047-1054.

Eastwood, M. M., Donahue, M. J., & Fowler, A. E. (2007). Reconstructing past biological invasions: niche shifts in response to invasive predators and competitors. *Biological Invasions, 9*(4), 397-407.

El Atouani, S., Bentiss, F., Reani, A., Zrid, R., Belattmania, Z., Pereira, L., . . . Sabour, B. (2016). The invasive brown seaweed *Sargassum muticum* as new resource for alginate in Morocco: Spectroscopic and rheological characterization. *Phycological Research, 64*(3), 185-193.

Forrest, B. M., & Taylor, M. D. (2002). Assessing invasion impact: survey design considerations and implications for management of an invasive marine plant. *Biological Invasions, 4*(4), 375-386.

Gárate-Lizárraga, I., del Socorro Muñetón-Gómez, M., & Maldonado-López, V. (2011). Florecimiento del dinoflagelado *Gonyaulax polygramma* frente a la Isla Espiritu Santo, Golfo de California, México. *Revista de investigaciones marinas, 27*(1), 31-39.

Gaudy, R., & Viñas, M. D. (1985). Première signalisation en Méditerranée du copépode pélagique *Acartia tonsa*. *Rapp. Comm. Int. Mer Médit, 29*, 227-229.

GBIF *Acartia tonsa*: GBIF.org (3^rd^ May 2017) GBIF Occurrence Download <http://doi.org/10.15468/dl.sumqig>

GBIF *Alexandrium tamarense*: GBIF.org (12^th^ January 2018) GBIF Occurrence Download <https://doi.org/10.15468/dl.zauonm>

GBIF *Amphibalanus eburneus*: GBIF.org (27^th^ June 2017) GBIF Occurrence Download <http://doi.org/10.15468/dl.uuxcu1>

GBIF *Amphibalanus improvisus*: GBIF.org (10^th^ June 2014) GBIF Occurrence Download <http://api.gbif.org/v0.9/occurrence/download/request/0009129-140429114108248.zip>

GBIF *Aurelia limbata*: GBIF.org (2^nd^ May 2017) GBIF Occurrence Download <http://doi.org/10.15468/dl.s42l7g>

GBIF *Botryllus schlosseri*: GBIF.org (27^th^ June 2017) GBIF Occurrence Download <http://doi.org/10.15468/dl.0xnrga>

GBIF *Botrylloides violaceus*: GBIF.org (15^th^ December 2014) GBIF Occurrence Download <http://api.gbif.org/v1/occurrence/download/request/0005436-141123120432318.zip>

GBIF *Carcinus maenas*: GBIF.org (June 10^th^ 2014) GBIF Occurrence Download <http://api.gbif.org/v0.9/occurrence/download/request/0009134-140429114108248.zip>

GBIF *Ciona intestinalis*: GBIF.org (27^th^ June 2017) GBIF Occurrence Download <http://doi.org/10.15468/dl.wt6wnw>

GBIF *Codium fragile* subsp. *tomentosoides*: GBIF.org (3^rd^ May 2017) GBIF Occurrence Download <http://doi.org/10.15468/dl.biwzum>

GBIF *Dinophysis caudata*: GBIF.org (15^th^ January 2018) GBIF Occurrence Download <https://doi.org/10.15468/dl.x5z9gs>

GBIF *Dinophysis dens*: GBIF.org (15^th^ January 2018) GBIF Occurrence Download <https://doi.org/10.15468/dl.85xkqt>

GBIF *Dumontia contorta*: GBIF.org (23^rd^ November 2017) GBIF Occurrence Download <https://doi.org/10.15468/dl.rh4ycf>

GBIF *Gonyaulax polygramma*: GBIF.org (23^rd^ November 2017) GBIF Occurrence Download <https://doi.org/10.15468/dl.9y7acp>

GBIF *Kryptoperidinium triquetrum*: GBIF.org (15^th^ January 2018) GBIF Occurrence Download <https://doi.org/10.15468/dl.pclrq4>

GBIF *Membranipora membranacea*: GBIF.org (10^th^ June 2014) GBIF Occurrence Download <http://api.gbif.org/v0.9/occurrence/download/request/0009142-140429114108248.zip>

GBIF *Mnemiopsis leidyi*: GBIF.org (10^th^ April 2017) GBIF Occurrence Download <http://doi.org/10.15468/dl.ak6zel>

GBIF *Molgula manhattensis*: GBIF.org (27^th^ June 2017) GBIF Occurrence Download <http://doi.org/10.15468/dl.3708ta>

GBIF *Mya arenaria*: GBIF.org (10^th^ April 2017) GBIF Occurrence Download <http://doi.org/10.15468/dl.kai1wm>

GBIF *Littorina littorea*: GBIF.org (10^th^ June 2014) GBIF Occurrence Download <http://api.gbif.org/v0.9/occurrence/download/request/0009105-140429114108248.zip>

GBIF *Paralithodes camtschaticus*: GBIF.org (10^th^ June 2014) GBIF Occurrence Download http://api.gbif.org/v0.9/occurrence/download/request/0009151-140429114108248.zip

GBIF *Sargassum muticum*: GBIF.org (4^th^ May 2017) GBIF Occurrence Download <http://doi.org/10.15468/dl.e5wzt2>

GBIF *Undaria pinnatifida*: GBIF.org (4^th^ May 2017) GBIF Occurrence Download <http://doi.org/10.15468/dl.uiwfnr>

Gilbey, V., Attrill, M. J., & Coleman, R. A. (2008). Juvenile Chinese mitten crabs (*Eriocheir sinensis*) in the Thames estuary: distribution, movement and possible interactions with the native crab *Carcinus maenas*. *Biological Invasions, 10*(1), 67-77.

Grosholz, E. D., & Ruiz, G. M. (2002). Management plan for the European green crab: submitted to the Aquatic Nuisance Species Task Force.

Gubanova, A. (2000). Occurrence of Acartia tonsa Dana in the Black Sea. Was it introduced from the Mediterranean. *Mediterranean marine science, 1*(1), 105-109.

Hallegraeff, G. M. (1991). *Aquaculturists' guide to harmful Australian microalgae*: Fishing industry training board of Tasmania.

Hänfling, B., Edwards, F., & Gherardi, F. (2011). Invasive alien Crustacea: dispersal, establishment, impact and control. *BioControl, 56*(4), 573-595.

Hansen, H. S. B. (2015). *Snow crab (Chionoecetes opilio) in the Barents Sea. Diet, biology and management.* Master Thesis in International Fisheries Management, Norwegian College of Fishery Science, The Arctic University of Norway, Tromso. 95 pp.

Harris, L. G., & Jones, A. C. (2005). Temperature, herbivory and epibiont acquisition as factors controlling the distribution and ecological role of an invasive seaweed. *Biological Invasions, 7*(6), 913-924.

Harris, L. G., & Tyrrell, M. C. (2001). Changing community states in the Gulf of Maine: synergism between invaders, overfishing and climate change. *Biological Invasions, 3*(1), 9-21.

Haven, D. S., & Morales‐Alamo, R. (1966). Aspects of Biodeposition by Oysters and Other Invertebrate Filter Feeders1. *Limnology and Oceanography, 11*(4), 487-498.

Hemmingsen, W., Jansen, P. A., & MacKenzie, K. (2005). Crabs, leeches and trypanosomes: an unholy trinity? *Marine Pollution Bulletin, 50*(3), 336-339.

Huq, A., Sack, R. B., Nizam, A., Longini, I. M., Nair, G. B., Ali, A., . . . Yunus, M. (2005). Critical factors influencing the occurrence of *Vibrio cholerae* in the environment of Bangladesh. *Applied and Environmental Microbiology, 71*(8), 4645-4654.

Jonsson, P. R., & Tiselius, P. (1990). Feeding behaviour, prey detection and capture efficiency of the copepod *Acartia tonsa* feeding on planktonic ciliates. *Marine Ecology Progress Series*, 35-44.

Jørgensen, L. L. (2005). Impact scenario for an introduced decapod on Arctic epibenthic communities. *Biological Invasions, 7*(6), 949-957.

Jørgensen, L. L., & Nilssen, E. M. (2011). The invasive history, impact and management of the red king crab *Paralithodes camtschaticus* off the coast of Norway *In the Wrong Place-Alien Marine Crustaceans: Distribution, Biology and Impacts* (pp. 521-536): Springer.

Josselyn, M. N., & West, J. A. (1985). The distribution and temporal dynamics of the estuarine macroalgal community of San Francisco Bay. *Hydrobiologia, 129*(1), 139-152.

Kim, K.-Y., Kim, Y.-S., Hwang, C.-H., Lee, C.-K., Lim, W.-A., & Kim, C.-H. (2006). Phylogenetic analysis of dinoflagellate *Gonyaulax polygramma* Stein responsible for harmful algal blooms based on the partial LSU rDNA sequence data. *Algae, 21*(3), 283-286.

Koizumi, Y., Kohno, J., Matsuyama, N., Uchida, T., & Honjo, T. (1996). Environmental features and the mass mortality of fish and shellfish during the *Gonyaulax polygramma* red tide occurred in and around Uwajima Bay, Japan, in 1994. *Bulletin of the Japanese Society of Scientific Fisheries (Japan)*.

Korsun, S., Fahrni, J. F., & Pawlowski, J. (2012). Invading *Aurelia aurita* has established scyphistoma populations in the Caspian Sea. *Marine Biology, 159*(5), 1061-1069.

Kott, P. (2003). New syntheses and new species in the Australian Ascidiacea. *Journal of Natural History, 37*(13), 1611-1653.

Kozhenkova, S. I. (2009). Retrospective analysis of the marine flora of Vostok Bay, Sea of Japan. *Russian journal of marine biology, 35*(4), 263-278.

Kurashova, E. K., & Abdullayeva, N. M. (1984). *Acartia tonsa* (Calanoida, Acartiidae) in the Caspian Sea. *Zoology Journal, 63*(6).

Laget, F. (2017). *Transport d’espèces de dinoflagellés potentiellement non-indigènes dans l’Arctique canadien, suite au déversement des eaux de ballast par un navire domestique.* (Master thesis), Université du Québec à Rimouski, Rimouski.

Larsen, J. (1992). Potentially toxic phytoplankton. 2. Genus *Dinophysis* (Dinophyceae). *ICES identification leaflets for plankton*.

Larsen, J., & Moestrup, O. (1989). *Guide to toxic and potentially toxic marine algae*. Fish Inspection Service. Ministry of Fisheries.

Lee, J.-S., Igarashi, T., Fraga, S., Dahl, E., Hovgaard, P., & Yasumoto, T. (1989). Determination of diarrhetic shellfish toxins in various dinoflagellate species. *Journal of Applied Phycology, 1*(2), 147-152.

Leppäkoski, E., Olenin, S., & Gollasch, S. (2002). The Baltic Sea—a field laboratory for invasion biology. In: Leppäkoski E, Gollasch S, Olenin S (eds) *Invasive Aquatic Species of Europe. Distribution, Impacts and Management*. Kluwer Academic Publishers, The Netherlands (pp. 253-259)

Levin, P. S., Coyer, J. A., Petrik, R., & Good, T. P. (2002). Community‐wide effects of nonindigenous species on temperate rocky reefs. *Ecology, 83*(11), 3182-3193.

Lovvorn, J. (2010). Predicting snow crab growth and size with climate warming in the northern Bering Sea. *North Pacific Research Board Final Report, 713*, 24.

Lubchenco, J. (1983). *Littorina* and *Fucus*: effects of herbivores, substratum heterogeneity, and plant escapes during succession. *Ecology, 64*(5), 1116-1123.

Madariaga, D. J., Rivadeneira, M. M., Tala, F., & Thiel, M. (2014). Environmental tolerance of the two invasive species *Ciona intestinalis* and C*odium fragile*: their invasion potential along a temperate coast. *Biological Invasions, 16*(12), 2507-2527.

Malej, A., Tirelli, V., Lučić, D., Paliaga, P., Vodopivec, M., Goruppi, A., . . . Camatti, E. (2017). *Mnemiopsis leidyi* in the northern Adriatic: here to stay? *Journal of Sea Research, 124*, 10-16.

Martin, J. P., & Cuevas, J. M. (2006). First record of *Undaria pinnatifida* (Laminariales, Phaeophyta) in Southern Patagonia, Argentina. *Biological Invasions, 8*(6), 1399.

Mathieson, A. C., Hehre, E. J., Dawes, C. J., & Neefus, C. D. (2008). An historical comparison of seaweed populations from Casco Bay, Maine. *Rhodora, 110*(941), 1-103.

Mathieson, A. C., Moore, G. E., & Short, F. T. (2010). A floristic comparison of seaweeds from James Bay and three contiguous northeastern Canadian Arctic sites. *Rhodora, 112*(952), 396-434.

Mathieson, A. C., Pederson, J. R., Neefus, C. D., Dawes, C. J., & Bray, T. L. (2008). Multiple assessments of introduced seaweeds in the Northwest Atlantic. *ICES Journal of Marine Science: Journal du Conseil, 65*(5), 730-741.

Matsuno, K., Ichinomiya, M., Yamaguchi, A., Imai, I., & Kikuchi, T. (2014). Horizontal distribution of microprotist community structure in the western Arctic Ocean during late summer and early fall of 2010. *Polar Biology, 37*(8), 1185-1195.

McDonald, J. I., Huisman, J. M., Hart, F. N., Dixon, R. R. M., & Lewis, J. A. (2015). The first detection of the invasive macroalga *Codium fragile* subsp. *fragile* (Suringar) Hariot in Western Australia. *BioInvasions records, 4*(2), 75-80.

McKenzie, C. H., Matheson, K., Caines, S., & Wells, T. (2016). Surveys for non-indigenous tunicate species in Newfoundland, Canada (2006–2014): a first step towards understanding impact and control. *Biological Invasions, 7*(1), 21-32.

Mead, A., Carlton, J. T., Griffiths, C. L., & Rius, M. (2011). Introduced and cryptogenic marine and estuarine species of South Africa. *Journal of Natural History, 45*(39-40), 2463-2524.

Meretta, P. E., Matula, C. V., & Casas, G. (2012). Occurrence of the alien kelp *Undaria pinnatifida* (Laminariales, Phaeophyceae) in Mar del Plata, Argentina. *Bioinvasions Rec, 1*(1), 59-63.

Miyake, H., Lindsay, D. J., Hunt, J. C., & Hamatsu, T. (2002). Scyphomedusa *Aurelia limbata* (Brandt, 1838) found in deep waters off Kushiro, Hokkaido, Northern Japan. *Plankton Biology and Ecology, 49*(1), 44-46.

Molnar, J. L., Gamboa, R. L., Revenga, C., & Spalding, M. D. (2008). Assessing the global threat of invasive species to marine biodiversity. *Frontiers in Ecology and the Environment, 6*(9), 485-492.

Murphy, J. T., Johnson, M. P., & Viard, F. (2016). A modelling approach to explore the critical environmental parameters influencing the growth and establishment of the invasive seaweed *Undaria pinnatifida* in Europe. *Journal of Theoretical Biology, 396*, 105-115.

Neefus, C. D., Mathieson, A. C., Bray, T. L., & Yarish, C. (2008). The distribution, morphology, and ecology of three introduced Asiatic species of *Porphyra* (Bangiales, Rhodophyta) in the Northwestern Atlantic. *Journal of Phycology, 44*(6), 1399-1414.

Nicholson, N., Hosmer, H., Bird, K., Hart, L., Sandlin, W., Shoemaker, C., & Sloan, C. (1981, 1981). *The biology of Sargassum muticum (Yendo) Fensholt at Santa Catalina Island, California*.

Nyberg, C. D. (2007). *Introduced marine macroalgae and habitat modifiers: Their ecological role and significant attributes*. Department of Marine Ecology.

Obolewski, K., & Piesik, Z. (2005). *Mya arenaria* (L.) in the Polish Baltic Sea Coastal (Kołobrzeg-Władysławowo). *Baltic Coastal Zone, 9*, 13-27.

Okolodkov, Y. B. (2005). The global distributional patterns of toxic, bloom dinoflagellates recorded from the Eurasian Arctic. *Harmful Algae, 4*(2), 351-369.

Olesen, N. J. (1995). Clearance potential of jellyfish *Aurelia aurita*, and predation impact on zooplankton in a shallow cove. *Marine Ecology Progress Series, 124*, 63-72.

Otsuka, C. M., & Dauer, D. M. (1982). Fouling community dynamics in Lynnhaven Bay, Virginia. *Estuaries, 5*(1), 10-22.

Oug, E., Cochrane, S. K. J., Sundet, J. H., Norling, K., & Nilsson, H. C. (2011). Effects of the invasive red king crab (*Paralithodes camtschaticus*) on soft-bottom fauna in Varangerfjorden, northern Norway. *Marine Biodiversity, 41*(3), 467-479.

Pederson, J., Bullock, R., Carlton, J., Dijkstra, J., Dobroski, N., Dyrynda, P., Fisher, R., Harris, L., Hobbs, N., Lambert, G., Lazo-Wasem, E., Mathieson, A., Miglietta, M., Smith, J., Smith III, J., and Tyrrell, M. (2005). *Marine invaders in the Northeast: rapid assessment survey of non-native and native marine species of floating dock communitie*. MIT Sea Grant College Program Publication No. 05-3: iii + 40 pp.

Pereyra, P. J., Arias, M., González, R. A. C., & Narvarte, M. A. (2014). Moving forward: the Japanese kelp *Undaria pinnatifida* (Harvey) Suringar, 1873 expands in northern Patagonia, Argentina. *BioInvasions Records,* *3*(2), 65–70.

Pienimäki, M., & Leppäkoski, E. (2004). Invasion pressure on the Finnish Lake District: invasion corridors and barriers. *Biological Invasions, 6*(3), 331-346.

Primo, C., Hewitt, C. L., & Campbell, M. L. (2010). Reproductive phenology of the introduced kelp *Undaria pinnatifida* (Phaeophyceae, Laminariales) in Port Phillip bay (Victoria, Australia). *Biological Invasions, 12*(9), 3081-3092.

Provan, J. I. M., Murphy, S., & Maggs, C. A. (2005). Tracking the invasive history of the green alga *Codium fragile* ssp. *tomentosoides*. *Molecular Ecology, 14*(1), 189-194.

Purcell, J. E., & Arai, M. N. (2001). Interactions of pelagic cnidarians and ctenophores with fish: a review. *Hydrobiologia, 451*(1-3), 27-44.

Purcell, J. E., Uye, S.-i., & Lo, W.-T. (2007). Anthropogenic causes of jellyfish blooms and their direct consequences for humans: a review. *Marine Ecology Progress Series, 350*, 153-174.

Radchenko, K. V. (2013). New data on the distribution and feeding habits of jellyfish in the Northwest Pacific. *Russian Journal of Marine Biology, 39*(7), 509-520.

Reyes-Vasquez, G., Ferraz-Reyes, E., & Vasquez, E. (1979). Toxic dinoflagellate blooms in northeastern Venezuela during 1977. *Developments in marine biology, 1,* 191-194..

Rodil, I. F., Olabarria, C., Lastra, M., & López, J. (2008). Differential effects of native and invasive algal wrack on macrofaunal assemblages inhabiting exposed sandy beaches. *Journal of Experimental Marine Biology and Ecology, 358*(1), 1-13.

Ronowicz, M., Włodarska‐Kowalczuk, M., & Kukliński, P. (2013). Depth‐and substrate‐related patterns of species richness and distribution of hydroids (Cnidaria, Hydrozoa) in Arctic coastal waters (Svalbard). *Marine Ecology, 34*, 165-176.

Rosa, M., Holohan, B. A., Shumway, S. E., Bullard, S. G., Wikfors, G. H., Morton, S., & Getchis, T. (2013). Biofouling ascidians on aquaculture gear as potential vectors of harmful algal introductions. *Harmful Algae, 23*, 1-7.

Rzhavsky, A. V., Kuzmin, S. A., & Udalov, A. A. (2006). *State of the soft bottom communities in the Dalnezelenetskaya Bay after the red king crab introduction*.

Sabour, B., Reani, A., El Magouri, H., & Haroun, R. (2013). *Sargassum muticum* (Yendo) Fensholt (Fucales, Phaeophyta) in Morocco, an invasive marine species new to the Atlantic coast of Africa. *Aquatic Invasions, 8*(1), 97-102.

Saitsev, Y., & Ozturk, B. (2001). *Exotic species in the Aegean, Marmara, Black, Azov and Caspian Seas.* Turkish Marine Research Foundation (TUDAV).

Scheibling, R. E., & Gagnon, P. (2006). Competitive interactions between the invasive green alga *Codium fragile* ssp. *tomentosoides* and native canopy-forming seaweeds in Nova Scotia (Canada). *Marine Ecology Progress Series, 325*, 1-14.

Scheibling, R. E., & Gagnon, P. (2009). Temperature-mediated outbreak dynamics of the invasive bryozoan *Membranipora membranacea* in Nova Scotian kelp beds. *Marine Ecology Progress Series, 390*, 1-13.

Schiel, D. R., & Thompson, G. A. (2012). Demography and population biology of the invasive kelp *Undaria pinnatifida* on shallow reefs in southern New Zealand. *Journal of Experimental Marine Biology and Ecology, 434*, 25-33.

Schmidt, A. L., & Scheibling, R. E. (2006). A comparison of epifauna and epiphytes on native kelps (*Laminaria* species) and an invasive alga (*Codium fragile* ssp. *tomentosoides*) in Nova Scotia, Canada. *Botanica Marina, 49*(4), 315-330.

Selander, E., Møller, L. F., Sundberg, P., & Tiselius, P. (2010). Parasitic anemone infects the invasive ctenophore *Mnemiopsis leidyi* in the North East Atlantic. *Biological Invasions, 12*(5), 1003-1009.

Seuthe, L., Iversen, K. R., & Narcy, F. (2011). Microbial processes in a high-latitude fjord (Kongsfjorden, Svalbard): II. Ciliates and dinoflagellates. *Polar Biology, 34*(5), 751-766.

Sfriso, A., & Facca, C. (2013). Annual growth and environmental relationships of the invasive species *Sargassum muticum* and *Undaria pinnatifida* in the lagoon of Venice. *Estuarine, Coastal and Shelf Science, 129*, 162-172.

Shiganova, T. A. (1998). Invasion of the Black Sea by the ctenophore *Mnemiopsis leidyi* and recent changes in pelagic community structure. *Fisheries Oceanography, 7*(3‐4), 305-310.

Shiganova, T. A. (2004). Some results of studying the intruder *Mnemiopsis leidyi* (A. Agasssiz) in the Black Sea. *Ctenophore Mnemiopsis leidyi (A. Agasssiz) in the Azov and Black Seas: Its Biology and Consequences of Its Intrusion*, 28-68.

Simkanin, C., Fofonoff, P. W., Larson, K., Lambert, G., Dijkstra, J., & Ruiz, G. M. (2016). Spatial and temporal dynamics of ascidian invasions in the continental United States and Alaska. *Marine Biology, 163*(7), 163.

Skolka, M., & Preda, C. (2010). Alien invasive species at the Romanian Black Sea coast-present and perspectives. *Travaux du Muséum National d'Histoire Naturelle" Grigore Antipa", 53*(1), 443-467.

Sorte, C. J. B., Williams, S. L., & Zerebecki, R. A. (2010). Ocean warming increases threat of invasive species in a marine fouling community. *Ecology, 91*(8), 2198-2204.

Steidinger, K. A., Tangen, K., & Tomas, C. R. (1996). Identifying marine diatoms and dinoflagellates. *Academic Press, Inc., San Diego*.

Stewart, D. B. & Howland, K. L. (2009)**.** *An ecological and oceanographical assessment of the alternate ballast water exchange zone in the Hudson Strait region***.** DFO Canadian Science Advisory Secretariat Research Document 2009/008. vi + 96 p.

Strasser, M. (1998). *Mya arenaria*—an ancient invader of the North Sea coast. *Helgoländer Meeresuntersuchungen, 52*(3-4), 309-324.

Tamiyavanich, S., Kodama, M., & Fukuyo, Y. (1985, 1985). *The occurrence of paralytic shellfish poisoning in Thailand*.

Teegarden, G. J., & Cembella, A. D. (1996). Grazing of toxic dinoflagellates, *Alexandrium* spp., by adult copepods of coastal Maine: implications for the fate of paralytic shellfish toxins in marine food webs. *Journal of Experimental Marine Biology and Ecology, 196*(1-2), 145-176.

Telesh, I. (2008). *Zooplankton of the open Baltic Sea: atlas*: Leibniz-Institut für Ostseeforschung.

Therriault, T. W. & Herborg, L-M. (2008) *Risk assessment for two solitary and three colonial tunicates in both Atlantic and Pacific Canadian waters.* Canadian Science Advisory Secretariat Research Document 2007/063: iv + 64 p.

Trowbridge, C. D. (1999). *An assessment of the potential spread and options for control of the introduced green macroalga Codium fragile ssp. tomentosoides on Australian shores.* Centre for Research on Introduced Marine Pests.

Tulp, A. S. (2006). *Mnemiopsis leidyi* (Agassiz, 1865) (Ctenophora, Lobata) in de Waddenzee. *Het Zeepaard, 66*(6), 183-189.

Turon, X., Cañete, J. I., Sellanes, J., Rocha, R. M., & López-Legentil, S. (2016). Too cold for invasions? Contrasting patterns of native and introduced ascidians in subantarctic and temperate Chile. *Management of Biological Invasions,* *7*(1), 77-86.

Vaz-Pinto, F., Torrontegi, O., Prestes, A. C. L., Álvaro, N. V., Neto, A. I., & Martins, G. M. (2014). Invasion success and development of benthic assemblages: Effect of timing, duration of submersion and substrate type. *Marine Environmental Research, 94*, 72-79.

Veldhuizen, T.C. & Stanish, S. (1999). *Overview of the life history, distribution, abundance, and impact of the Chinese mitten crab,* *Eriocheir sinensis*. California Department of Water Resources. Environmental Services Office. 6 pp.

Wallentinus, I., & Nyberg, C. D. (2007). Introduced marine organisms as habitat modifiers. *Marine Pollution Bulletin, 55*(7-9), 323-332.

White, L. L. F. (2010). Mechanisms underlying marine macroalgal invasions: understanding invasion success of *Sargassum muticum*.

Wieczorek, S. K., & Hooper, R. G. (1995). Relationship between diet and food availability in the snow crab *Chionoecetes opilio* (O. Fabricius) in Bonne Bay, Newfoundland. *Journal of Crustacean Biology, 15*(2), 236-247.

Włodarska-Kowalczuk, M., Kukliński, P., Ronowicz, M., Legeżyńska, J., & Gromisz, S. (2009). Assessing species richness of macrofauna associated with macroalgae in Arctic kelp forests (Hornsund, Svalbard). *Polar Biology, 32*(6), 897-905.

Wolff, W. J. (2005) Non-indigenous marine and estuarine species in The Netherlands. *Zoologische Mededelingen* 79, 1-116.

Wong, M. C., & Vercaemer, B. (2012). Effects of invasive colonial tunicates and a native sponge on the growth, survival, and light attenuation of eelgrass (*Zostera marina*). *Aquatic Invasions, 7*(3).

Yamada, S. B., & Mansour, R. A. (1987). Growth inhibition of native *Littorina saxatilis* (Olivi) by introduced *L. littorea* (L.). *Journal of Experimental Marine Biology and Ecology, 105*(2-3), 187-196.

Yasumoto, T. (1990). Marine microorganisms toxins-- an overview. In: Graneli E, Sundstrom B, Edler L, Anderson DM (eds) *Toxic marine phytoplankton*. Elsevier Science Publications, New York, pp 3–8

Zavolokin, A. V. (2010). Distribution and abundance dynamics of jellyfish in the Sea of Okhotsk. *Russian Journal of Marine Biology, 36*(3), 157-166.
