# Supplementary material for: What and where? Predicting invasion hotspots in the Arctic marine realm
Source: Glob Chang Biol. 2020 Jul 10;26(9):4752–71. doi: 10.1111/gcb.15159 (PMC7496761; doi:10.1111/gcb.15159)
Supplement: Supplementary file 5 — Table S2 [file GCB-26-4752-s005.docx]

**Table S2**: Complete suite of environmental variables gathered for its use in species distribution modelling. Correlation between variables (coefficients ≥ 0.7) are also shown. Modified from Tyberghein et al. (2012) and Assis et al. (2018).

| **Variable** | **Units** | **Range** | **Type of values used** | **Source** | **Resolution** | **Correlated to** |
| --- | --- | --- | --- | --- | --- | --- |
| Temperature | °C | -1.94; 39.22 | Bottom and Sea Surface: minimum, maximum, mean | ARMOR  (Bio-ORACLE v2) | 5 arcmin | -Bottom T (min, max, mean) between them (>0.95)  -SST (min, max, mean) between them (>0.96)  -SST (min, max, mean) with Dissolved O_2_ (min, max, mean) (>-0.93)  -SST max with Phosphate (max, min) (>-0.71)  -SST mean with Phosphate max (>-0.7)  -SST (mean and min) with chlorophyll max (>-0.72) |
| Salinity | PSS | 4.75; 41.96 | Bottom and Sea Surface: minimum, maximum, mean | ARMOR  (Bio-ORACLE v2) | 5 arcmin | -Bottom salinity (min, max, mean) between them (>0.9)  -SSS (min, max, mean) between them (>0.92)  -SSS (max, mean) with bottom salinity (min, max, mean) (>0.71)  -SSS min with bottom salinity min (>0.7) |
| Sea ice thickness | m | 0; 10.94 | Minimum, maximum, mean | ORAP  (Bio-ORACLE v2) | 5 arcmin | -Sea ice (min, max, mean) between them (>0.9) |
| Chlorophyll | mg/m^3^ | 0; 17.46 | Minimum, maximum, mean | PISCES  (Bio-ORACLE v2) | 5 arcmin | -Chlorophyll mean with Chlorophyll max (>0.84)  - Chlorophyll max with Dissolved O_2_ (min, max, mean) (>0.72)  --Chlorophyll max with SST (mean and min) (>-0.72) |
| Dissolved molecular oxygen | µmol/m^3^ | 0; 789.94 | Minimum, maximum, mean | PISCES  (Bio-ORACLE v2) | 5 arcmin | -Dissolved O_2_ (min, max, mean) between them (>0.96)  - Dissolved O_2_ (min, max, mean) with SST (min, max, mean) (>-0.93)  - Dissolved O_2_ (min, max, mean) with chlorophyll max (>0.72) |
| pH | - | 6.68; 8.63 | Mean | WOD 2009  (Bio-ORACLE v2) | 5 arcmin | - |
| Photosynthe-tically active radiation (PAR) | Einstein  /m²/day | 0.44; 68.1 | Maximum, mean | SeaWiFS  (Bio-ORACLE v2) | 5 arcmin | - |
| Nitrate | µmol/m^3^ | 0; 164.51 | Minimum, maximum, mean | PISCES  (Bio-ORACLE v2) | 5 arcmin | -Nitrate (min, max, mean) between them (>0.96)  -Nitrate (min, max, mean) with phosphate (min, max, mean) (>0.94)  -Nitrate (min, max, mean) with silicate (min, max, mean) (>0.82) |
| Phosphate | µmol/m^3^ | 0; 3.55 | Minimum, maximum, mean | PISCES  (Bio-ORACLE v2) | 5 arcmin | -Phosphate (min, max, mean) between them (>0.98)  -Phosphate (min, max, mean) with silicate (min, max, mean) (>0.79)  - Phosphate (max, min) with SST max (>-0.71)  - Phosphate max with SST mean (>-0.7)  - Phosphate (min, max, mean) with nitrate (min, max, mean) ) (>0.94) |
| Silicate | µmol/m^3^ | 0.46; 316.67 | Minimum, maximum, mean | PISCES  (Bio-ORACLE v2) | 5 arcmin | -Silicate (min, max, mean) between them (>0.98)  - Silicate (min, max, mean) with nitrate (min, max, mean) (>0.82)  - Silicate (min, max, mean) with phosphate (min, max, mean) (>0.79) |
| Calcite | mol/m^3^ | 0; 0.06 | Mean | Aqua-MODIS  (Bio-ORACLE v2) | 5 arcmin | - |
| Dissolved iron | µmol/m^3^ | 0; 0.03 | Minimum, maximum, mean | PISCES  (Bio-ORACLE v2) | 5 arcmin | Iron (min, max, mean) between them (>0.92) |
| Depth | m | 0; 8405.46 | - | ETOPO 2  (Aquamaps) | 30 arcmin | - |
| Land distance | m | 0; 2708.67 | - | SAUP  (Aquamaps) | 30 arcmin | - |

ARMOR: Global Observed Ocean Physics Reprocessing; ORAP: Global Ocean Physics Reanalysis ECMWF; PISCES: Global Ocean Biogeochemistry Non-assimilative Hindcast; WOD: World Ocean Database, Aqua-MODIS: Moderate Resolution Imaging Spectroradiometer; SeaWiFS: OceanColor Biology Processing Group

**References:**

Assis, J., Tyberghein, L., Bosch, S., Verbruggen, H., Serrão, E. A., & De Clerck, O. (2018). Bio‐ORACLE v2. 0: Extending marine data layers for bioclimatic modelling. *Global Ecology and Biogeography, 27*(3), 277-284.

Tyberghein, L., Verbruggen, H., Pauly, K., Troupin, C., Mineur, F., & De Clerck, O. (2012). Bio‐ORACLE: a global environmental dataset for marine species distribution modelling. *Global Ecology and Biogeography, 21*(2), 272-281.
