# Supplementary material for: What and where? Predicting invasion hotspots in the Arctic marine realm
Source: Glob Chang Biol. 2020 Jul 10;26(9):4752–71. doi: 10.1111/gcb.15159 (PMC7496761; doi:10.1111/gcb.15159)
Supplement: Supplementary file 6 — Table S3 [file GCB-26-4752-s006.docx]

**Table S3**: Environmental variables used for each species modelled. ZB: zoobenthos; PB: phytobenthos; ZP: zooplankton; PP: phytoplankton; Clas: classification of ecological group; BT: bottom temperature; SST: sea surface temperature; BS: bottom salinity; SSS: sea surface salinity. Cells marked in grey correspond to variables that were not considered for that particular taxa group.

| **Species** | **Taxa** | **Clas** | **Environmental variables used** | | | | | | | |
| --- | --- | --- | --- | --- | --- | --- | --- | --- | --- | --- |
|  |  |  | **Temperature** | **Salinity** | **Ice** | **Depth** | **Land distance** | **pH** | **DO_2_** | **Nutrients/**  **minerals** |
| *Amphibalanus eburneus* | Crustacea | ZB | X BT max SST min |  |  |  | X |  |  |  |
| *Botrylloides violaceus* | Tunicata | ZB | X BT max SST mean |  |  |  | X |  |  |  |
| *Botryllus schlosseri* | Tunicata | ZB | X BT max | X SSS min |  | X |  |  |  |  |
| *Carcinus maenas* | Crustacea | ZB | X BT max SST mean |  |  | X |  |  |  |  |
| *Chionoecetes opilio* | Crustacea | ZB | X SST mean | X SSS max | X mean | X |  |  |  |  |
| *Ciona intestinalis* | Tunicata | ZB | X BT max | X SSS min |  |  | X |  |  |  |
| *Littorina littorea* | Mollusca | ZB | X BT max SST max | X SSS min |  | X |  |  |  |  |
| *Membranipora membranacea* | Bryozoa | ZB | X BT max |  |  | X | X |  |  |  |
| *Molgula manhattensis* | Tunicata | ZB | X BT max | X BS min |  | X | X |  |  |  |
| *Mya arenaria* | Mollusca | ZB | X BT max | X BS min |  | X |  |  |  |  |
| *Paralithodes camtschaticus* | Crustacea | ZB | X BT max SST mean |  |  | X |  |  |  |  |
| *Codium fragile* spp. *fragile* | Chlorophyta | PB | X BT max SST mean |  | X  mean |  | X |  |  |  |
| *Dumontia contorta* | Rhodophyta | PB | X BT max |  |  | X | X |  |  | X Iron min |
| *Sargassum muticum* | Phaeophycea | PB | X BT mean |  |  |  | X |  |  | X Nitrate max |
| *Undaria pinnatifida* | Phaeophycea | PB | X BT max |  | X mean | X | X |  | X max | X Calcite |
| *Acartia (Acanthacartia) tonsa* | Copepoda | ZP | X SST mean | X SSS min |  | X | X |  |  |  |
| *Aurelia limbata* | Cnidaria | ZP | X SST mean |  | X mean | X |  | X |  |  |
| *Mnemiopsis leidyi* | Ctenophora | ZP | X SST max | X SSS min | X mean |  |  |  |  |  |
| *Alexandrium tamarense* | Dinoflagellata | PP | X SST mean | X SSS min |  |  | X |  |  | X Iron min Nitrate mean |
| *Dinophysis caudata* | Dinoflagellata | PP | X SST mean |  |  |  | X | X |  | X Iron max Phosphate mean |
| *Dinophysis dens* | Dinoflagellata | PP | X SST max |  |  |  | X |  |  | X Iron min Nitrate max |
| *Gonyaulax polygramma* | Dinoflagellata | PP | X SST mean |  |  |  | X | X |  | X Iron mean Silicate mean |
| *Kryptoperidinium triquetrum* | Dinoflagellata | PP | X SST mean | X SSS max |  |  | X |  |  | X Iron min |

**Note:** Chlorophyll was taken into account for zooplankton and PAR for phytobenthos and phytoplankton when they were first modelled; but they do not appear in the table given that they were not selected as having >4% contribution to the model as explained in the main text.
