# Supplementary material for: What and where? Predicting invasion hotspots in the Arctic marine realm
Source: Glob Chang Biol. 2020 Jul 10;26(9):4752–71. doi: 10.1111/gcb.15159 (PMC7496761; doi:10.1111/gcb.15159)
Supplement: Supplementary file 7 — Table S4 [file GCB-26-4752-s007.docx]

**Table S4**: Depth thresholds used to reflect ecological requirements of AIS models.

| **Species** | **Known max depth (m)** | **Reference** | **Mask for maximum depth used (m)** |
| --- | --- | --- | --- |
| ***Amphibalanus eburneus*** | 37 | Voss (1976) | 50 |
| ***Botrylloides violaceus*** | <50 | Therriault and Herborg (2008b) | 50 |
| ***Botryllus schlosseri*** | 200 | Carver, Mallet, and Vercaemer (2006); NEMESIS | 200 |
| ***Carcinus maenas*** | 55 | Crothers (1968); Grosholz and Ruiz (2002); Klassen and Locke (2007) | 50 |
| ***Chionoecetes opilio*** | 1400 | Dawe and Colbourne (2002) | 1500 |
| ***Ciona intestinalis*** | 200-500 | Therriault and Herborg (2008a) | 500 |
| ***Littorina littorea*** | Sublittoral | Carlson, Shulman, and Ellis (2006); NEMESIS; CABI | 50 |
| ***Membranipora membranacea*** | 12 | Saunders and Metaxas (2007); Saunders and Metaxas (2009) | 50 |
| ***Molgula manhattensis*** | 90 | Lambert and Lambert (1998); Lambert and Lambert (2003); Zvyagintsev and Ke (2003); http://wdfw.wa.gov/ais/molgula_manhattensis/ | 100 |
| ***Mya arenaria*** | 100 | NEMESIS | 100 |
| ***Paralithodes camtschaticus*** | 400 | Jewett and Onuf (1988) | 500 |
| ***Codium fragile* spp. *fragile*** | Intertidal / subtidal | Mathieson, Hehre, Dawes, and Neefus (2008) | 50 |
| ***Dumontia contorta*** | Intertidal / subtidal | Algaebase | 50 |
| ***Sargassum muticum*** | Intertidal / subtidal | Druehl (2000) | 50 |
| ***Undaria pinnatifida*** | Intertidal / subtidal | CABI | 50 |
| ***Acartia (Acanthacartia) tonsa*** | 26 | Chen and Hare (2008); NOBANIS | 50 |
| ***Aurelia limbata*** | 200-250 | Miyake, Lindsay, Hunt, and Hamatsu (2002) | 200 |
| ***Mnemiopsis leidyi*** | 80-110 | Huwer, Storr-Paulsen, Riisgard, and Haslob (2008) | 100 |

**Note:** known depths were grouped to the closest mask values for round up. For intertidal species such as phytobenthos, a mask of 50 m depth was used as the minimum given that data at the coastal regions can be affected by accuracy errors.

**References:**

Carlson, R. L., Shulman, M. J., & Ellis, J. C. (2006). Factors contributing to spatial heterogeneity in the abundance of the common periwinkle *Littorina littorea* (L.). *Journal of Molluscan Studies, 72*(2), 149-156.

Carver, C. E., Mallet, A. L., & Vercaemer, B. (2006). *Biological synopsis of the colonial tunicates (Botryllus schlosseri and Botrylloides violaceus):* Canadian Manuscript Report of Fisheries and Aquatic Sciences 2747. Retrieved from: http://publications.gc.ca/site/eng/314382/publication.html

Chen, G., & Hare, M. P. (2008). Cryptic ecological diversification of a planktonic estuarine copepod, *Acartia tonsa*. *Molecular Ecology, 17*(6), 1451-1468.

Crothers, J. H. (1968). The biology of the shore crab, *Carcinus maenas* (L.). The life of the adult crab. *Field Stud., 2*, 579-614.

Dawe, E. G., & Colbourne, E. B. (2002). *Distribution and demography of snow crab (Chionoecetes opilio) males on the Newfoundland and Labrador Shelf.* Crabs in cold water regions: biology, management, and economics. Alaska Sea Grant Coll Program, AK-SG-02-01, Fairbanks, AK, 577-594.

Druehl, L. D. (2000). *Pacific Seaweeds: A guide to common seaweeds of the West Coast.* Harbour Publishing Company.

Grosholz, E. D., & Ruiz, G. M. (2002). *Management plan for the European green crab*. Submitted to the Aquatic Nuisance Species Task Force. Retrieved from: https://www.anstaskforce.gov/GreenCrabManagementPlan.pdf

Huwer, B., Storr-Paulsen, M., Riisgard, H. U., & Haslob, H. (2008). Abundance, horizontal and vertical distribution of the invasive ctenophore *Mnemiopsis leidyi* in the central Baltic Sea, November 2007. *Aquatic Invasions, 3*(2), 113-124.

Jewett, S. C., & Onuf, C. P. (1988). *Habitat suitability index models: red king crab*. Report U.S. Fish and Wildlife Service 82/10.153, pp. 34. Retrieved from: https://pubs.er.usgs.gov/publication/fwsobs82_10_153

Klassen, G. J., & Locke, A. (2007). *A biological synopsis of the European green crab, Carcinus maenas.* Canadian Manuscript Report of Fisheries and Aquatic Sciences 2818, pp. 75. Retrieved from: http://www.dfo-mpo.gc.ca/library/330845.pdf

Lambert, C. C., & Lambert, G. (1998). Non-indigenous ascidians in southern California harbors and marinas. *Marine Biology, 130*(4), 675-688.

Lambert, C. C., & Lambert, G. (2003). Persistence and differential distribution of nonindigenous ascidians in harbors of the Southern California Bight. *Marine Ecology Progress Series, 259*, 145-161.

Mathieson, A. C., Hehre, E. J., Dawes, C. J., & Neefus, C. D. (2008). An historical comparison of seaweed populations from Casco Bay, Maine. *Rhodora, 110*(941), 1-103.

Miyake, H., Lindsay, D. J., Hunt, J. C., & Hamatsu, T. (2002). Scyphomedusa *Aurelia limbata* (Brandt, 1838) found in deep waters off Kushiro, Hokkaido, Northern Japan. *Plankton Biology and Ecology, 49*(1), 44-46.

Saunders, M., & Metaxas, A. (2007). Temperature explains settlement patterns of the introduced bryozoan *Membranipora membranacea* in Nova Scotia, Canada. *Marine Ecology Progress Series, 344*, 95-106.

Saunders, M., & Metaxas, A. (2009). Population dynamics of a nonindigenous epiphytic bryozoan *Membranipora membranacea* in the western North Atlantic: effects of kelp substrate. *Aquatic Biology, 8*(1), 83-94.

Therriault, T. W., & Herborg, L.-M. (2008a). A qualitative biological risk assessment for vase tunicate *Ciona intestinalis* in Canadian waters: using expert knowledge. *ICES Journal of Marine Science, 65*(5), 781-787.

Therriault, T. W., & Herborg, L.-M. (2008b). *Risk assessment for two solitary and three colonial tunicates in both Atlantic and Pacific Canadian waters.* Canadian Science Advisory Secretariat 2007/063. Retrieved from: http://www.dfo-mpo.gc.ca/csas-sccs/publications/resdocs-docrech/2007/2007_063-eng.htm

Voss, G. L. (1976). *Seashore life of Florida and the Caribbean*. New York: Dover Publications, 199 pp.

Zvyagintsev, A. Y., & Ke, S. (2003). The introduction of the ascidian *Molgula manhattensis* (De Kay, 1843) into Peter the Great Bay (Sea of Japan). *Sessile organisms, 20*(1), 7-10.
