# Supplementary material for: What and where? Predicting invasion hotspots in the Arctic marine realm
Source: Glob Chang Biol. 2020 Jul 10;26(9):4752–71. doi: 10.1111/gcb.15159 (PMC7496761; doi:10.1111/gcb.15159)
Supplement: Supplementary file 8 — Table S5 [file GCB-26-4752-s008.docx]

**Table S5**: Model evaluation indicators: Area Under the Curve (AUC) , sensitivity, specificity, and True Skill Statistic (TSS) ± Standard Deviation (in parenthesis after each evaluation statistic value is shown) obtained for each species and each model performed. The number of asterisks (*) next to the model evaluation indicators defines the number of replicates that were excluded from the final average ensemble model due to the cut-off for excluding models with TSS scores < 0.7 and sensitivity = 0.

| Species |  | ANN | GAM | GLM | RF | MaxEnt |
| --- | --- | --- | --- | --- | --- | --- |
| *Amphibalanus eburneus* | AUC | 0.994  (0.002) | 0.995  (0.002) | 0.991  (0.001) | 0.996  (0.002) | 0.988 (0.003) |
|  | Sensitivity | 0.972  (0.026) | 0.987  (0.008) | 0.981  (0.017) | 0.991  (0.008) | 0.952 (0.016) |
|  | Specificity | 0.978  (0.008) | 0.976  (0.009) | 0.965  (0.024) | 0.981  (0.003) | 0.968 (0.002) |
|  | TSS | 0.951  (0.023) | 0.963  (0.009) | 0.946  (0.021) | 0.973  (0.005) | 0.920 (0.014) |
| *Botrylloides violaceus* | AUC | 0.998  (0.001) | 0.998  (0.001) | 0.997  (0.001) | 0.999  (0.001) | 0.982 (0.001) |
|  | Sensitivity | 0.992  (0.003) | 0.994  (0.003) | 0.994  (0.003) | 0.995  (0.002) | 0.991 (0.009) |
|  | Specificity | 0.991  (0.003) | 0.99  (0.002) | 0.988  (0.003) | 0.994  (0.001) | 0.983 (0.003) |
|  | TSS | 0.984  (0.002) | 0.985  (0.003) | 0.982  (0.002) | 0.989  (0.002) | 0.973 (0.007) |
| *Botryllus schlosseri* | AUC | 0.996  (0.001) | 0.996  (0.001) | 0.994  (0.001) | 0.999  (0.001) | 0.918 (0.004) |
|  | Sensitivity | 0.993  (0.003) | 0.993  (0.002) | 0.99  (0.003) | 0.994  (0.001) | 0.992 (0.005) |
|  | Specificity | 0.971  (0.003) | 0.969  (0.001) | 0.97  (0.004) | 0.99  (0.001) | 0.953 (0.001) |
|  | TSS | 0.964  (0.001) | 0.962  (0.002) | 0.961  (0.001) | 0.984  (0.001) | 0.945 (0.005) |
| *Carcinus maenas* | AUC | 0.997  (0.001) | 0.998  (0.001) | 0.998  (0.001) | 0.999  (0.001) | 0.911 (0.002) |
|  | Sensitivity | 0.994  (0.001) | 0.993  (0.001) | 0.994  (0.001) | 0.995  (0.001) | 0.973 (0.002) |
|  | Specificity | 0.992  (0.001) | 0.992  (0.002) | 0.992  (0.001) | 0.993  (0.001) | 0.973 (0.003) |
|  | TSS | 0.986  (0.001) | 0.985  (0.001) | 0.985  (0.001) | 0.988  (0.001) | 0.946 (0.003) |
| *Chionoecetes opilio* | AUC | 0.993  (0.001) | 0.996  (0.001) | 0.994  (0.001) | 0.998  (0.001) | 0.757 (0.005) |
|  | Sensitivity | 0.992  (0.001) | 0.991  (0.001) | 0.99  (0.001) | 0.996  (0.002) | 0.991 (0.002) |
|  | Specificity | 0.984  (0.003) | 0.983  (0.001) | 0.983  (0.002) | 0.993  (0.002) | 0.931 (0.007) |
|  | TSS | 0.976  (0.003) | 0.974  (0.002) | 0.974  (0.002) | 0.988  (0.001) | 0.922 ± 0.006 |
| *Ciona intestinalis* | AUC | 0.996  (0.001) | 0.998  (0) | 0.997  (0.001) | 0.999  (0.001) | 0.953 (0.003) |
|  | Sensitivity | 0.995  (0.001) | 0.993  (0.003) | 0.994  (0.002) | 00.997  (0.001) | 0.99  (0.002) |
|  | Specificity | 0.984  (0.002) | 0.986  (0.004) | 0.981  (0.002) | 0.99  (0.001) | 0.975 (0.003) |
|  | TSS | 0.98  (0.002) | 0.979  (0.001) | 0.999  (0.001) | 0.987  (0.002) | 0.966 ± 0.004 |
| *Littorina littorea* | AUC | 0.994  (0.001) | 0.997  (0.001) | 0.996  (0.001) | 0.999  (0) | 0.919 (0.004) |
|  | Sensitivity | 0.993  (0.002) | 0.995  (0.003) | 0.99  (0.002) | 0.998  (0.001) | 0.986 (0.002) |
|  | Specificity | 0.984  (0.002) | 0.982  (0.003) | 0.985  (0.001) | 0.993  (0.001) | 0.962 (0.010) |
|  | TSS | 0.978  (0.001) | 0.977  (0.001) | 0.976  (0.002) | 0.99  (0.001) | 0.948 (0.008) |
| *Membranipora membranacea* | AUC | 0.997  (0.001) | 0.997  (0.001) | 0.997  (0) | 0.998  (0.001) | 0.939 (0.001) |
|  | Sensitivity | 0.994  (0.001) | 0.994  (0.001) | 0.993  (0.001) | 0.997  (0.001) | 0.989 (0.001) |
|  | Specificity | 0.99  (0.001) | 0.989  (0.001) | 0.99  (0.001) | 0.991  (0.002) | 0.972 (0.005) |
|  | TSS | 0.984  (0.002) | 0.983  (0.002) | 0.983  (0.001) | 0.987  (0.002) | 0.961 ± 0.005 |
| *Molgula manhattensis* | AUC | 0.987  (0.006) | 0.997  (0.001) | 0.987  (0.001) | 0.999  (0.001) | 0.973 (0.003) |
|  | Sensitivity | 0.978  (0.012) | 0.993  (0.001) | 0.977  (0.006) | 0.992  (0.001) | 0.991 (0.004) |
|  | Specificity | 0.978  (0.006) | 0.983  (0.002) | 0.954  (0.002) | 0.992  (0.002) | 0.959 (0.005) |
|  | TSS | 0.957  (0.01) | 0.976  (0.002) | 0.931  (0.004) | 0.984  (0.002) | 0.950 (0.003) |
| *Mya arenaria* | AUC | 0.997  (0.001) | 0.997  (0.001) | 0.997  (0.001) | 0.999  (0.001) | 0.908 (0.005) |
|  | Sensitivity | 0.993  (0.003) | 0.987  (0.002) | 0.989  (0.004) | 0.994  (0.002) | 0.984 (0.001) |
|  | Specificity | 0.981  (0.003) | 0.984  (0.002) | 0.978  (0.006) | 0.989  (0.002) | 0.959 (0.001) |
|  | TSS | 0.974  (0.003) | 0.971  (0.002) | 0.967  (0.003) | 0.983  (0.002) | 0.942 (0.001) |
| *Paralithodes camtschaticus* | AUC | 0.997  (0.001) | 0.996  (0.001) | 0.995  (0.001) | 0.998  (0.001) | 0.964 (0.001) |
|  | Sensitivity | 0.992  (0.004) | 0.991  (0.002) | 0.993  (0.002) | 0.992  (0.003) | 0.979 (0.025) |
|  | Specificity | 0.98  (0.005) | 0.977  (0.002) | 0.974  (0.001) | 0.981  (0.006) | 0.967  (0.01) |
|  | TSS | 0.972  (0.003) | 0.968  (0.002) | 0.974  (0.001) | 0.974  (0.004) | 0.946 (0.015) |
| *Codium fragile* spp. *fragile* | AUC | 0.996  (0.001) | 0.996  (0) | 0.994  (0.003) | 0.996  (0.004) | 0.992 (0.002) |
|  | Sensitivity | 0.996  (0.004) | 1  (0) | 0.996  (0.008) | 0.988  (0.01) | 0.977  (0.02) |
|  | Specificity | 0.982  (0.007) | 0.99***  (0.001) | 0.986  (0.004) | 0.986  (0.004) | 0.969 (0.007) |
|  | TSS | 0.978  (0.009) | 0.99  (0.001) | 0.982  (0.007) | 0.974  (0.009) | 0.946 (0.014) |
| *Dumontia contorta* | AUC | 0.996  (0.001) | 0.997  (0.001) | 0.993  (0.005) | 0.999  (0.001) | 0.961 (0.002) |
|  | Sensitivity | 0.998  (0.001) | 0.995  (0.002) | 0.994  (0.005) | 0.996  (0.002) | 0.993 (0.004) |
|  | Specificity | 0.986  (0.002) | 0.984  (0.003) | 0.983  (0.002) | 0.992  (0.002) | 0.975 (0.004) |
|  | TSS | 0.984  (0.002) | 0.978  (0.002) | 0.978  (0.006) | 0.988  (0.001) | 0.967 (0.003) |
| *Sargassum muticum* | AUC | 0.997  (0.001) | 0.997  (0.001) | 0.997  (0.001) | 0.999  (0.001) | 0.975 (0.001) |
|  | Sensitivity | 0.994  (0.001) | 0.994  (0.003) | 0.994  (0.003) | 0.995  (0.003) | 0.974 (0.006) |
|  | Specificity | 0.987  (0.002) | 0.982  (0.001) | 0.983  (0.004) | 0.992  (0.001) | 0.985 (0.002) |
|  | TSS | 0.982  (0.002) | 0.975  (0.003) | 0.977  (0.004) | 0.987  (0.002) | 0.960 (0.007) |
| *Undaria pinnatifida* | AUC | 0.991  (0.006) | 0.984  (0.001) | 0.991  (0.001) | 0.999  (0.001) | 0.994 (0.002) |
|  | Sensitivity | 0.979  (0.015) | 0.97  (0) | 0.985  (0) | 0.997  (0.003) | 0.987 (0.022) |
|  | Specificity | 0.992  (0.002) | 0.996***  (0.001) | 0.988*  (0.004) | 0.989  (0.003) | 0.983 (0.011) |
|  | TSS | 0.971  (0.014) | 0.966  (0) | 0.973  (0.004) | 0.987  (0.006) | 0.970 (0.012) |
| *Acartia tonsa* | AUC | 0.992  (0.005) | 0.997  (0.001) | 0.995  (0.001) | 0.999  (0.001) | 0.977 (0.004) |
|  | Sensitivity | 0.988  (0.003) | 0.987  (0.003) | 0.983  (0.004) | 0.992  (0.001) | 0.919 (0.053) |
|  | Specificity | 0.972  (0.012) | 0.983  (0.003) | 0.983  (0.003) | 0.996  (0.001) | 0.955 (0.005) |
|  | TSS | 0.96  (0.013) | 0.97  (0.003) | 0.967  (0.003) | 0.988  (0.002) | 0.875 (0.051) |
| *Aurelia limbata* | AUC | 0.907  (0.024) | 0.898  (0.048) | 0.943  (0.014) | 0.925  (0.021) | 0.965 (0.012) |
|  | Sensitivity | 0.978  (0.007) | 0.813  (0.104) | 0.896  (0.036) | 0.875  (0.042) | 0.875 (0.125) |
|  | Specificity | 0.829  (0.066) | 0.932*  (0.02) | 0.904  (0.029) | 0.96***  (0.013) | 0.912 (0.004) |
|  | TSS | 0.766*  (0.027) | 0.745*  (0.106) | 0.801*  (0.042) | 0.835*  (0.029) | 0.787 (0.125) |
| *Mnemiopsis leidyi* | AUC | 0.994  (0.002) | 0.997  (0.001) | 0.995  (0.001) | 0.998  (0.001) | 0.962 (0.002) |
|  | Sensitivity | 0.994  (0.006) | 0.997  (0.002) | 0.976  (0.007) | 0.997  (0.001) | 0.945 (0.026) |
|  | Specificity | 0.986  (0.005) | 0.985  (0.001) | 0.989  (0.002) | 0.991  (0.001) | 0.951 (0.013) |
|  | TSS | 0.98  (0.006) | 0.982  (0.003) | 0.965  (0.008) | 0.989  (0.002) | 0.896 (0.014) |
| *Alexandrium tamarense* | AUC | 0.968  (0.012) | 0.99  (0.001) | 0.984  (0.001) | 0.997  (0.001) | 0.944 (0.004) |
|  | Sensitivity | 0.937  (0.017) | 0.955  (0.004) | 0.937  (0.017) | 0.979  (0.003) | 0.952 (0.036) |
|  | Specificity | 0.961  (0.009) | 0.963  (0.004) | 0.961  (0.009) | 0.983  (0.003) | 0.885 (0.018) |
|  | TSS | 0.898  (0.015) | 0.918  (0.004) | 0.898  (0.009) | 0.961  (0.002) | 0.838 (0.024) |
| *Dinophysis caudata* | AUC | 0.993  (0.001) | 0.996  (0.001) | 0.995  (0.001) | 0.998  (0.001) | 0.77  (0.004) |
|  | Sensitivity | 0.983  (0.002) | 0.983  (0.001) | 0.995  (0.001) | 0.991  (0.002) | 0.974 (0.001) |
|  | Specificity | 0.982  (0.004) | 0.981  (0.001) | 0.98  (0.003) | 0.99  (0.003) | 0.836 (0.008) |
|  | TSS | 0.965  (0.003) | 0.964  (0.001) | 0.976  (0.004) | 0.982  (0.002) | 0.810 (0.008) |
| *Dinophysis dens* | AUC | 0.997  (0.001) | 0.99  (0.007) | 0.994  (0.005) | 0.988  (0.009) | 0.992  (0.01) |
|  | Sensitivity | 0.988  (0.013) | 0.98  (0.02) | 0.986  (0.012) | 0.959  (0.029) | 0.917 (0.095) |
|  | Specificity | 0.986  (0.007) | 0.989**  (0.002) | 0.983  (0.003) | 0.985  (0.009) | 0.983 (0.002) |
|  | TSS | 0.974  (0.011) | 0.969  (0.019) | 0.969  (0.009) | 0.945  (0.027) | 0.899 (0.094) |
| *Gonyaulax polygramma* | AUC | 0.96  (0.007) | 0.958  (0.001) | 0.943  (0.001) | 0.994  (0.001) | 0.837 (0.007) |
|  | Sensitivity | 0.916  (0.005) | 0.921  (0.005) | 0.883  (0.003) | 0.972  (0.004) | 0.901 (0.012) |
|  | Specificity | 0.9  (0.019) | 0.887  (0.009) | 0.876  (0.006) | 0.973  (0.004) | 0.835  (0.01) |
|  | TSS | 0.816  (0.021) | 0.808  (0.005) | 0.759**  (0.003) | 0.945  (0.005) | 0.745 (0.016) |
| *Heterocapsa triquetra* | AUC | 0.991  (0.003) | 0.995  (0.001) | 0.992  (0.0010 | 0.998  (0.001) | 0.945 (0.005) |
|  | Sensitivity | 0.98  (0.003) | 0.984  (0.002) | 0.982  (0.002) | 0.994  (0.002) | 0.951 (0.015) |
|  | Specificity | 0.985  (0.003) | 0.984  (0.001) | 0.977  (0.002) | 0.991  (0.001) | 0.929 (0.007) |
|  | TSS | 0.966  (0.001) | 0.968  (0.001) | 0.959  (0.001) | 0.985  (0.002) | 0.880 (0.015) |
